# Blood flow coordinates collective endothelial cell migration during vascular plexus formation and promotes angiogenic sprout regression via *flt4*

**DOI:** 10.1242/dev.205424

**Published:** 2026-10-06

**Authors:** Yan Chen, Aaron M. Savage, Zhen Jiang, Ignacio Rodriguez-Pastrana, Myles Wilson, Hyejeong R. Kim, Paul C. Evans, Robert N. Wilkinson

**Affiliations:** ^1^ Department of Infection, Immunity and Cardiovascular Disease, University of Sheffield, UK; ^2^ School of Life Sciences, Medical School, University of Nottingham, Nottingham, NG72UH, UK; ^3^ Max Planck Institute for Heart and Lung Research, Department of Developmental Genetics, Bad Nauheim 61231, Germany; ^4^ German Centre for Cardiovascular Research (DZHK), Partner Site Rhine-Main, Bad Nauheim 61231, Germany; ^5^ Department of Biomedical Science, University of Sheffield, Sheffield, S10 2TN, UK; ^6^ Centre for Microvascular Research, William Harvey Research Institute, Queen Mary University of London, London, EC1M 6BQ, UK

**Keywords:** Zebrafish, Angiogenesis, Vascular remodelling, Haemodynamics, *flt4*, Vegfr3

## Abstract

Nascent vascular networks adapt to the increasing metabolic demands of growing tissues by expanding via angiogenesis. As vascular networks expand, blood vessels remodel, progressively refining vascular connectivity to generate a more haemodynamically efficient network. This is driven by interplay between endothelial cell (EC) signalling and blood flow. While much is known about angiogenesis, less is understood of how blood flow remodels vessels. Here, we employ the zebrafish sub-intestinal venous plexus (SIVP) to characterise blood flow-dependent remodelling. Using live imaging to track ECs, we show that blood flow controls SIVP remodelling by coordinating collective EC migration within the developing plexus. Blood flow opposes continuous ventral EC migration within the SIVP and is required for angiogenic sprout regression to support plexus growth. Sprout regression occurs by coordinated migration of ECs from non-perfused leading sprouts, which migrate against blood flow and incorporate into the sub-intestinal vein. Sprout regression remains compatible with low blood flow but requires *flt4* function under these conditions. Collectively, these studies reveal how blood flow sculpts a developing vascular plexus by coordinating EC migration and balancing vascular remodelling via *flt4*.

## Teaser

**Summary:** Live imaging in zebrafish reveals that blood flow coordinates directional endothelial cell migration during venous plexus remodelling, mediated by Flt4 under reduced-flow conditions.

## INTRODUCTION

During early development, cellular uptake of nutrients and elimination of metabolic waste must occur prior to the formation of specialised organs responsible for these functions. Consequently, the nascent vascular networks that mediate metabolic exchange undergo rapid adaptation to accommodate the dynamic metabolic demands of growing tissues. As vascular networks expand, they must optimise metabolic exchange while minimising resistance to blood flow. Evolution has therefore favoured mechanisms that enhance haemodynamic efficiency (Campinho et al., 2020). Angiogenic sprouting and vascular remodelling are balanced to maintain haemodynamic efficiency during network expansion. Remodelling occurs either by fusion or regression of angiogenic sprouts to form functional tubular vessels, or the pruning of redundant blood vessels (Ribatti and Crivellato, 2012). Endothelial cells (ECs) lining the inner surface of blood vessels play a central role in this process by responding dynamically to changes in their local environment (Udan et al., 2013). While most blood vessels develop from pre-existing patent vessels with stable blood flow, diverse mechanisms govern vascular remodelling and our understanding of the influence of blood flow on these processes remains incomplete. It is not clear whether vascular regression is driven by active signalling pathways, the withdrawal of survival cues, or a combination of both, or how this varies across vascular beds (Korn and Augustin, 2015).

The zebrafish sub-intestinal venous plexus (SIVP) forms bilaterally over the embryonic yolk sac and is structurally similar to the mammalian vitelline veins, which connect the embryo with extra-embryonic circulation in the yolk sac (Goi and Childs, 2016). Given its developmental plasticity, the SIVP has become a valuable model in which to investigate cellular mechanisms underlying vascular remodelling (Goi and Childs, 2016; Hen et al., 2015; Koenig et al., 2016; Lenard et al., 2015). The SIVP initially facilitates nutrient uptake from the yolk, later contributing to vascularisation of the larval digestive system (Isogai et al., 2001). The SIVP comprises the supra-intestinal artery (SIA), bilateral sub-intestinal veins (SIVs) and inter-connecting vessels (ICVs) which connect the SIA and SIVs (Fig. 1A). The SIVP originates from angioblasts in the ventral posterior cardinal vein, which give rise to both the SIA and the SIVs (Goi and Childs, 2016; Hen et al., 2015; Koenig et al., 2016). ECs emerge from the posterior cardinal vein and migrate ventrolaterally over the yolk. During this process, some ECs contribute to the forming SIV, whereas others are retained within the developing plexus and later contribute to ICVs and the SIA (Goi and Childs, 2016; Hen et al., 2015; Koenig et al., 2016). In mammals, progressive refinement of vascular networks by vessel pruning or regression involves dynamic migration and rearrangement of ECs (Franco et al., 2016, 2015; Udan et al., 2013). ECs can migrate within patent blood vessels during vascular network formation (Christ et al., 1990; Franco et al., 2015) leading us to hypothesise that coordinated EC motility is essential for SIVP remodelling and may be regulated by blood flow.

Vascular endothelial growth factor (VEGF) signalling controls diverse aspects of EC biology, including migration and survival (Akeson et al., 2010; Ferrara et al., 2003). VEGF ligands signal via their cognate receptor tyrosine kinase receptors VEGFR1-3, with VEGFR2 serving as the principal signalling receptor in mammalian blood ECs (Simons et al., 2016). Zebrafish Vegfr4/Kdrl and Vegfr2/Kdr are functionally analogous to mammalian VEGFR2 (Bussmann et al., 2008). ECs sense blood flow via junctional mechanosensory complexes, most notably the VE-cadherin, PECAM-1, VEGFR2/3 complex, which operates independently of VEGF ligands and is essential for arterial vascular remodelling (Coon et al., 2015; Tzima et al., 2005). Loss of endothelial VEGFR3/Flt4 triggers vascular remodelling in arteries (Baeyens et al., 2015; Tzima et al., 2005). Zebrafish *vegfaa* mutants exhibit severe defects in SIVP formation (Habeck et al., 2002; Koenig et al., 2016) and combined loss of *kdr* and *kdrl* similarly disrupts SIVP development (Habeck et al., 2002; Koenig et al., 2016). Sprout pathfinding within the SIVP depends on the guidance receptor Plexin D1, and plexus expansion is constrained by inhibition of bone morphogenetic protein (BMP) signalling (Goi and Childs, 2016). SIVP remodelling occurs via flow-dependent pruning and fusion of vessel branches (Hen et al., 2015; Lenard et al., 2015). However, how much this process depends on flow is unknown. Moreover, a systematic analysis of EC migration during SIVP formation is lacking and how blood flow and signalling pathways coordinate plexus remodelling remains unclear.

Here, we demonstrate that blood flow plays an essential role in coordinating EC migration during vascular plexus formation and remodelling. Through time-lapse imaging and quantitative analysis, we show that blood flow orchestrates collective EC migration throughout the developing SIVP and promotes regression of leading angiogenic sprouts to support plexus expansion. Our data suggest that sprout regression occurs by coordinated EC rearrangement between non-perfused sprouts and the lumenised SIV, driven by directed EC migration against blood flow. Using erythrocyte depletion to reduce circulation, we find that sprout regression can occur under reduced-viscosity flow conditions, but this process requires *flt4* function. Together, these findings provide a clear view of the complex cellular dynamics underlying vessel remodelling during vascular plexus formation and highlight the central role of EC responses to blood flow.

## RESULTS

### Blood flow controls SIVP remodelling by influencing EC distribution and promoting leading sprout regression

The SIVP develops via angiogenic sprouts from the posterior cardinal vein (PCV) by 30 h post-fertilisation (hpf), which migrate in a ventrolateral direction, bilaterally, over the surface of the yolk (Goi and Childs, 2016; Hen et al., 2015; Koenig et al., 2016). To determine the timing of SIVP development in relation to blood flow, we imaged the developing plexus using fluorescently labelled erythrocytes with *Tg(gata1a:*DsRed*)^sD2^* (Fig. 1A). Between 53 hpf and 55 hpf, blood flow entered the nascent SIVP anteriorly via the SIA (Fig. 1A-C″). The onset of SIVP perfusion progressed from anterior to posterior, and by 72 hpf the plexus was fully perfused with erythrocytes (Fig. 1D-G; Movie 1), coinciding with extensive vascular remodelling. Migrating angiogenic sprouts (hereafter referred to as leading sprouts) protruded from the ventral SIV and either fused with neighbouring leading sprouts or regressed, with ECs re-integrating into the SIV by 70 hpf (Fig. 1H-J, arrows; Movies 1 and 2, arrowheads). We note that cells labelled by *Tg(gata1a:*DsRed*)^sD2^* are present outside of vessels (Fig. 1 and subsequent figures). Ectopic expression of *Tg(gata1a:*DsRed*)^sD2^* has been previously reported (Andreana et al., 2025; Daetwyler et al., 2019).

**Fig. 1.**
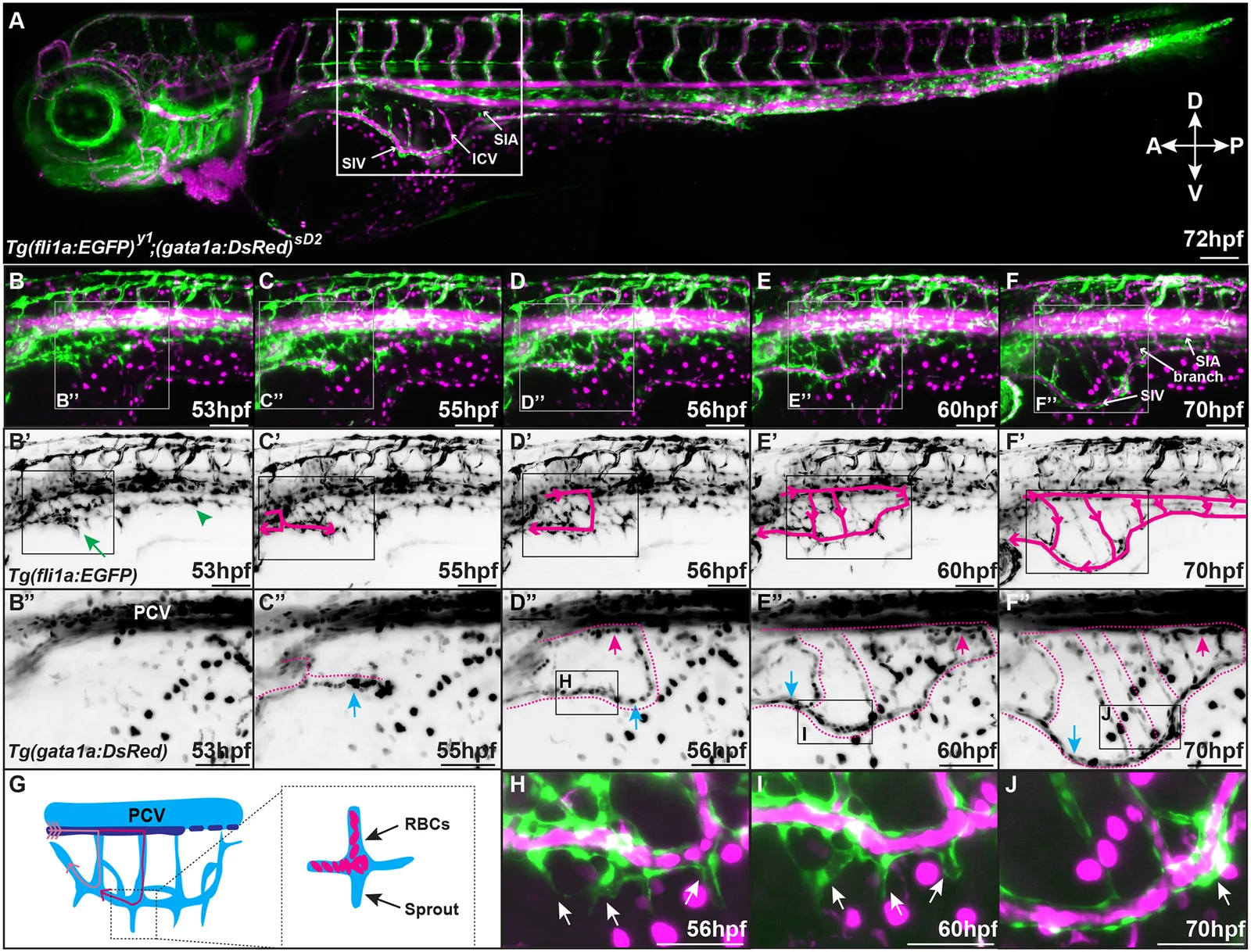
**SIVP perfusion is coincident with leading sprout regression.** (A-F″) Representative images from a time lapse (Movie 1) between 53 and 70 hpf showing perfusion of the SIVP. *Tg(fli1a:EGFP)^y1^* labels ECs (green) and *Tg(gata1a:DsRed)^sD2^* labels erythrocytes (magenta). The region highlighted in A is displayed in B-F, and the region highlighted in B-F is enlarged in B″-F″. B″-F″ displays the path of circulating erythrocytes (pink dotted line). Circulation enters the anterior SIVP between 53 hpf (B-B″) and 55 hpf (C-C″) and perfuses the SIV from anterior to posterior as it develops (D-F″). Blood enters the SIVP from the SIA (D′-F″, magenta arrows), an extension of the upstream anterior mesenteric artery, through the SIVP branches, drains into the SIV (C″-F″, blue arrows) and exits the SIV via the hepatic portal vein. (G) Schematic of SIVP perfusion. Direction of perfusion is highlighted by arrows; the segment highlighted in light pink was perfused first, followed by the segment highlighted in magenta. (H-J) Leading sprouts are observed to regress and undergo anastomosis in the presence of blood flow (arrows). A, anterior; D, dorsal; ICV, inter-connecting vessel; P, posterior; PCV, posterior cardinal vein; RBC, red blood cells; SIA, supra-intestinal artery; SIV, sub-intestinal vein; V, ventral. Scale bars: 100 μm (A-F′); 50 μm (B″-F″,H-J).

Remodelling of leading sprouts coincided with the onset of SIV blood flow, with new leading sprouts rarely forming after erythrocyte circulation through the SIVP. Indeed, most existing sprouts underwent regression, and regression displayed a posterior bias, with anterior sprouts taking longer to regress (Fig. S1). Therefore, we defined this period as the sprout regression phase. It is important to note that plasma flow may enter the SIVP before erythrocytes are detectable. However, since erythrocytes make a major contribution to blood viscosity and therefore to the magnitude of wall shear stress, we focused our analysis on erythrocyte flow as a practical readout of haemodynamic input. To determine whether this process depends on blood flow, we inhibited cardiac contraction and circulation using a morpholino targeting cardiac troponin T type 2a (*tnnt2a*) and examined SIVP morphology (Fig. 2). Although the overall size of the SIVP was unchanged in *tnnt2a* morphants compared to controls, (Fig. 2A-B′,C), its morphology was altered (Fig. 2A,B). Specifically, morphants exhibited fewer vascular loops (Fig. 2A′,B′ asterisks; 2D), reduced dorsoventral plexus length (Fig. 2A′,B′,E) and increased length (Fig. 2A′,B′,E) and number of leading sprouts (Fig. 2A″,B″,E,F). While total SIVP EC number was not significantly different (Fig. 2G), EC distribution was affected (Fig. 2A″,B″,H,I). Leading sprouts in morphants contained more ECs (Fig. 2A″,B″,H,I), whereas SIVP branches had significantly fewer. However, EC numbers in the ventral region of the SIVP (within the SIV), were not significantly altered (Fig. 2A″,B″,I).

**Fig. 2.**
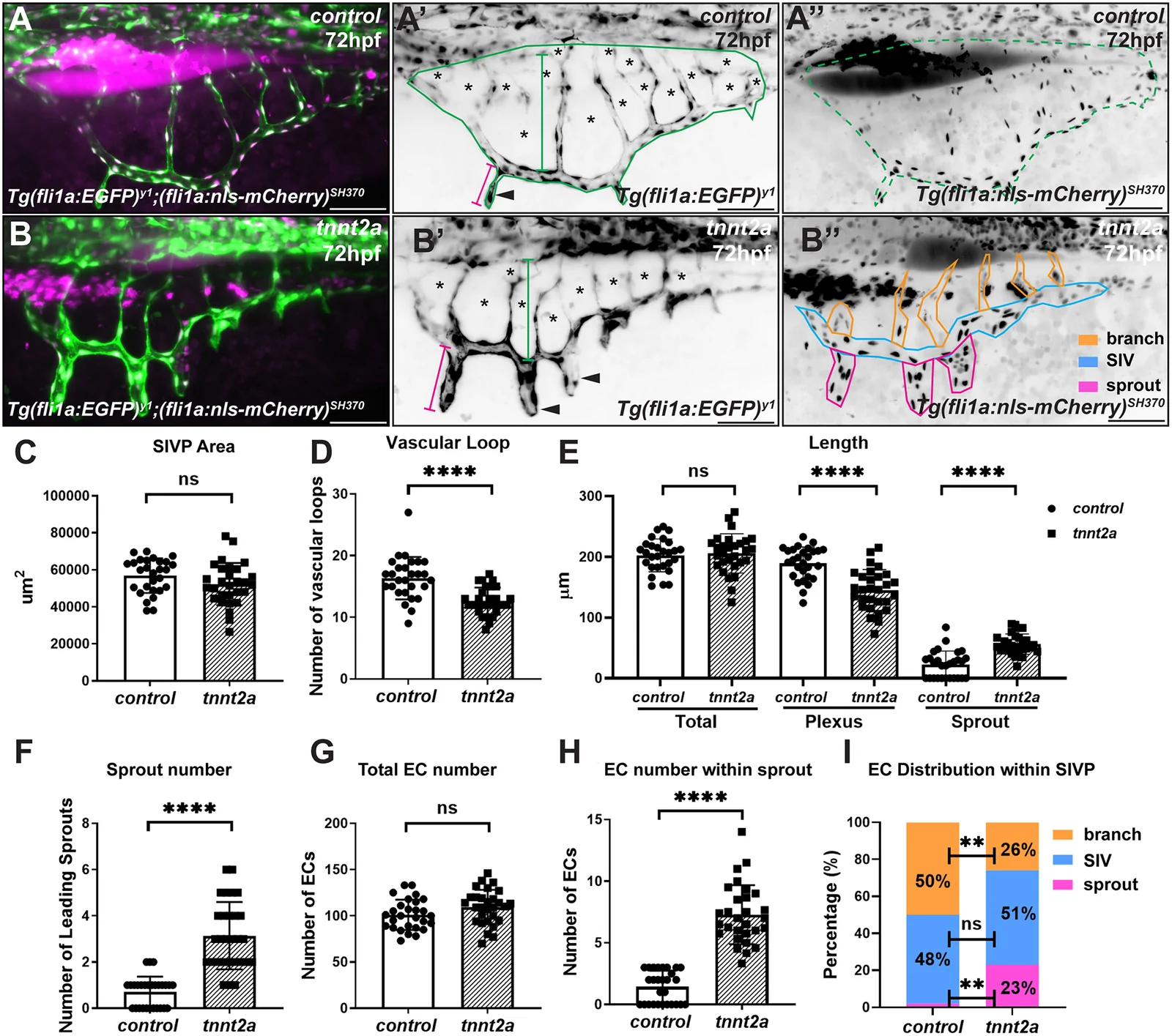
**Blood flow controls morphology and EC distribution of the SIVP.** (A-I) Comparison of SIVP morphology at 72 hpf in the presence (control morphants; A-A″) or absence (*tnnt2a* morphants; B-B″) of blood flow. No significant difference in plexus area (highlighted in green in A′,A″) was observed in the presence or absence of blood flow (C). Embryos without blood flow displayed fewer vascular loops (A′, asterisks; D). Total length of the SIVP plexus was not altered in the presence or absence of blood flow (B′, green line; E); however, *tnnt2a* morphant embryos displayed reduced length of the SIVP basket (B″, blue line; E) and increased sprout length (B′, magenta line; E). The number of leading sprouts (A′,B′, arrowheads) were increased in *tnnt2a* morphant embryos (B″, highlighted in magenta; F). The total number of ECs in the SIV was not altered by the flow status of the plexus (G); however, leading sprouts contained increased numbers of ECs in *tnnt2a* morphant embryos (B″, highlighted in magenta; H). In the absence of blood flow, SIV branches contained fewer ECs (B″, highlighted in orange; I). ****P≤0.0001; **P≤0.01 (unpaired *t*-test). ns, not significant (*P*≥0.05). Control embryos, *n*=28; *tnnt2a* embryos, *n*=29 (3 replicates). Graphs show mean±s.d. Scale bars: 50 μm.

To control for potential morpholino off-target effects, we generated G0 *tnnt2a* CRISPR mutants (Wu et al., 2018) and assessed SIVP morphology. Mosaic *tnnt2a* mutants phenocopied the defects observed in morphants (Fig. S2A,B, arrows; S2C-I; Fig. 2), confirming that these changes were specific to *tnnt2a* inhibition. However, both morpholino or G0 mutagenesis inhibit *tnnt2a* from early development. To test whether blood flow is required specifically during SIVP remodelling, we employed acute pharmacological inhibition of cardiac contraction, using nifedipine treatment during SIVP remodelling between 56 and 72 hpf, when SIVP perfusion by erythrocytes begins. Acute nifedipine treatment abolished flow (Fig. S3A). Nifedipine-treated embryos displayed increased leading sprout number, leading sprout length and total plexus length, while total SIVP area was unchanged (Fig. S3B-F). Unlike *tnnt2a* knockdown, acute nifedipine treatment reduced total SIVP EC number. However, similar to *tnnt2a* knockdown, nifedipine treatment increased the relative number of ECs in leading sprouts compared with branches and the SIV (Fig. S3G-I). This difference may reflect the abrupt timing of flow cessation or additional pharmacological effects of calcium-channel inhibition. Nonetheless, both manipulations caused leading sprout retention, supporting a role for blood flow during sprout regression. Together, these findings suggest that blood flow influences EC distribution within the developing venous plexus and that blood flow promotes sprout regression. While *tnnt2a* morphants retained more leading sprouts by 72 hpf (Fig. 3D-F), leading sprouts regressed in the presence of flow, with their ECs integrating into the SIV (Fig. 3A-C, arrows; Movie 3). Anastomosis of leading sprouts was rare and occurred independently of blood flow (Fig. 3A, asterisks; 3G,H; Movie 3). Overall, these data indicate that blood flow contributes to SIVP remodelling, in part by promoting leading sprout regression.

**Fig. 3.**
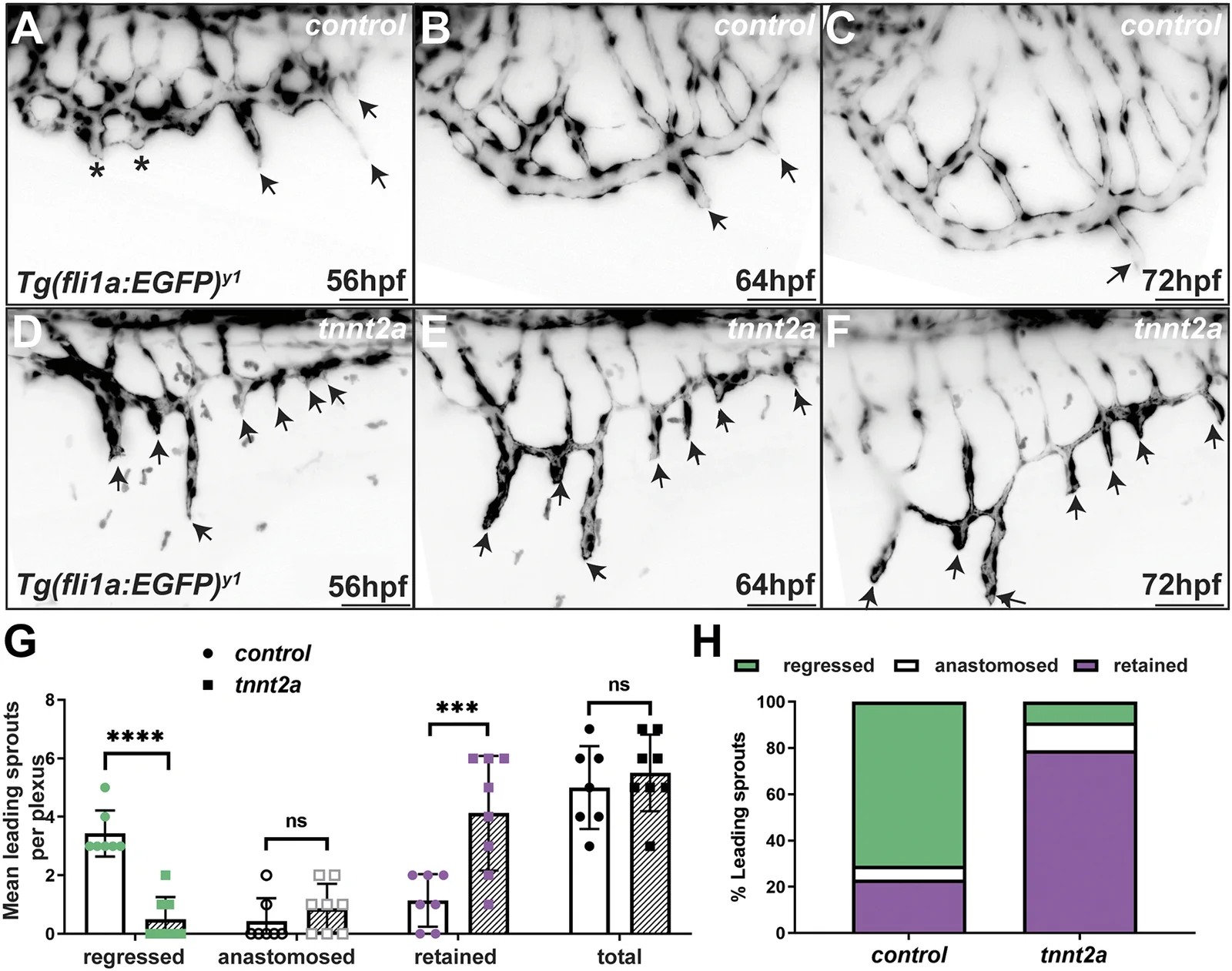
**Blood flow promotes regression of SIVP leading sprouts.** (A-F) Still images taken from time-lapse movies (Movie 3) showing SIVP development from 56 to 72 hpf in the presence (A-C) and absence (D-F) of flow. (A) In control animals, before blood flow enters the plexus, some angiogenic sprouts from the primary SIV anastomose (asterisks) to form vascular loops, while others lead at the migration front (arrows). (B,C) In controls, leading sprouts regressed following the onset of blood flow and sprout ECs became incorporated into the SIV (arrows). (D-F) In the absence of flow, leading sprouts failed to regress (D-F, arrows). (G,H) Leading sprout regression was significantly reduced in *tnnt2a* morphant embryos and, thus, the number of sprouts present at 72 hpf was increased in comparison to controls. ****P≤0.0001; ***P≤0.001 (unpaired *t*-test). ns, not significant (*P*≥0.05). Control morphants, *n*=8; *tnnt2a* morphants, *n*=7 (3 replicates). Graphs show mean±s.d. Scale bars: 50 μm.

### Blood flow controls EC migration but not proliferation within the developing SIVP

Although flow status did not affect the total number of ECs within the SIVP (Fig. 2G), the altered spatial distribution of ECs (Fig. 2H,I) suggested that flow might affect local rates of proliferation, apoptosis or migration. To determine how blood flow affects EC redistribution during SIVP remodelling, we tracked individual EC nuclei from 56 to 70 hpf in control and *tnnt2a* morphants (Fig. 4A-C; Movies 4-6). ECs were classified according to their initial position at 56 hpf as tip cells, SIV cells or branch cells, and their final position was used to quantify transitions between vessel compartments (Fig. 4D; Tables S3 and S4). These movies show representative examples of the cell behaviours, quantified in Fig. 4D and Tables S3 and S4, including dorsal movement of tip-derived cells into the SIV in controls and retention of tip cells within leading sprouts in *tnnt2a* morphants.

**Fig. 4.**
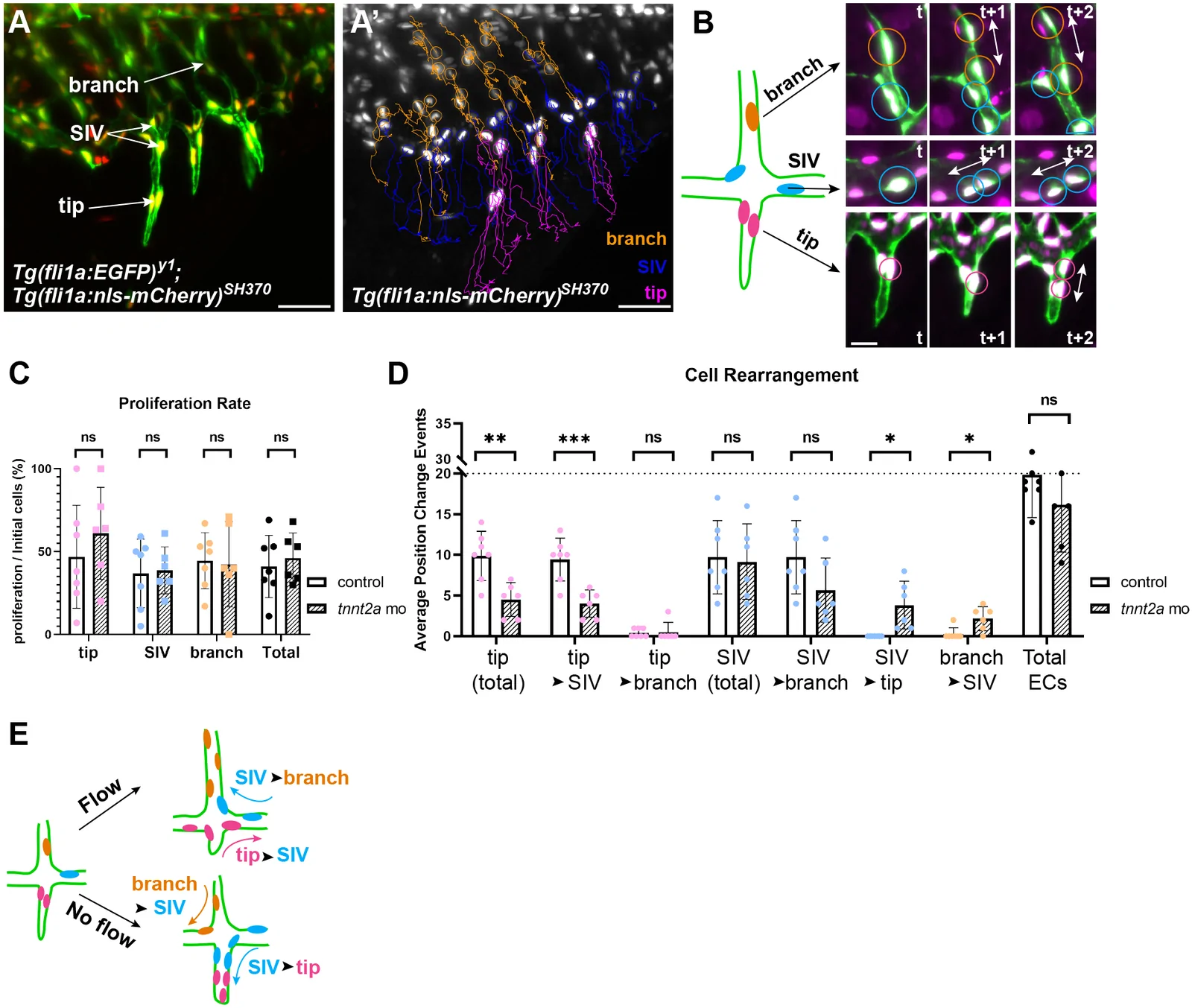
**Blood flow coordinates EC migration but not proliferation within the developing SIVP.** (A-B) ECs in the SIVP were divided into three groups depending upon their initial position at 56 hpf: SIVP branch cells (orange) within branches, SIV cells within the ventral SIV (blue) and tip cells within leading sprouts (magenta). Examples of EC proliferation in subsequent timepoints are highlighted (B). t, t+1 and t+2 denote three consecutive time points in the time-lapse sequence. The direction of cell division is parallel to the long axis of the vessel (B). (C) EC proliferation rate (cell division events/initial number of ECs) did not differ in total or between groups in the presence or absence of flow. (D) There was a substantial reduction in tip cell contribution to SIVs in *tnnt2a* morphants, indicating that flow is required for dorsal tip cell rearrangement. In addition, there was an increase in ventral migration of SIV ECs into leading sprouts in the absence of flow. Most branch cells remained in the branches under flow; however, more branch ECs migrated ventrally to contribute to the SIV without blood flow. (E) Schematic of differing EC migration in the presence and absence of blood flow. Under normal flow, leading sprout tip cells migrate dorsally and incorporate into the SIV, promoting sprout regression, while SIV ECs contribute to branch remodelling. In the absence of flow, EC rearrangement is altered, with increased retention of tip cells in leading sprouts and movement of SIV ECs into sprouts, consistent with impaired sprout regression. ***P≤0.001; **P≤0.01; *P<0.05 (unpaired *t*-test). ns, not significant (*P*≥0.05). Control morphants, *n*=7; *tnnt2a* morphants, *n*=6 (3 replicates). Graphs show mean±s.d. Scale bars: 50 μm (A,A′); 25 μm (B).

Flow status of the developing plexus did not affect endothelial proliferation rates (Fig. 4C), and no apoptosis was observed, making differential proliferation or apoptosis unlikely to account for altered EC distribution in *tnnt2a* morphants (Fig. 2I). However, the frequency of differential ECs contribution to neighbouring EC subsets was altered (Fig. 4D). In the presence of blood flow, most leading sprouts consisted of a pair of endothelial tip cells positioned in parallel to each other, consistent with previous reports (Hen et al., 2015). When tip cells divided in the presence of blood flow (Fig. 4B), the dorsal daughter cell rapidly migrated dorsally in most cases to contribute to the SIV (Movie 6, arrows). This left the paired tip cells within the sprout, suggesting that the migrating daughter cell adopted a stalk identity (Tables S2, S3). In rare cases, with blood flow present, daughter cells that migrated dorsally from leading sprouts to the SIV were later observed contributing to SIVP branches by 72 hpf (Fig. 4D). ECs were not seen migrating into leading sprouts under normal flow conditions. However, occasional bending and transient repositioning of EC nuclei at the boundary between the SIV and leading sprouts were observed, suggesting limited plasticity at this interface. In contrast, in *tnnt2a* morphants fewer tip cells migrated dorsally to the SIV, with most tip cells remaining within leading sprouts by 72 hpf, aligning with our previous observations (Fig. 2B″,I; Fig. S1I; Movies 4, 5). Notably, in *tnnt2a* morphants, some SIV ECs migrated ventrally into leading sprouts (Fig. 4D) but did not appear to acquire tip cell identity.

ECs within SIVP branches comprise approximately half of the total plexus EC population by 72 hpf, yet they rarely change position in the presence of blood flow (Movie 4; Table S4). Fewer than 1% of ECs in branches contributed to cellular rearrangement during SIVP development (Fig. 4D; Table S4). In contrast, ECs from branches were significantly more likely to migrate into the SIV in *tnnt2a* morphants (Fig. 4D; Movie 5; Table S4). Although the overall number of EC positional changes was not significantly different between control and *tnnt2a* morphants (Fig. 4D), the direction of migration varied. ECs tended to migrate dorsally in the presence of flow, while ventral migration was more common in plexuses that developed without flow, resulting in an increased tendency for ECs to adopt and maintain tip and stalk cell behaviour (Fig. 4D). Collectively, these data suggest that blood flow is required to guide the directional rearrangement of ECs during SIVP development.

In the presence of blood flow, ECs within the SIVP exhibited distinct migratory behaviours depending upon their location. While the plexus expanded and ECs migrated ventrally, blood flow promoted dorsal migration, likely supporting coordinated plexus growth (Fig. 4E). In the absence of blood flow, ECs showed persistent ventral movement, leading to distended SIVP branches with fewer cells (Fig. 3C,F) and elongated leading sprouts with accumulated ECs displaying tip cell behaviour (Fig. 4E).

### Blood flow coordinates the direction of EC migration during SIVP development

To investigate whether blood flow influences EC migration, we focused on the most ventral ECs within each SIVP, particularly those located in the SIVs and leading sprouts. Given the semi-circular curvature of the SIVP, ECs in the anterior and posterior of the SIVP migrate shorter distances than ventral ECs in the central region of the plexus. We tracked ECs from 56 hpf onward and analysed their movement between consecutive timepoints, referred to as migration steps.

ECs near the most ventral sprout were classified as either SIV ECs (blue) or tip cells (magenta) based on initial positions at 56 hpf (Fig. 5A,B), and migration steps were recorded at 30-min intervals. While total EC migration distances did not differ significantly between controls and *tnnt2a* morphants (Fig. 5C), the meandering index (total distance/displacement ratio), was significantly reduced in tip cells in *tnnt2a* morphants (Fig. 5D). A meandering index closer to 1 indicates a straighter migration path, while values >1 reflect more-tortuous paths. Thus, in the absence of blood flow, tip cells migrated more directly in a ventral direction, with reduced dorsal rearrangement toward the SIVs (Fig. 5D). In contrast, the meandering index of ECs within the SIV was unaffected by flow status (Fig. 5E). Migration velocity of both tip and SIV ECs gradually decreased in the presence of blood flow, whereas no significant reduction was observed in *tnnt2a* morphants (Fig. 5F), suggesting that flow may act to slow EC migration and promote vessel stability. Additionally, EC nuclear spacing was reduced in *tnnt2a* morphants (Fig. 5G), consistent with increased EC density in the absence of flow (Fig. 2B″).

**Fig. 5.**
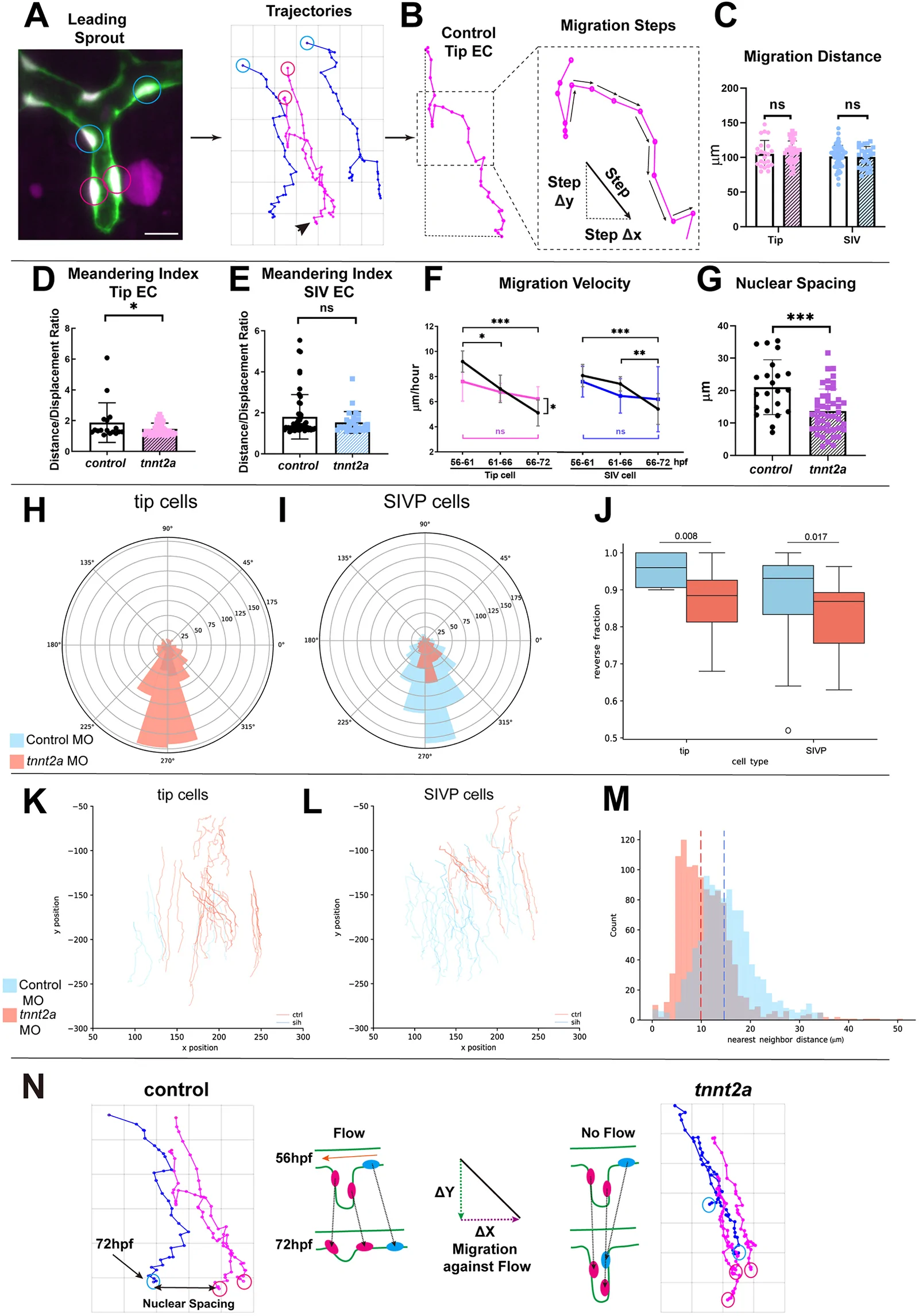
**Blood flow controls EC migration direction during leading sprout regression.** (A) Examples of ECs within the SIV (blue) or tip cells (magenta) tracked in proximity to leading sprout and example migration trajectories. Arrowhead in trajectory map indicates the final recorded position of migrating EC. (B) Migration ‘steps’ are defined as the movement of ECs between two consecutive timepoints (arrows). (C) Migration distance (the sum of step displacements) of SIV ECs or tip cells was unaffected by flow status of the SIVP. (D,E) Meandering index was significantly reduced in tip cells in *tnnt2a* morphants (D) but was not significantly different in SIV ECs (E). (C-E) Each dot represents a migration track of a single EC. *P≤0.05 (unpaired *t*-test). ns, not significant (*P*≥0.05). Control, *n*=79 tracks from 5 embryos; *tnnt2a*, *n*=64 tracks from 5 embryos. Tip cells: control, *n*=23 tracks; *tnnt2a*, *n*=37 tracks. SIV cells: control, *n*=56 tracks; *tnnt2a*, *n*=27 tracks. (F) Migration velocity of tip or SIV cells gradually reduced in the presence of flow (black lines) but was not significantly altered in *tnnt2a* morphants (coloured lines). Average speed of every step of tip cells or SIV cells from six control or *tnnt2a* embryos. *x*-axis presents distinct 5-h temporal bins during which cell migration was tracked. *P≤0.05; **P≤0.01; ***P≤0.001 (unpaired *t*-test). ns, not significant (*P*≥0.05). (G) Nuclear spacing was significantly reduced in *tnnt2a* morphants indicating increased EC crowding within the SIVP. Control, *n*=22 from 6 embryos; *tnnt2a*, *n*=46 from 6 embryos. ***P≤0.001 (unpaired *t*-test). (H,I) Rose plots displaying coordinates and relative extent of tip and SIVP cell migration. *n*=383 steps from 4 embryos; *tnnt2a*, *n*=860 steps from 4 embryos. (J) Analysis of reverse migration of both tip and SIV cells in both conditions demonstrates reduced reverse migration in *tnnt2a* morphants. Box limits represent upper and lower quartiles, horizontal line represents the median and whiskers represent minimum and maximum values. Unpaired *t*-tests. (K,L) Cell trajectory plots showing movement of tip and SIVP cells over time. (M) Nearest neighbour analysis of all SIV cells showing cell clustering in *tnnt2a* morphants. Dashed lines indicate the median nearest-neighbour distance for each condition. (N) Schematic of EC migration within the SIVP during sprout regression. In the presence of flow, SIV cells migrate laterally to accommodate dorsally migrating tip cells as leading sprouts regress. In the absence of flow, SIV ECs migrate more anteriorly, and tip cells migrate ventrally leading to elongated sprouts and regression failure. All data were generated from three biological replicates. Graphs show mean±s.d. Scale bar: 25 μm. All experimental time lapses were captured between 56 and 72 hpf.

We defined migration steps as a relative change in position in both *x* and *y* directions over a 30-min interval (Fig. 5B) and used this information to generate rose plots. This analysis preserved temporal information and revealed distinct effects of blood flow on tip cell and SIV EC migration. Tip cells in *tnnt2a* morphants displayed increased ventral migration compared with controls (Fig. 5H; Fig. S4), consistent with sprout extension and impaired regression. In contrast, SIV ECs displayed greater ventral migration in control embryos than in *tnnt2a* morphants (Fig. 5I; Fig. S4). Both tip cells and SIV ECs showed increased reverse migration in control embryos compared with *tnnt2a* morphants (Fig. 5J), indicating that blood flow promotes directional endothelial rearrangements during SIVP remodelling. Furthermore, in *tnnt2a* morphants, tip cells and SIV ECs remained more closely clustered during migration (Fig. 5K,L; Fig. S5), with tip cells displaying a reduced nearest-neighbour distance (Fig. 5M). Since tip cells normally migrate in pairs prior to dorsal sprout regression (Fig. 5N), the persistence of tip cell clustering in *tnnt2a* morphants is consistent with failed sprout regression.

Together, these data suggest that blood flow redirects endothelial migration during SIVP remodelling. As the plexus expands, ECs continue to move ventrally; however, once flow is established, tip cells undergo reverse/dorsal migration and incorporate into the SIV, while SIV ECs rearrange to accommodate sprout regression. In *tnnt2a* morphants, this flow-associated re-direction is impaired such that tip cells migrate more persistently ventrally, remain clustered within unresolved sprouts, and fail to re-integrate efficiently into the SIV. This suggests blood flow does not simply suppress endothelial motility, but biases ongoing EC migration to coordinate leading sprout regression.

### Intracellular actin rearrangements occur during sprout regression

To explore EC rearrangement during sprout regression, we visualised endothelial actin cytoskeleton using an EC-specific actin nanobody line, *Tg(fli1a:AC-TagRFP)^SH511^* (Savage et al., 2019) (Fig. S6; Movie 7). Leading sprouts consisted of paired tip cells with dorsal membranes connected to the SIV lumen (Fig. S6A-D). As regression progressed, both tip cells appeared to migrate over each other toward the SIV. The leading tip cell (Fig. S6E-H, green) re-oriented its nucleus from a dorsoventral to lateral orientation, with most of its membrane incorporated into the perfused SIV. The adjacent tip cell (Fig. S6I-L, pink) followed, retracting its membrane and re-orienting its nucleus horizontally upon reaching the SIV. Meanwhile the leading tip cell maintained its position, possibly to preserve vessel integrity. During this process, an F-actin-positive focus was observed at the ventral membrane of the trailing tip cell (Fig. S6H), which appeared to form a transient ring-like structure that expanded and eventually closed as the membrane merged with the main vessel (Fig. S6M-P, pink), suggesting a role for dynamic actin reorganisation. To help distinguish active endothelial rearrangement from passive incorporation into the SIV wall, we compared nuclear eccentricity in regressing sprout ECs and neighbouring SIV ECs (Fig. S6U), building on previous studies showing that vascular regression and pruning involve coordinated EC migration and rearrangement (Franco et al., 2015; Lenard et al., 2015). SIV ECs displayed more elongated nuclei, whereas reverse-migrating sprout ECs had more rounded nuclei (Fig. S6U). Although nuclear morphology alone does not prove active migration, the absence of SIV-like elongation in regressing sprout ECs argues against a simple passive-pulling model. Together with associated actin remodelling during sprout regression (Fig. S6H,M-P), these data support the interpretation that leading sprout regression involves active endothelial rearrangement.

### Leading sprout regression occurs under conditions of reduced blood flow

EC rearrangement from regions of low to high shear stress has been implicated in vessel pruning during vascular development (Franco et al., 2015). Shear stress experienced by ECs is directly influenced by haemodynamic flow and blood viscosity, and inversely proportional to the cube of the vessel radius (Heil and Schaper, 2004). We next examined whether reduced blood viscosity is compatible with normal sprout regression. We titrated *gata1a* morpholino to deplete circulating erythrocytes by an average of 87% (Fig. 6A-C, arrowheads). Unlike *tnnt2a* loss of function (Fig. 2, Fig. S1), *gata1a* morphants showed no major changes in overall SIVP morphology (Fig. 6A,B). ICVs remained connected to the SIA, and the vascular loop number was unchanged (Fig. 6D). However, many ICVs were not patent in *gata1a* morphants (Fig. 6B), likely due to reduced erythrocyte flow, which may have contributed to the smaller plexus size observed in these larvae (Fig. 6E,F). Surprisingly, the number of leading sprouts was not significantly different between control and *gata1a* morphants (Fig. 6G), indicating that sprout regression can occur under reduced-viscosity flow conditions. These data align with models proposing a low endothelial shear stress set point (Baeyens et al., 2015).

**Fig. 6.**
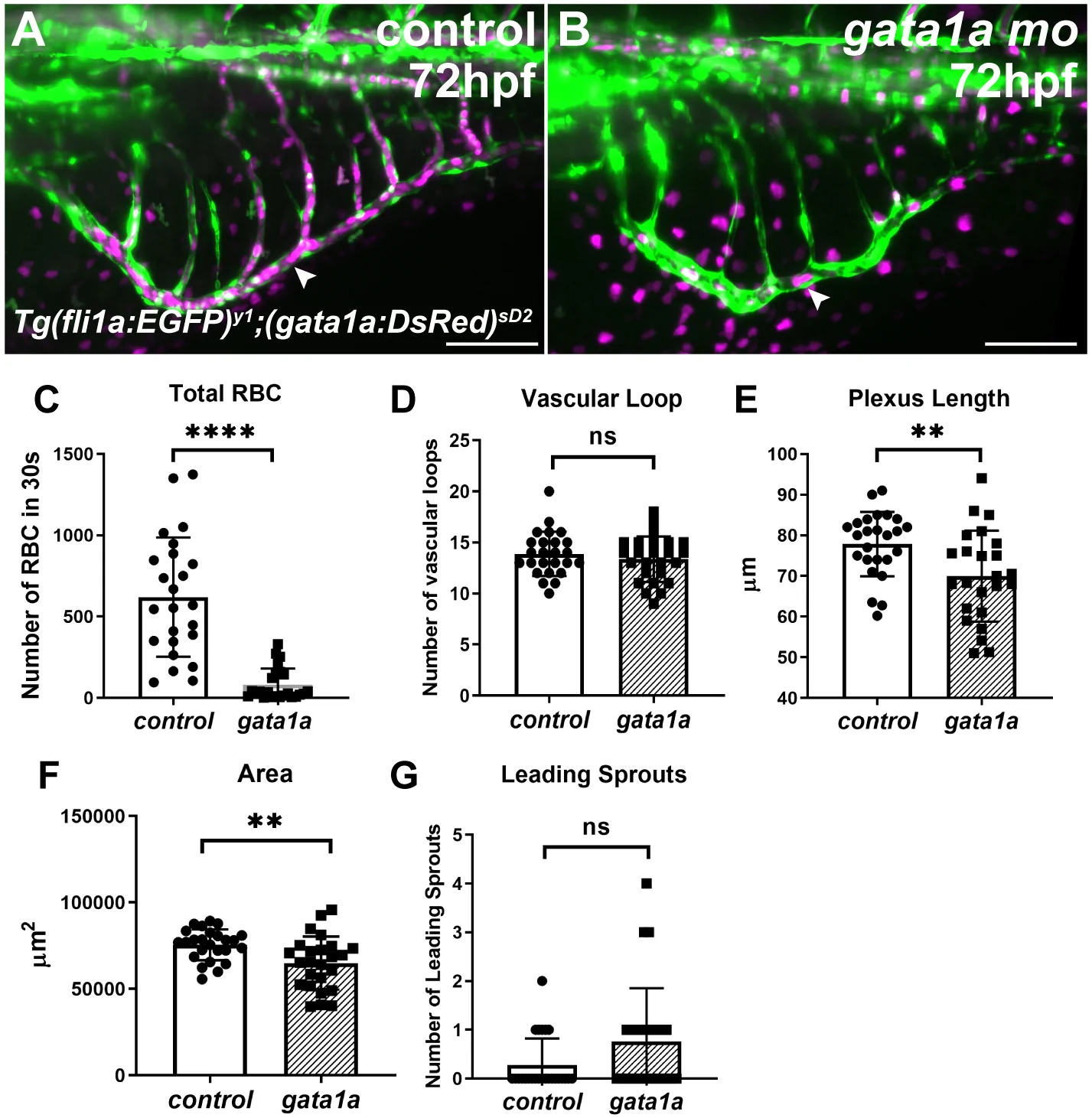
**Leading sprout regression occurs under conditions of reduced blood flow.** (A-G) Leading sprouts undergo normal regression in *gata1a* morphants (B) in comparison to controls (A) despite a substantial reduction of circulating erythrocytes in *gata1a* morphants (C). The frequency of vascular loops did not differ between *gata1a* morphants and controls (D), but plexus length (E) and area (F) was reduced in *gata1a* morphants. The frequency of leading sprouts was not significantly altered in *gata1a* morphants (G). ****P≤0.0001; **P≤0.01 (unpaired *t*-test). ns, not significant (*P*≥0.05). Control morphants, *n*=24; *gata1a* morphants, *n*=24 (3 replicates). Graphs show mean±s.d. Scale bars: 50 μm. RBC, red blood cell.

### Leading sprout regression is dependent on *flt4* under low blood flow conditions

Zebrafish *kdrl* and *kdr* are individually and cooperatively required for normal SIV formation (Habeck et al., 2002; Koenig et al., 2016). Loss of *flt4* in zebrafish arteries triggers inward remodelling (Baeyens et al., 2015). Additionally, Flt1 (VEGFR1), inhibits excessive venous sprouting in the CNS (Krueger et al., 2011; Matsuoka et al., 2016; Wild et al., 2017) and may act as a decoy receptor to limit angiogenesis (Hiratsuka et al., 1998; Park et al., 1994; Zygmunt et al., 2011). Increased sprouting in the SIVP has previously been reported in *flt1* morphants (Hen et al., 2015); however, the flow status of the SIVP was not examined. We hypothesised that blood flow may regulate *flt1* expression in the SIVP to facilitate regression.

We examined VEGF receptor expression in the SIVP under normal and flow-deficient conditions. Since SIVP signal was low relative to background yolk staining, these *in situ* data were interpreted qualitatively. Membrane-bound *flt1* (*mflt1*) was undetectable in the SIVP at either 56 hpf when sprout regression typically begins (Fig. S7A,B), or 72 hpf, when regression is complete (Fig. S7C,D), regardless of flow status. Similarly, soluble *flt1* (*sflt1*) was not detected in the SIVP at 56 hpf in either condition (Fig. S7E,F). However, *sflt1* was detected in the SIVP in a subset of *tnnt2a* morphants at 72 hpf (Fig. S7G,H, arrow), but absent in controls, suggesting that blood flow may suppress *flt1* expression in the SIVP. This pattern was consistent in the cerebral vasculature where *sflt1* expression was present and unaffected by flow at 56 hpf (Fig. S7I,J, arrows), but increased by 72 hpf in morphants (Fig. S7K,L, arrows). Since *flt1* was not expressed in the SIVP under normal flow conditions, it is unlikely to be required for sprout regression. Supporting this, sprout regression was normal in *flt1* mutants (Fig. S8), indicating that *flt1* is dispensable for this process.

Kdrl is essential for proper formation and perfusion of the SIVP (Habeck et al., 2002), and VEGFR3/Flt4 has been proposed to regulate endothelial shear stress response (Baeyens et al., 2015). Both *kdrl* and *flt4* were broadly expressed throughout the developing SIVP (Fig. S9, arrowheads) and their expression persisted in leading sprouts which failed to regress in *tnnt2a* morphants (Fig. S9I,F,L, arrowheads). *kdrl* expression was unaffected by blood flow status at all examined stages (Fig. S9A-F, arrowheads), whereas *flt4* expression was elevated in the ventral SIVP of *tnnt2a* morphants at 72 hpf compared to controls (Fig. S9K,L, arrowheads). Interestingly, *vegfaa* expression did not show an obvious change in spatial pattern in the absence of blood flow (Fig. S10), suggesting that the increased sprouting observed under flow-deficient conditions is unlikely to result from gross changes in *vegfaa* expression.

Since loss of *kdrl* disrupts SIVP perfusion entirely (Habeck et al., 2002; Koenig et al., 2016) we focused on *flt4* morphants. Knockdown of *flt4* did not impair sprout regression and was compatible with normal remodelling, similar to *gata1a* morphants (Fig. S11A,C,E, arrows). However, simultaneous knockdown of *flt4* and *gata1a* resulted in incomplete sprout regression by 72 hpf compared to controls (Fig. S11D, arrowheads; S11E). Importantly, *gata1a* knockdown did not affect expression of *vegfc*, suggesting that incomplete sprout regression was not due to increased *vegfc* expression adjacent to the SIVP (Fig. S12).

Given the known discrepancies between morpholino and mutant phenotypes (Kok et al., 2015), we generated a new *flt4* allele, *flt4^qmc316^*, using CRISPR mutagenesis. This allele contains a frameshift mutation in exon 3 at amino acid 83, resulting in a premature truncation at amino acid 94 (Fig. S13A). Consistent with impaired Flt4 function, *flt4^qmc316^* mutants displayed no gross morphological defects by 5 days post-fertilisation (dpf) (Fig. S13B,C) but showed severe lymphatic defects. These included reduced meningeal lymphatic ECs (Fig. S13E, yellow asterisk), loss of the otolithic facial lymphatic vessel (Fig. S13E′, white asterisk), medial facial lymphatic vessel (Fig. S13E′, red asterisk) and lateral facial lymphatic vessel (Fig. S13E′, green asterisk), and absence of the thoracic duct (Fig. S13E″). However, some craniofacial lymphatic structures were retained, including the facial lymphatic valve, and mutants did not develop overt oedema by 5 dpf (Fig. S13E, blue arrowhead). Together with comparison to previously described *flt4* mutants (Hogan et al., 2009b; Shin et al., 2016), this suggests that *flt4^qmc316^* is likely to be a hypomorph. Importantly, since *flt4^qmc316^* mutants do not display embryonic oedema up to 5 dpf, this allowed us to study impaired *flt4* function without disrupting blood flow in the SIVP. We therefore injected control or titrated *gata1a* morpholinos into embryos from a *flt4^qmc316^* heterozygous incross. Under normal flow conditions, there was no significant difference in leading sprout number between wild-type, heterozygous and homozygous siblings (Fig. 7A-D). However, *gata1a* knockdown in *flt4^qmc316^* significantly increased the number of non-regressing sprouts compared to heterozygous and wild-type siblings (Fig. 7E-H), indicating that *flt4* function is required for effective sprout regression under reduced reduced-viscosity flow conditions. We next investigated whether *flt4^qmc316^* mutants displayed pre-existing defects in SIVP development that could influence sprout regression under reduced-viscosity flow conditions and analysed embryos from *flt4^qmc316^* heterozygous incrosses before and after onset of SIVP flow. At 56 hpf, leading sprout number was not significantly different between wild-type, heterozygous and homozygous siblings (Fig. S14A-D). Similarly, at 72 hpf we observed no significant differences in SIVP area, plexus length, leading sprout length (Fig. S14E-I) or EC number (Fig. S14J-O) across genotypes. Thus, *flt4^qmc316^* mutants do not display detectable SIVP patterning defects under normal-flow conditions that could account for their increased sprout retention when flow is reduced.

**Fig. 7.**
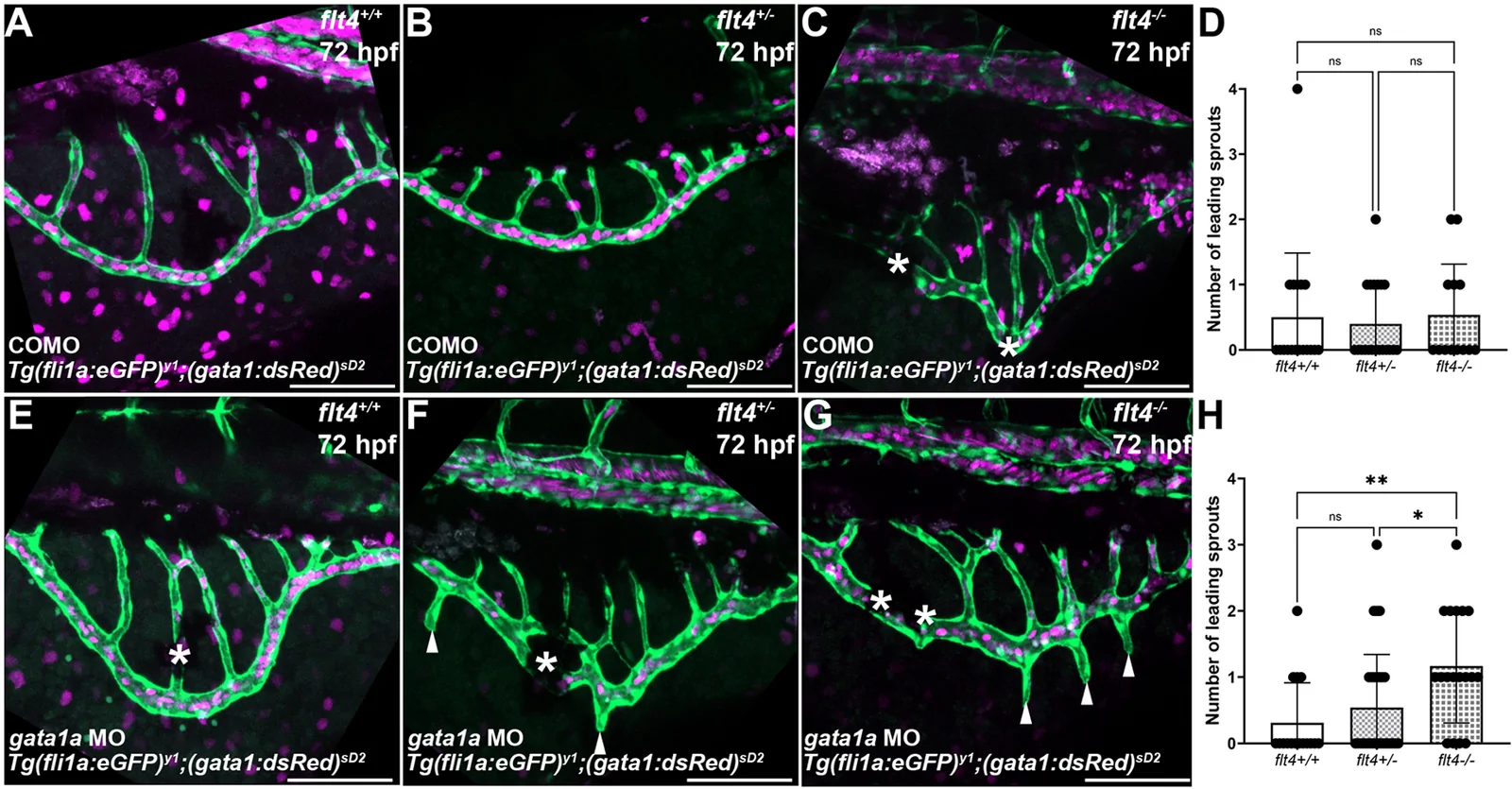
**Leading sprout regression under low blood flow conditions is dependent on *flt4*.** (A-D) The number of leading sprouts observed in wild type (A), *flt4* heterozygous mutants (B) or *flt4* homozygous mutants (C) under normal flow conditions, injected with morpholino, was not significantly different (D). (E-H) Reduced reduced-viscosity flow conditions, caused by *gata1* morpholino injection did not increase leading sprout numbers in wild type (E) or *flt4* heterozygous mutants (F), but leading sprouts in *flt4* homozygous mutants (G; white arrowheads) were significantly increased in number (H; arrowheads). **P≤0.01; *P≤0.05 (one-way ANOVA, with multiple comparisons). ns, not significant (*P*≥0.05). Control morphant *flt4^+/+^*, *n*=18; control morphant *flt4^+/−^*, *n*=20; control morphant *flt4^−/−^*, *n*=13; *gata1* morphant *flt4^+/+^*, *n*=16; *gata1* morphant *flt4^+/−^*, *n*=37; *gata1* morphant *flt4^−/−^*, *n*=18 (3 replicates each). Graphs show mean±s.d. Apparent gaps in the SIV in some maximum-intensity projections reflect pigment cells obscuring endothelial fluorescence and were not scored as vascular discontinuities. Scale bars: 100 μm. White asterisks indicate pigmentation. COMO, control morpholino.

To test the temporal requirement for Flt4 during leading sprout regression, we treated *gata1a* morphants with the Flt4 tyrosine kinase inhibitor MAZ51 from 56 to 72 hpf. We first identified a dose of MAZ51 that impaired lymphatic development during this window without altering SIVP flow velocity (Fig. S15A-E). Because MAZ51 can affect additional VEGFR-related pathways, we next assessed whether this treatment caused gross defects in cranial blood-vessel patterning. Cranial vascular density, network length and branchpoint number were not significantly altered in MAZ51-treated embryos compared with controls (Fig. S15F-J). Finally, neither MAZ51 treatment nor *gata1a* knockdown alone significantly increased leading sprout retention, whereas combined MAZ51 treatment and *gata1a* knockdown caused a significant increase in retained sprouts (Fig. S15K-O). Together with the *flt4^qmc316^* mutant analysis, these pharmacological data support a requirement for Flt4 activity during sprout regression under reduced-viscosity flow conditions.

We next tested whether acute partial inhibition of flow during the regression window affected sprout retention in *flt4^qmc316^* mutants. Treatment with 10 μM nifedipine from 56 to 72 hpf reduced blood flow velocity without abolishing circulation (Fig. S16A). Under these conditions, retained leading sprout number increased in nifedipine-treated *flt4^qmc316^* mutants compared with DMSO-treated controls (Fig. S16B-F). Wild-type nifedipine-treated embryos showed an intermediate phenotype and were not significantly different from either nifedipine-treated *flt4^qmc316^* mutant embryos or DMSO-treated controls. Similarly, treatment with 2× tricaine from 56 to 72 hpf significantly reduced blood flow velocity (Fig. S16G). Under these conditions, *flt4^qmc316^* mutants displayed increased retention of leading sprouts compared with control mutants, untreated control siblings, and control siblings treated with 2× tricaine (Fig. S16H-L). Together, these data show that acute flow reduction during the sprout regression window is sufficient to increase leading sprout retention. Finally, to test whether leading sprout regression requires canonical Vegfc signalling, we performed G0 CRISPR mutagenesis of *vegfc*. We titrated the *vegfc* dgRNPs to a level that generated mutations and impaired lymphatic formation without causing oedema that would compromise blood flow (Fig. S17A-F′). Leading sprout number was not significantly different between *vegfc* crispants and controls (Fig. S17G-I). These data indicate that partial *vegfc* disruption sufficient to impair lymphatic development does not phenocopy the reduced-flow *flt4* phenotype. Therefore, while *flt4* is dispensable for sprout regression under normal flow conditions, Flt4 activity becomes important when flow is reduced. Together, these findings show that blood flow drives SIVP remodelling by redirecting endothelial migration and identify Flt4 as a low-flow mediator of sprout regression (Fig. 8).

**Fig. 8.**
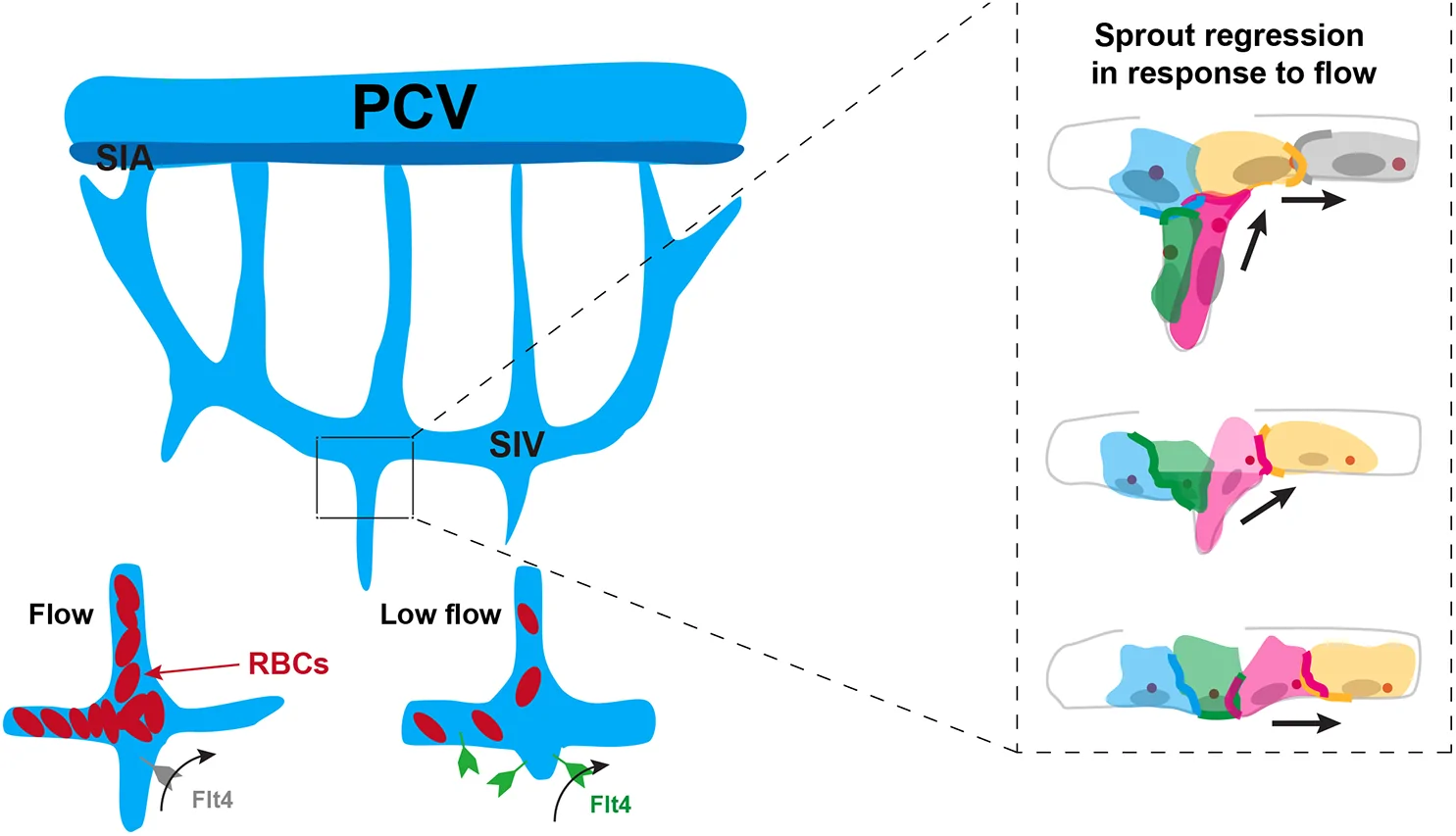
**Cellular mechanisms of flow-mediated sprout regression.** Schematic depicting cellular behaviours during SIVP sprout regression in the presence and absence of flow. Blood flow promotes dorsal EC migration and leads to sprout regression via Flt4 under low blood flow conditions. Illustrations highlighted with dashed lines depict coordination of EC migration during sprout regression in response to flow. SIV ECs (grey/yellow) at the luminal surface migrate laterally against flow and sprout ECs (green/pink) in the regressing sprout are pulled or use their neighbouring cells as migratory substrates via EC junctions (thickened lines), followed by EC rearrangements that lead to sprout regression. PCV, posterior cardinal vein; RBCs, red blood cells; SIV; sub-intestinal vein; SIA, supra-intestinal artery.

## DISCUSSION

Developmental remodelling of vascular networks has been described in different models and has mainly focused on vascular cross-branch pruning (Chen et al., 2012; Franco et al., 2015; Kochhan et al., 2013; Lobov et al., 2011; Phng et al., 2009). Pruning occurs in loop forming or cross-branch vessel segments, which ultimately leads to regression of the cross branch, leaving behind the two parallel vessels via a process involving EC migration and apoptosis (Chen et al., 2012; Zhang et al., 2018). Dynamic EC migration and rearrangement have also been described in sprouts; however, studies have mostly focused on angiogenic sprouting and anastomosis (Arima et al., 2011; Bentley et al., 2014; Jakobsson et al., 2010). Whether SIVP sprouts ultimately anastomose with each other or not, and how these sprouts are remodelled, remained unknown. Here, using the zebrafish SIVP we find most leading sprouts within the developing plexus are remodelled via EC migration-driven regression rather than anastomosis. Apoptosis during pruning has been described in murine retinal vessels when circulation is compromised (Franco et al., 2016, 2015; Hughes and Chan-Ling, 2000) and also in zebrafish cranial arteries (Kochhan et al., 2013; Zhang et al., 2018), but this has not been implicated during branch pruning within the SIVP (Lenard et al., 2015). Consistent with this, we tracked approximately 70% of SIVP ECs per plexus and, while we cannot exclude the possibility that EC apoptosis occurs in the most anterior or posterior SIVP, or in the SIA, we observed no apoptotic events following live imaging of over 100 leading sprouts. Therefore, our data suggest that EC migration represents the primary mechanism of sprout regression within the SIVP. Indeed, angiogenic regression is an efficient system that recycles ECs from transient sprouts, re-purposing them in other vascular structures. Thus, coordination of EC migration represents a more efficient use of resources than sculpting vascular networks by proliferation or apoptosis.

Haemodynamic force exerted by blood flow has been proposed to be crucial in selecting and triggering pruning and regression of particular vessels (Chen et al., 2012; Franco et al., 2015; Kochhan et al., 2013; Lenard et al., 2015; Lucitti et al., 2007). Pruning mostly occurs in small and bifurcated branches with relatively unstable, or low, blood flow in comparison to adjacent large vessels (Chen et al., 2012; Lenard et al., 2015). This observation suggests that pruning is triggered by local differences in flow patterns between branches, which in turn induce EC polarisation and direct EC migration against flow, from low-flow to high-flow vessels (Franco et al., 2015). Our data are consistent with this and upon initiation of SIVP sprout regression, ECs within non-perfused leading sprouts may respond to the relatively higher-flow environment of the SIV by undergoing directional rearrangement and dorsal migration into the SIV. In line with this, ventral EC migration persisted throughout the SIVP in *tnnt2a* morphants, indicating that a flow-independent mechanism controls this aspect of EC migration. However, formation of the stereotypical basket-like structure consisting of well-defined branches, SIVs and leading sprouts occurs even in the absence of flow. This suggests that SIVP formation is regulated by well-orchestrated molecular patterning cues that may interact with physical forces such as blood flow to refine plexus development. Indeed, loss of guidance molecules such as Plexin D1 disrupt SIVP patterning (Goi and Childs, 2016) and Plexin D1 has recently been identified as a shear stress mechanosensor in ECs (Mehta et al., 2020); however, it is unclear whether Plexin D1 modulates EC response to flow during SIVP remodelling.

Remodelling of blood vessels from primitive structures into mature networks via EC response to flow temporally separates migratory sprouting from branch pruning during angiogenesis (Lenard et al., 2015). In the SIVP, new sprouts are rarely produced after the onset of flow and most existing sprouts remodel via regression. While the entire plexus expands ventrally during this period, the tip cells within leading sprouts do not simply undergo initial ventral migration followed by dorsal migration during regression; rather, these ECs display transient and stochastic dorsal migration steps while undergoing collective, flow-independent, ventral migration. This suggests that sprouting and regression are interlinked. An intriguing question is at which point such steps lead to irreversible sprout regression. One possibility is that at the onset of flow the posterior membranes of the tip cells are connected with adjacent SIV ECs and have limited exposure to blood flow within the SIV. It is possible that when blood flow creates sufficiently high shear forces on SIV cells, it drives their migration against flow, which may exert a pulling force on the tip cells, promoting their dorsal migration and inducing sprout regression. The SIV cells and the tip cells in contact with flow could act as bridging ECs to sense differences in the local flow environment, similar to branch pruning (Franco et al., 2015). That the combined plexus and sprout length in *tnnt2a* morphants was not significantly different to controls indicates that, in the absence of blood flow, ECs within sprouts can migrate the same distance as in controls. Reduced plexus length in the absence of blood flow is therefore a consequence of increased contribution of ECs from the branches and SIVs to leading sprouts. In the presence of blood flow, ECs within sprouts migrate against flow and contribute to the SIV and branches, thereby supporting plexus expansion. This is consistent with reports that ECs incorporate in developing arterial networks in the retina and coronary vasculature via coordinated EC migration (Chang et al., 2017; Pitulescu et al., 2017). Our data therefore suggest that blood flow promotes regression of leading sprouts within the SIVP as a mechanism to support expansion of the developing plexus. Moreover, sprouting and regression share similar cellular behaviours but in a ‘reverse mode’.

Yolk sac vascular remodelling is dependent upon erythroblast circulation (Lucitti et al., 2007), and uneven erythrocyte distribution in bifurcations may enhance local shear stress differences between vessels undergoing pruning and their neighbouring vessels (Zhou et al., 2021). Thus, blood viscosity is strongly associated with vessel regression. How ECs sense low levels of flow remains unclear, but several possible molecular mechanisms have been proposed. The level of VEGFR3 might maintain a threshold of shear stress sensed by ECs (Baeyens et al., 2015). Interaction of the transforming growth factor β (TGFβ) receptor activin receptor-like kinase Acvrl1 (also known as Alk1) with a component of the TGFβ receptor complex, Endoglin, has been reported to enhance EC sensitivity of flow sensing (Baeyens et al., 2016). In addition, non-canonical Wnt signalling may control vessel regression by modulating the threshold for flow-induced EC polarisation (Franco et al., 2016). We find that, although plexus length was reduced in erythrocyte-depleted embryos, even substantial reductions in blood viscosity were compatible with sprout regression. We find that sprout regression in the SIVP is dependent upon *flt4* under reduced flow conditions, but Flt4 function is dispensable for sprout regression under normal blood flow. This indicates that additional mechanisms could be involved to regulate sprout regression under normal flow conditions. Consistent with this, *flt4* mutants display normal SIVP formation (Hogan et al., 2009a,b and this study), suggesting different functions of Flt4 under different flow conditions in zebrafish. The established role of VEGFR2 during EC mechanosensation (Coon et al., 2015; Tzima et al., 2005) suggests that Kdr and Kdrl are promising candidates to mediate sprout regression under normal flow; however, their requirement during SIVP formation precluded their assessment in this study. The redundant function of Flt4 during sprout regression imparts robustness on the system and facilitates proper vessel remodelling under dysregulated flow conditions. It is important to note that, although SIVP leading sprouts are not perfused with erythrocytes before or during their regression, a low level of shear provided by skimmed plasma could promote dorsal migration of tip cells to elicit regression (Zhou et al., 2021).

Our analysis of *vegfc* further suggests that this reduced-flow requirement for Flt4 is not simply explained by canonical Vegfc-dependent signalling. v*egfc* crispants displayed impaired lymphatic development, consistent with effective disruption of *vegfc* function, but did not show defective SIVP leading sprout regression. Similarly, *vegfc* expression was not obviously altered in *gata1a* morphants. However, because residual *vegfc* activity in crispants cannot be excluded, and because our *flt4* allele has not been definitively established as a molecular null, we cannot distinguish whether Flt4 acts through low-level ligand-dependent signalling, ligand-independent mechanosensing, or interaction with additional flow-responsive pathways. Future work using domain-specific *flt4* alleles, including mutations that selectively disrupt Vegfc binding or mechanosensory function, will be required to resolve how Flt4 couples reduced haemodynamic input to the cytoskeletal and migratory changes that drive sprout regression.

Together, our data demonstrate that blood flow drives remodelling of a venous plexus by coordinating EC migration and sprout regression and identify Flt4 as a context-specific mediator of this process under reduced-flow conditions. These insights highlight the dynamic interplay between mechanical forces and molecular signalling in shaping vascular architecture and underscore the importance of flow-responsive pathways in venous remodelling.

## MATERIALS AND METHODS

### Zebrafish strains

Maintenance of zebrafish and experimental procedures involving zebrafish were carried out according to UK national guidelines and under UK Home Office licences. The following zebrafish lines were employed in this study: *nacre^w2^* (Lister et al., 1999), *Tg(fli1a:*EGFP*)^y1^* (Lawson and Weinstein, 2002)*, Tg(gata1a:*DsRed*)^sD2^* (Traver et al., 2003), *Tg(kdrl:HRAS-*mCherry*-CAAX)^s916^* (Chi et al., 2008), *Tg(fli1a:LifeAct-mClover)^sh467^*, *Tg(fli1a:AC-TagRFP)^sh511^* (Savage et al., 2019), *Tg(kdrl:EGFP)^s843^* (Jin et al., 2005), *Tg(fli1a:nls-*mCherry*)^sh550^* (this study), *Tg(fli1a:nls-*EGFP*)^sh549^* (this study), *flt1*^bns29^ (Matsuoka et al., 2016), *flt4^qmc316^* (this study). All animal research was carried out in accordance with The Animals (Scientific Procedures) Act 1986 (ASPA).

### Generation of transgenic lines

*Tg(fli1a:nls-*EGFP*)^sh549^* and *Tg(fli1a:nls-*mCherry*)^sh550^* were generated using the Tol2 Kit (Kwan et al., 2007) by standard methods as previously described (Savage et al., 2019) using the following components: p5E-fli1aep (Villefranc et al., 2007) pME-mCherry or pME-EGFP, p3E-SV40pA and pDestTol2-pA2. Embryos were injected at the one-cell stage with 25 ng/μl *Tol2* mRNA and corresponding plasmid DNA.

### Microinjection of morpholinos and mRNA

Microinjections were performed on single-cell embryos with 1 nl injection volume. Embryos were injected with 0.8 ng morpholinos (Table S1) including control, *tnnt2a* (Sehnert et al., 2002), *flt4* (Hogan et al., 2009b) and *gata1a* (Galloway et al., 2005), or 200 pg mRNA including *mTurquoise2* and *vegfaa_165_* (Lawson et al., 2002).

### G0 CRISPR mutants

Target sequences were ordered as crRNAs (Merck) and were either designed for this study (*vegfc*) or used previously published sequences (*tnnt2a*) (Wu et al., 2018) (Table S1) and co-injected alongside tracrRNA (Merck) in equimolar ratios and EnGen^®^Spy Cas9 NLS protein (NEB) into one-cell zebrafish embryos. Universal non-targeting gRNA was designed as described by Wierson et al. (2019).

### Chemical inhibitor treatments

Embryos were treated from 56 hpf until 72 hpf with 10 μM or 20 μM nifedipine to reduce or ablate blood flow, 200 μM MAZ51 (Merck) to inhibit Flt4 function, 2× tricaine (MS-222; 328 mg/l) to reduce blood flow velocity, or equivalent volumes of DMSO as vehicle controls.

### RNA *in situ* hybridisation

Alkaline phosphatase whole-mount *in situ* hybridisation experiments were performed using standard methods as described previously (Wilkinson et al., 2012) using probes against *mflt1* (Krueger et al., 2011), *sflt1* (Krueger et al., 2011), *flt4* (Thompson et al., 1998) and *kdrl* (Fouquet et al., 1997).

### Live imaging of zebrafish embryos and larvae

Zebrafish were anaesthetised using Tricaine (MS-222, Sigma-Aldrich) and embedded in 1% low-melting point agarose, which was either held in place in a glass capillary and imaged using a light sheet Z.1 microscope (Zeiss), or in a glass-bottom Petri dish imaged on either a Cell Discoverer 7 (Zeiss) or LSM880 microscope (Zeiss). Imaging chambers contained E3 and tricaine (164 mg/l) to ensure embryos and larvae remained anaesthetised throughout imaging. Imaging was performed at 28.5°C. Images were acquired using ZEN software (Zeiss). Zebrafish were imaged at 5-min intervals to detect blood flow, and 10-min intervals to track EC nuclei. To image individual erythrocytes in *gata1a* morphants, time lapses were taken at 50 frames per second for 30 s with 1 μm *z*-stacks. The number of erythrocytes that passed a defined region of the SIV within a 30 s period were manually counted.

### Quantification of SIVP morphology

Image quantification and analysis was performed using Fiji (Schindelin et al., 2012). Parameters employed to quantify SIVP morphology, including area, length, vascular loops, sprout number and EC number, were recorded in a region of five somite widths. The length of the SIVP was measured as the vertical distance from the SIA to the most-ventral part of the SIV. To ensure only endothelial nuclei within the SIVP were quantified, dual channel fluorescence was employed to highlight EC cytosol and nuclei. To quantify average SIV diameter, the SIV was measured at three equidistant points positioned anteroposteriorly and the mean value was calculated.

### EC rearrangement and cell trajectory analysis

*Tg(fli1a:nls-*mCherry*)^SH550^* heterozygous embryos were used for cell-tracking studies. Time-lapse images were pre-processed using Linear Stack Alignment with SIFT (Lowe, 2004) in Fiji and the movements of EC nuclei were tracked using TrackMate (Tinevez et al., 2017). Misconnected tracks were manually corrected. ECs were classified and colour-coded according to their initial position at the start of tracking, 56 hpf, as tip cells (magenta), SIVP branch cells (orange) or SIV cells (blue). At the end of observation (72 hpf), final cell positions were scored to determine whether ECs remained within the same compartment or had moved between compartments. For example, an EC initially located in the SIV that was located in a branch at 72 hpf was recorded as a single rearrangement event. Initial and final EC positions, and EC rearrangement events, were manually recorded from each time lapse.

Migration-step displacements, velocities and angles were first calculated in MATLAB from exported TrackMate coordinates. For each tracked EC nucleus, a migration step was defined as the displacement between two consecutive tracked positions. For a cell moving from position 1, with coordinates *x*1 and *y*1, to position 2, with coordinates *x*2 and *y*2, step displacement was calculated as 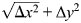. Track distance was calculated as the sum of all step displacements for an individual cell. Step velocity was calculated as step displacement divided by the time interval between consecutive frames, and mean velocity for a given time window was calculated as the average step velocity within that window. Migration angles were calculated using the four-quadrant inverse tangent (*atan2*) function (Δ*y*, Δ*x*), where Δ*x*=*x*2−*x*1 and Δ*y*=*y*2−*y*1.

Rose plots and directional bias metrics were then generated from the exported migration step coordinates using custom Python scripts. In Python, migration angles were recalculated using the NumPy arctan2 function to assign each step to angular bins for rose-plot visualisation. In Fig. 5 and Fig. S4, rose plots contain 16 angular bins spanning 0°-360°, with each bin covering 22.5°. Each petal represents the number of single-frame migration steps falling within that angular bin. The radial scale therefore reflects frequency of directional migration steps, not the cumulative distance travelled in that direction.

Reverse migration and nearest neighbour distances were analysed using Python (v3.10). For reverse migration analysis, cell tracks were converted into displacement steps between consecutive TrackMate spots. Each step was represented as a vector and projected onto a user-defined anatomical migration axis. Unless otherwise specified, the ventral migration axis was used as the reference axis, reflecting the dominant direction of SIVP expansion over the yolk. The reverse fraction was defined as the proportion of steps with a negative axial projection, corresponding to movement opposing the defined migration axis (projection D<0). Thus, in analyses using the ventral axis, reverse fraction represents the proportion of migration steps opposing ventral movement rather than migration against blood flow. Nearest neighbour distances were calculated for each spot in each frame by measuring the Euclidean distances between all spot centroids. The nearest neighbour distance for each spot was calculated as the minimum non-zero distance to any other spot within the same frame. Because SIVP development involves large-scale ventral morphogenetic expansion, migration-step directionality was interpreted as a measure of directional bias superimposed on overall plexus growth, rather than as a direct measure of net dorsal displacement into the SIV.

### EC nucleus eccentricity analysis

Time-lapse images of *Tg(fli1a:AC-TagRFP)^SH511^; (fli1a:nls-EGFP)^SH549^* were captured between 56 and 75 hpf, during leading sprout regression. To quantify nuclear eccentricity, individual EC nuclei were tracked over time, and nuclear eccentricity was measured in each frame. For each nucleus, eccentricity values were averaged over the regression period and compared between regressing sprout ECs and neighbouring SIVP ECs using a custom Python script available at https://github.com/fishyvessels/.

### Generation of *flt4^qmc316^* allele

gRNAs targeting *flt4* (ENSDARG00000104453) were ordered from IDT DNA Technologies and targeted the sequence 5′-CCCAGGGTATAGAGAAATCAGGC-3′ in exon 3. crRNAs were annealed with tracrRNA (IDT) as previously described (Kroll et al., 2021). Annealed sgRNAs were injected into one-cell stage zebrafish embryos alongside EnGen^®^ Spy Cas9 NLS (NEB, M0646) in a non-complexed solution. F1 progeny of G0 embryos were genotyped using high-resolution melt analysis and PCR using the primers 5′-AGTGGAGTTTACTGATCGCCA-3′ and 5′-CCGTGGTGCCATCAATAACA-3′ to confirm the presence of the *flt4^qmc316^* allele. *flt4^qmc316^* consists of a complex insertion and deletion resulting in a net 132 bp insertion, which is predicted to shift frame after 83 residues and truncate following 11 subsequent incorrect residues within the first extracellular Ig-like domain and earlier than previously published *flt4* alleles including a reported null allele within exon 4 (Shin et al., 2016).

### Blood flow analysis

Embryos were mounted laterally in 35 mm glass-bottomed Petri dishes and a 20 s brightfield movie was captured at 30 fps. Red blood cell flow was visualised using image subtraction using a custom ImageJ macro (available at https://github.com/fishyvessels/), followed by use of the Unsharp Mask plugin to enhance moving pixel visibility. An 1800 μm section of the dorsal aorta was cropped out for analysis. The ImageJ Trackmate (Ershov et al., 2022) plugin was used to link pixels between frames to calculate velocity. Foci with a radius of over 1 μm were filtered for analysis. Kalman filter was then selected and 20 μm set as the initial search radii and a maximum of 1 frame gap allowed, with subsequent filters to select for cells with a forward progression value of >0.8 and a track displacement of >10 μm. Values were exported as a .csv file for subsequent analysis, using a custom Python script available at https://github.com/fishyvessels/.

### Cranial vascular patterning analysis

Cranial vascular patterning was quantified from confocal *z*-stacks of *Tg(kdrl:EGFP)^s843^* embryos treated with DMSO or MAZ51 from 56 to 72 hpf. Maximum-intensity projections of the cranial vasculature were generated in Fiji and analysed using VISTA-Z (Rodriguez-Pastrana et al., 2026). A consistent region of interest encompassing the cranial vascular network was applied across embryos. Analysis was performed with the operator unaware of treatment condition.

### Statistical analyses

Statistical analyses and graph plots were performed using GraphPad Prism. All statistical analysis is described in figure legends, including paired/unpaired *t*-test, ordinary one-way ANOVA and two-way ANOVA. All error bars display the mean and standard deviation in the figures. *P*-values, unless exact value is listed, are listed as follows: ns (not significant), *P<0.05, **P≤0.01, ***P≤0.001, ****P≤0.0001. Statistical analysis was performed with the operator unaware of groups. In morpholino experiments, the researcher was unaware of the group to which each embryo belonged. For mutant experiments, group assignment information was available as required for genotyping. Tests for normality were performed prior to assumptions of normal distribution and non-parametric tests were performed in the absence of normal distribution. Appropriate sample size was determined by post-hoc power calculations.

## Supplementary Material



10.1242/develop.205424_sup1 Supplementary information
