# Supplementary material for: Blood flow coordinates collective endothelial cell migration during vascular plexus formation and promotes angiogenic sprout regression via flt4
Source: Development. 2026 Oct 6;153(19):dev205424. doi: 10.1242/dev.205424 (PMC13723401; doi:10.1242/dev.205424)
Supplement: Supplementary file 1 [file develop-153-205424-s1.pdf]

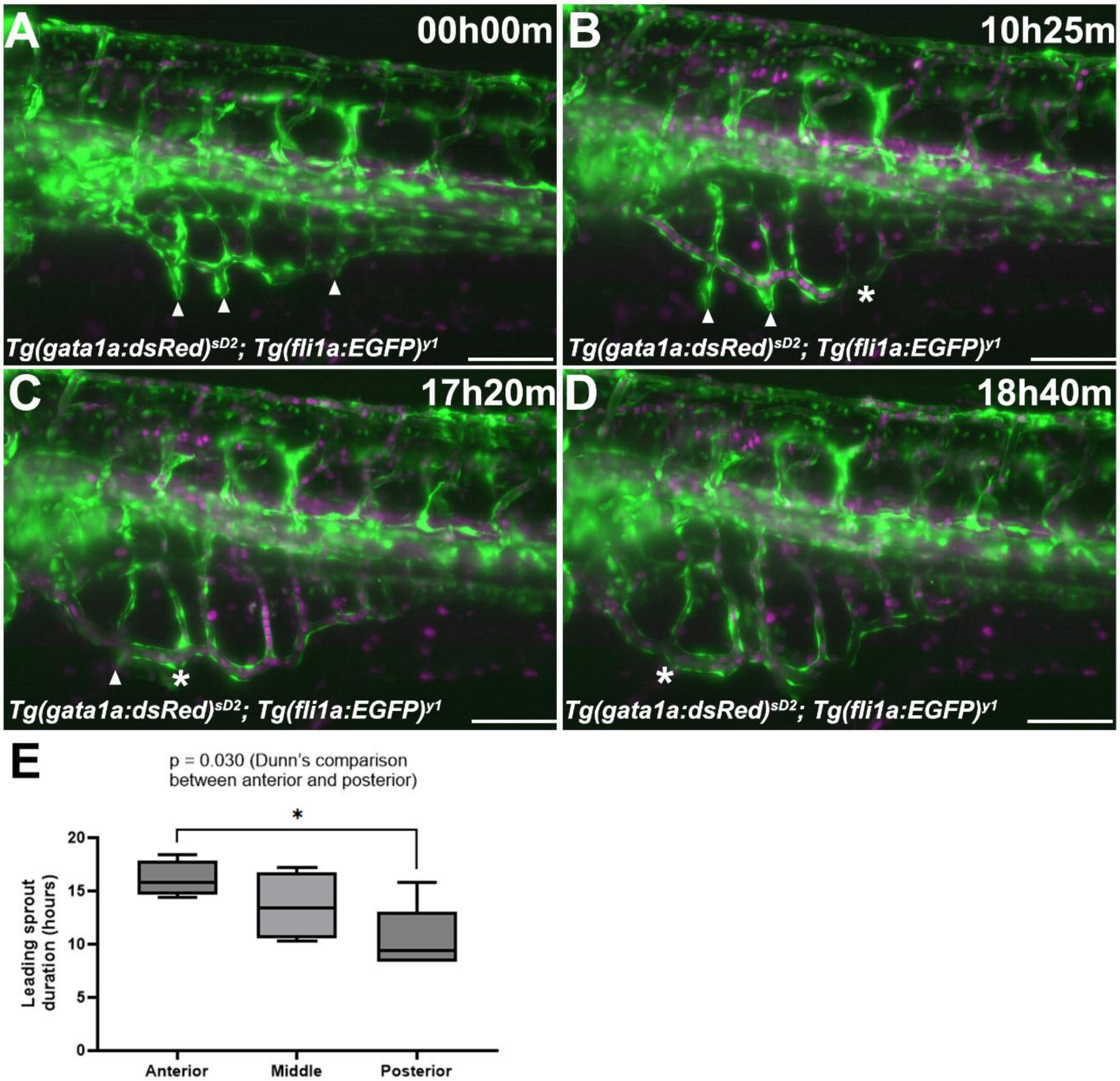

Fig. S1. Leading sprout regression shows a posterior bias during SIVP remodelling.

A-D) Still images from representative time-lapse showing regression of leading sprouts at different anterior-posterior positions within the SIVP. Posterior sprouts regress earlier than more anterior sprouts. White arrowheads indicate leading sprouts; white asterisks indicate regressed sprouts. **E)** Quantification of relative sprout duration according to anterior-posterior position. Posterior sprouts persist for significantly less time than anterior sprouts. n=5 embryos. Data are presented as standard box-and-whisker plots showing the median (center line), upper and lower quartiles (box limits), and minimum and maximum values (whiskers). Kruskal-Wallis test with Dunn's correction; $p<0.05$

Development • Supplementary information

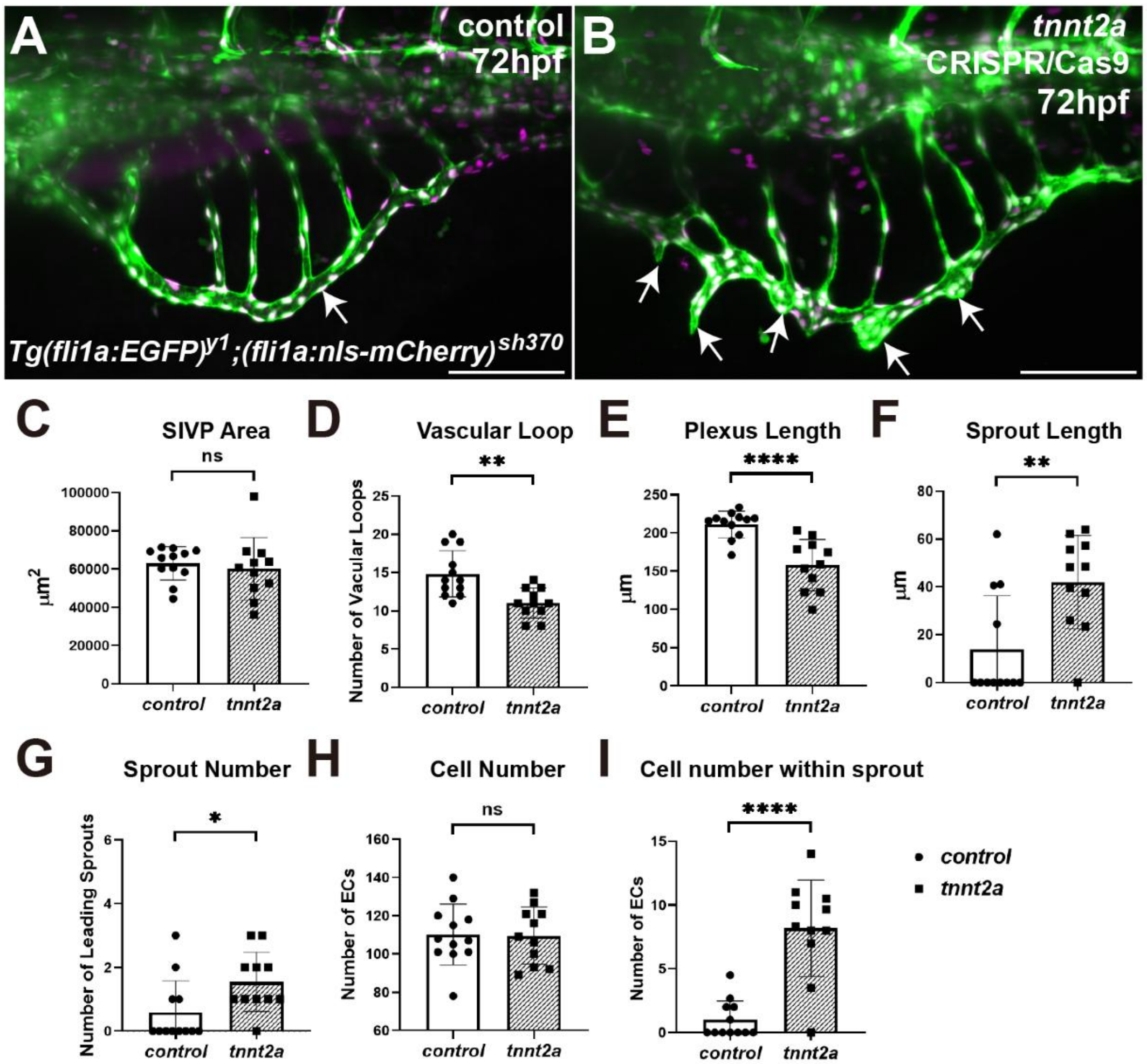

Fig. S2. SIV development in *tnnt2a* F0 mosaic CRISPR mutants phenocopies *tnnt2a* morphants

A, **B**) Embryos were injected with control gRNA and tracr RNA or *tnnt2a* gRNAs and tracr gRNA alongside nCas9 protein. Comparison of SIVP morphology at 72hpf in the presence (control, **A**) or absence of blood flow (*tnnt2a* mosaic mutant, **B**). **C**) No significant difference in plexus area was observed between control or *tnnt2a* mosaic mutants. **D, E**) *tnnt2a* mosaic mutants displayed fewer vascular loops than controls (**D**) and length of SIVP basket was reduced (**E**). **F**, **G**) *tnnt2a* mosaic mutant embryos displayed increased sprout length and number in comparison to controls. **H, I**) The total number of ECs in the SIV was not altered in *tnnt2a* mosaic mutants in comparison to controls, however, leading sprouts contained more ECs in *tnnt2a* mosaic mutants (**I**). Control, n=12; *tnnt2a*, n=11; Unpaired t-test, ****$p \leq 0.0001$, ns, $p \geq 0.05$. 3 replicates. Graphs show mean and standard deviation. Scale bars 100µm.

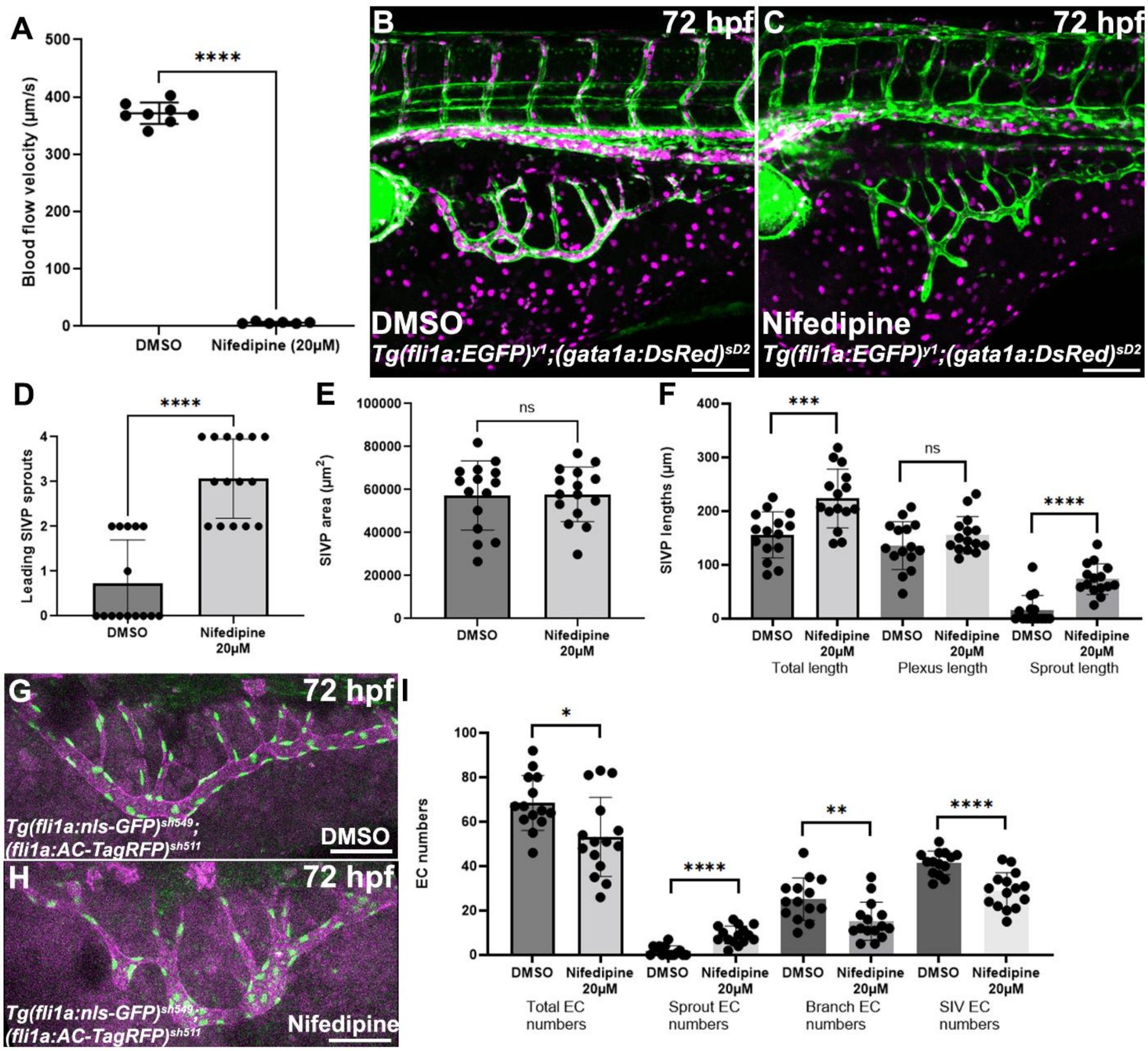

Fig. S3. Acute flow cessation phenocopies loss of *tnnt2a* function during SIVP remodelling. A) Treatment with 20µM Nifedipine from 56-72hpf abolishes blood flow. Unpaired t-test, ****p<0.0001. Two independent repeats; DMSO, n=8; nifedipine, n=6. **B-C)** Nifedipine-treated embryos lack detectable circulating erythrocytes in the SIVP compared with DMSO-treated controls. **D)** Nifedipine treatment significantly increases leading sprout number. Unpaired *t*-test; ****p<0.0001. Three independent repeats; DMSO, n=15; nifedipine, n=15 **E)** SIVP area is not significantly altered by nifedipine treatment. Unpaired t-test; ns, not significant. Three independent repeats; DMSO, n=15; nifedipine n=15 **F)** Leading sprout length and total SIVP length (plexus length + leading sprout length) are significantly increased following nifedipine treatment, whereas plexus length is not significantly altered. Unpaired *t*-tests; ***p<0.001; ****p<0.0001. Three independent repeats; DMSO, n=15; nifedipine n=15. **G, H)** Endothelial nuclei are less uniformly distributed within the SIVP following nifedipine treatment. **I)** Nifedipine treatment reduces total endothelial cell number and increases the number of endothelial cells retained in leading sprouts relative to branches or the SIV. Unpaired *t*-tests, *p<0.05; ** p<0.01; ****p<0.0001. Three independent repeats, DMSO, n=15; nifedipine, n=14. Scale bars: 100µm.

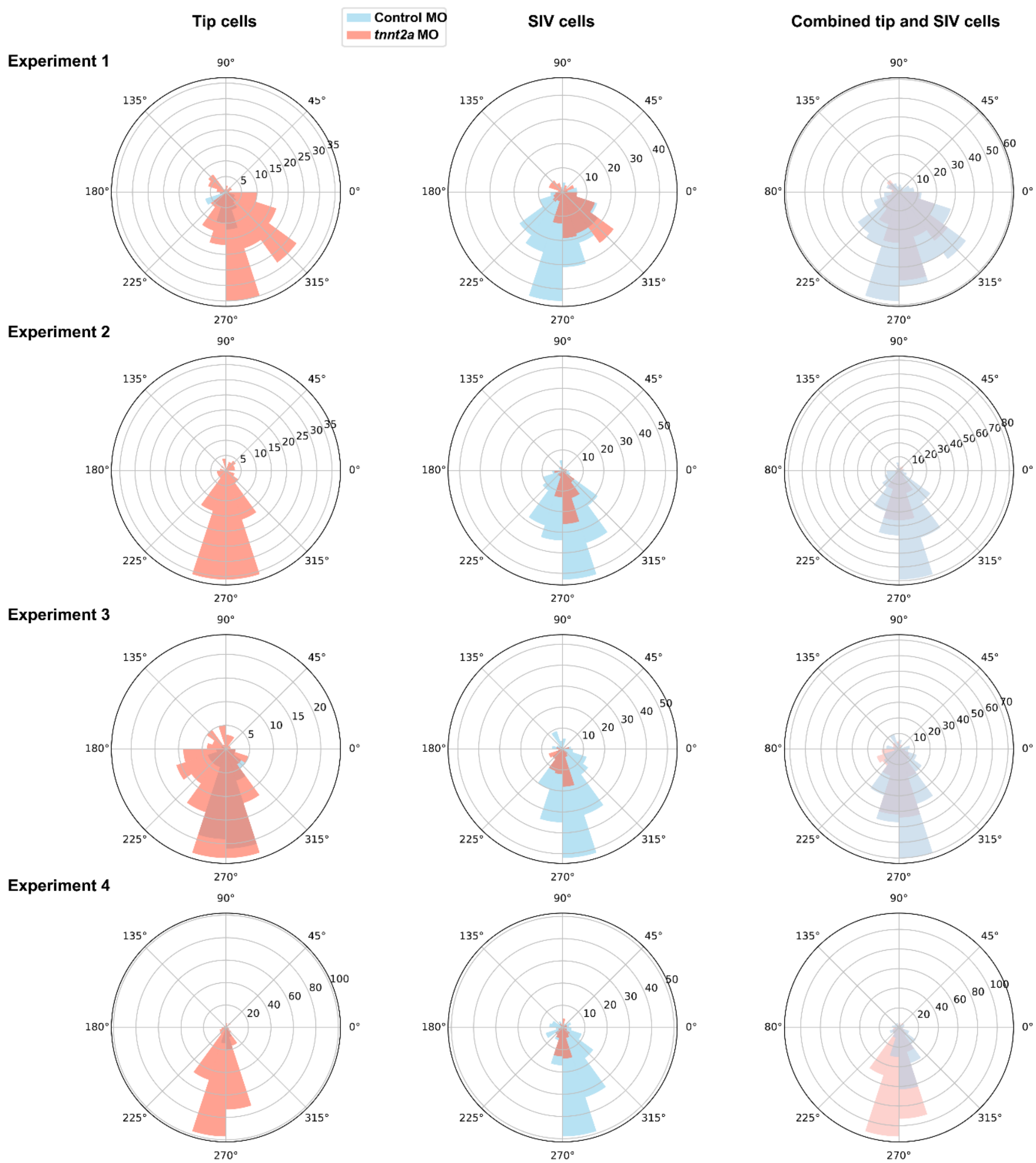

Fig. S4. *tnnt2a* knockdown alters the directionality of SIVP endothelial cell migration. Rose plots showing endothelial cell migration-step direction in paired control and *tnnt2a* morphant embryos. Each row represents an independent experiment, labelled by experiment number. Migration steps are shown for tip cells, SIV ECs and all tracked SIVP ECs. Control embryos are shown in blue and *tnnt2a* morphants in red. Angles indicate migration direction, with 270° corresponding to ventral movement. *tnnt2a* morphant tip cells display increased ventral migration compared with control tip cells, whereas control SIV cells display stronger ventral/posterior migration than *tnnt2a* morphant SIV cells.

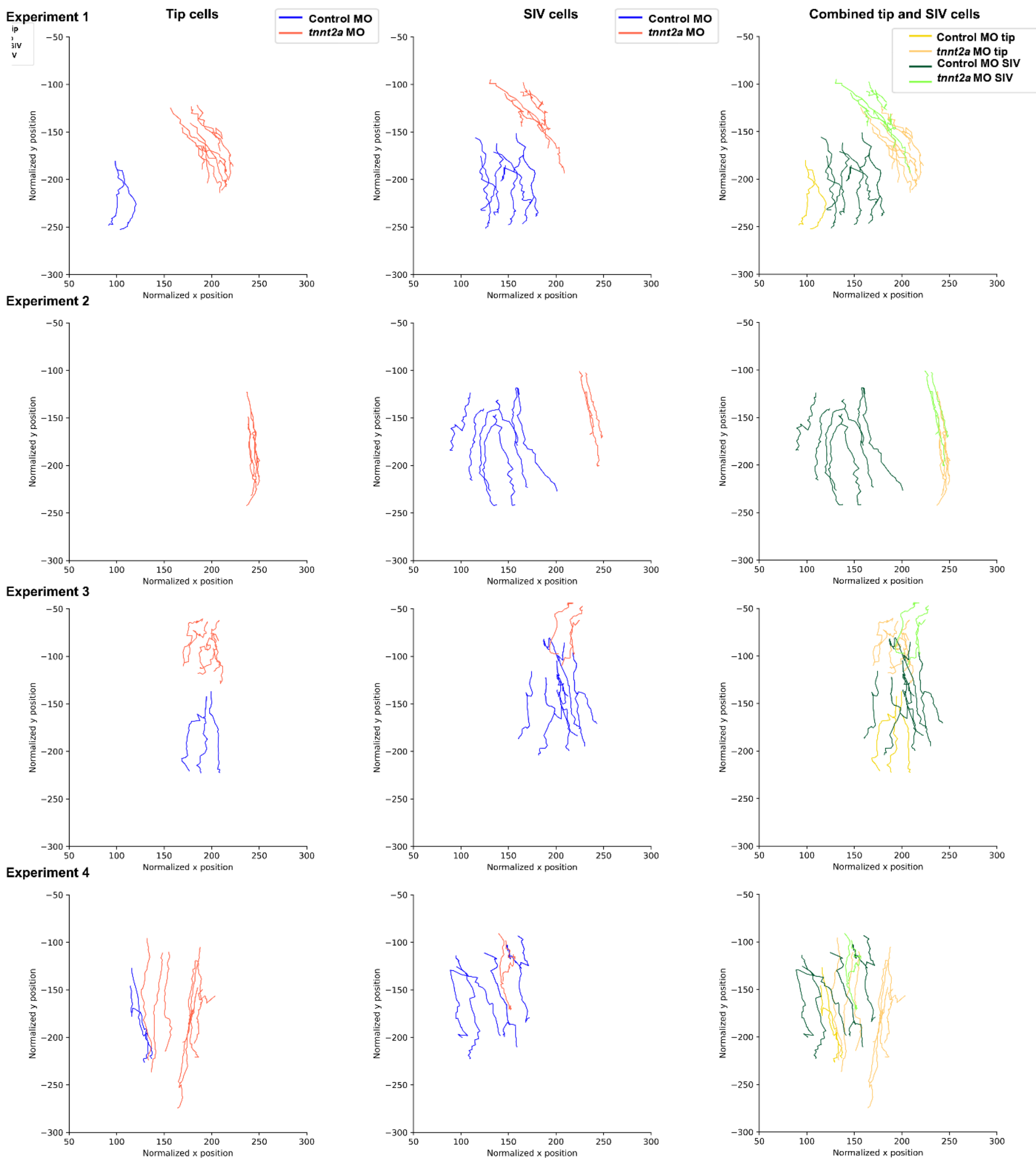

Fig. S5. ***tnnt2a*** **knockdown alters endothelial cell trajectories during SIVP remodelling.** Tracks showing the movement of individual endothelial cells in paired control and *tnnt2a* morphant embryos. Tracks are shown for tip cells, SIV ECs and all tracked SIVP ECs. Control morphant embryos are shown in blue and *tnnt2a* morphants in red. *tnnt2a* morphant tip cells remain more closely clustered during migration and are retained in greater numbers, consistent with impaired resolution of ventral sprout migration and reduced dorsal rearrangement during sprout regression.

Development • Supplementary information

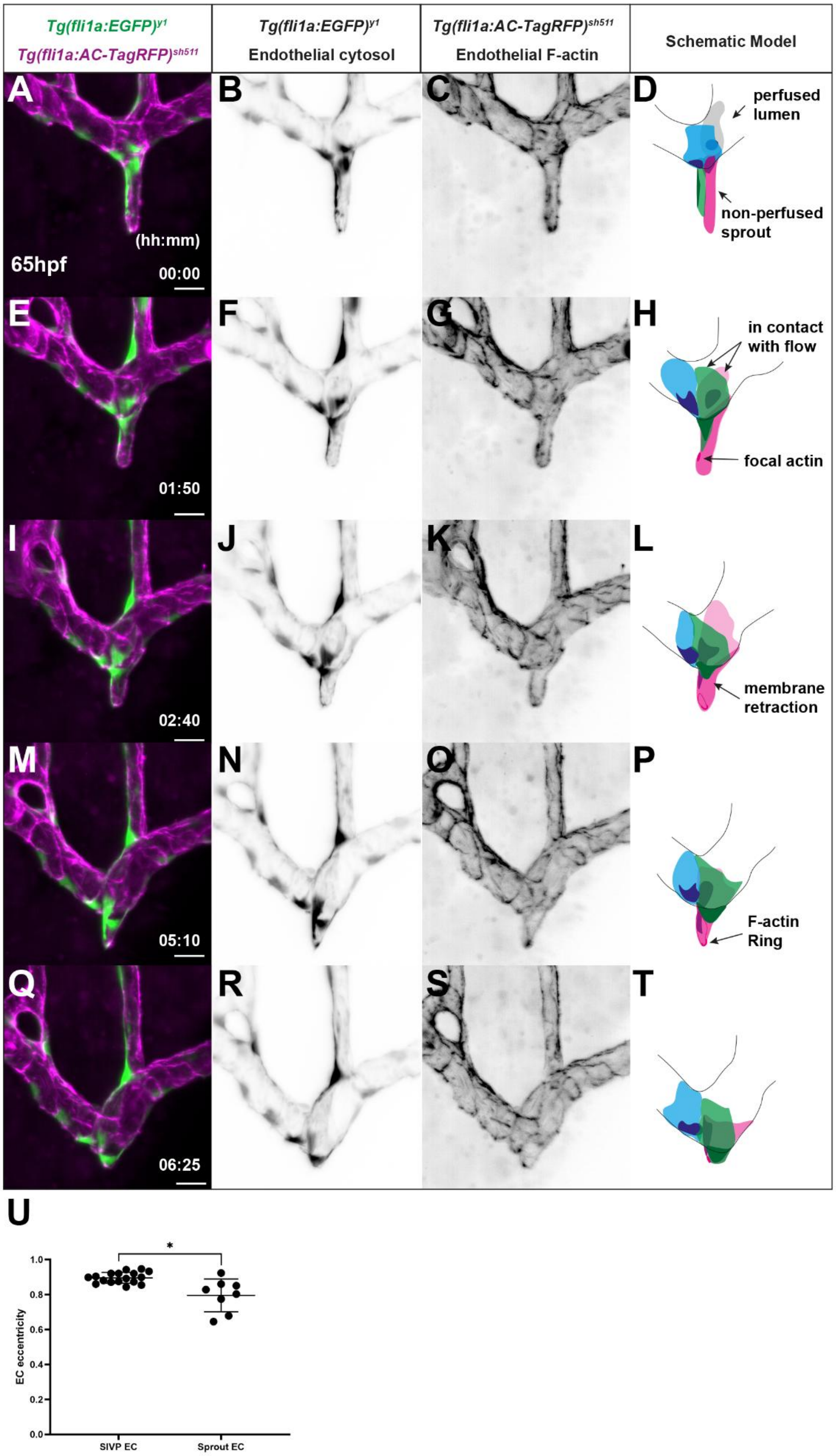

Fig. S6. Cellular mechanisms of sprout regression

A-E) Representative images of leading sprout regression taken from 6.5h time-lapse from 65hpf in *Tg(fli1a:EGFP)^{y1};(fli1a:AC-TagRFP)^{sh511}* embryo with normal blood flow (Supplementary Movie 7). GFP (EC cytosol and nucleus) and RFP (EC actin) channels are shown in addition to merged image. Nuclei were identified in *Tg(fli1a:EGFP)^{y1}*;*Tg(fli1a:AC-TagRFP)^{sh511}* embryos by combining the nuclear visibility of the *fli1a:eGFP* channel with nuclear exclusion from the AC-TagRFP signal. Schematic model is drawn to illustrate the dynamic process of EC arrangement. **A-D**) Leading sprout consisting of a pair of EC tip cells. The dorsal most EC membrane is in contact with blood flow within SIV lumen. **E-H**) During regression, one of the pair of tip cells (green) retracts most of its cell body within the SIV and positions its nucleus at the edge of the main vessel. The neighbouring tip cell (pink) retracts its membrane into the SIV while the distal region of the tip cell remains within the sprout. **I-L**) One of the tip cells (pink) continues to retract its distal membrane while the adjacent tip cell (green) holds its position and maintains vessel integrity. **M-P**) F-actin positive focus in the distal tip cell (pink) membrane grows into a ring which closes when the tip cell completes sprout regression. **Q-T**) Both tip cells complete regression and become part of the SIV. Scale bars 25μm. **U)** EC eccentricity is significantly higher in SIV ECs than in sprout ECs. Mann-Whitney U-test, *$p<0.05$, n=5 embryos.

Development • Supplementary information

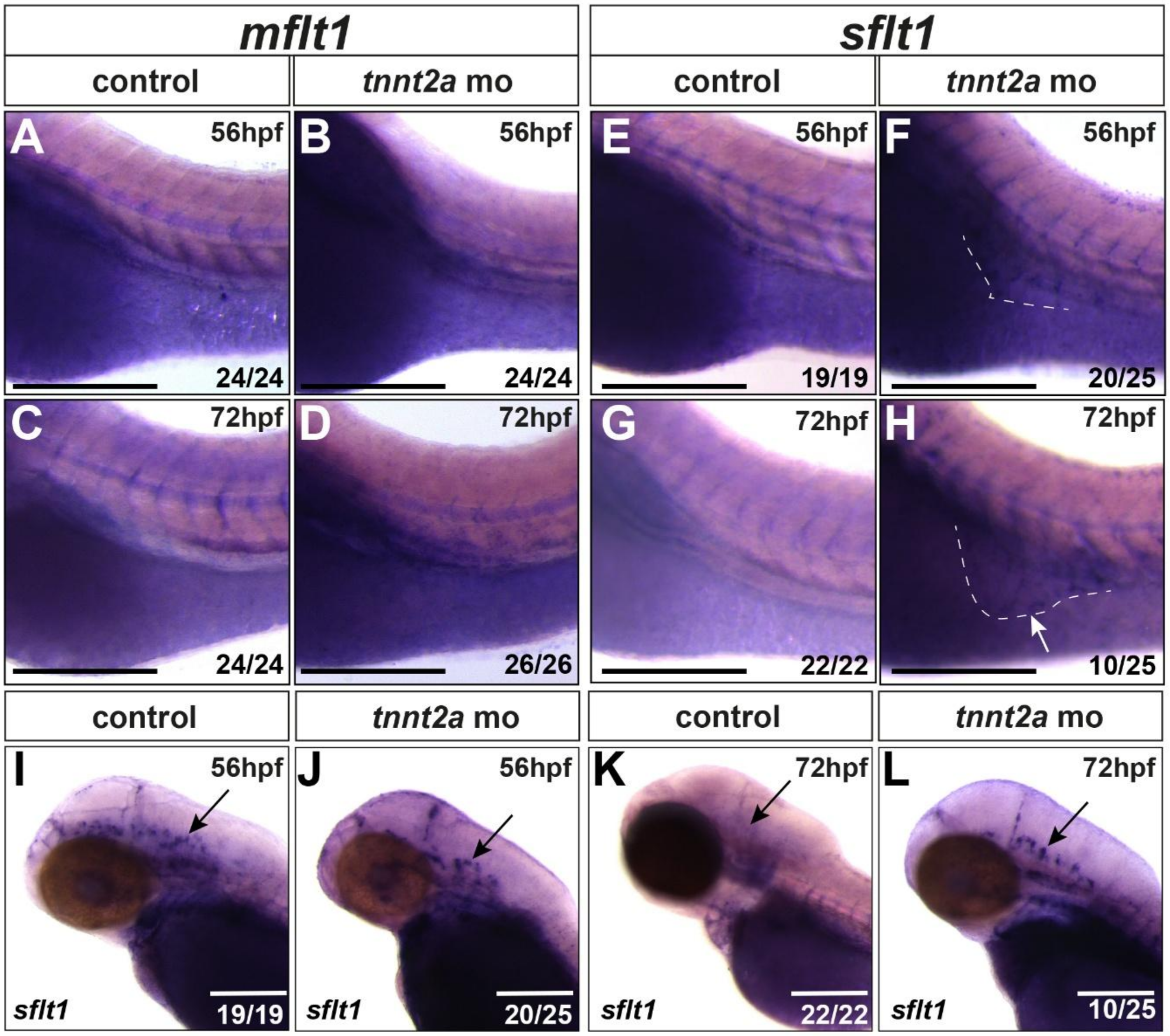

Fig. S7. *sflt1* expression is increased in the SIVP in the absence of blood flow
A-**D**) Expression of *mfl1* was not detected in the presence or absence of blood flow within the SIVP during the period of leading sprout regression. **E-H**) *sflt1* was not expressed in the SIVP in the presence of blood flow at the onset of leading sprout regression, but by 72hpf at the completion of regression, *slft1* was present in the SIVP only in the absence of blood flow (**H**, arrow). **I**, **J**) *sflt1* was expressed within central arteries at 56hpf in both the presence (**I**, arrow) and absence (**J**, arrow) of blood flow. **K**, **L**) slft1 expression was absent from central arteries by 72hpf (**K**, arrow), but persisted in embryos without flow (**L**, arrow). Scale bars: 100µm.

Development • Supplementary information

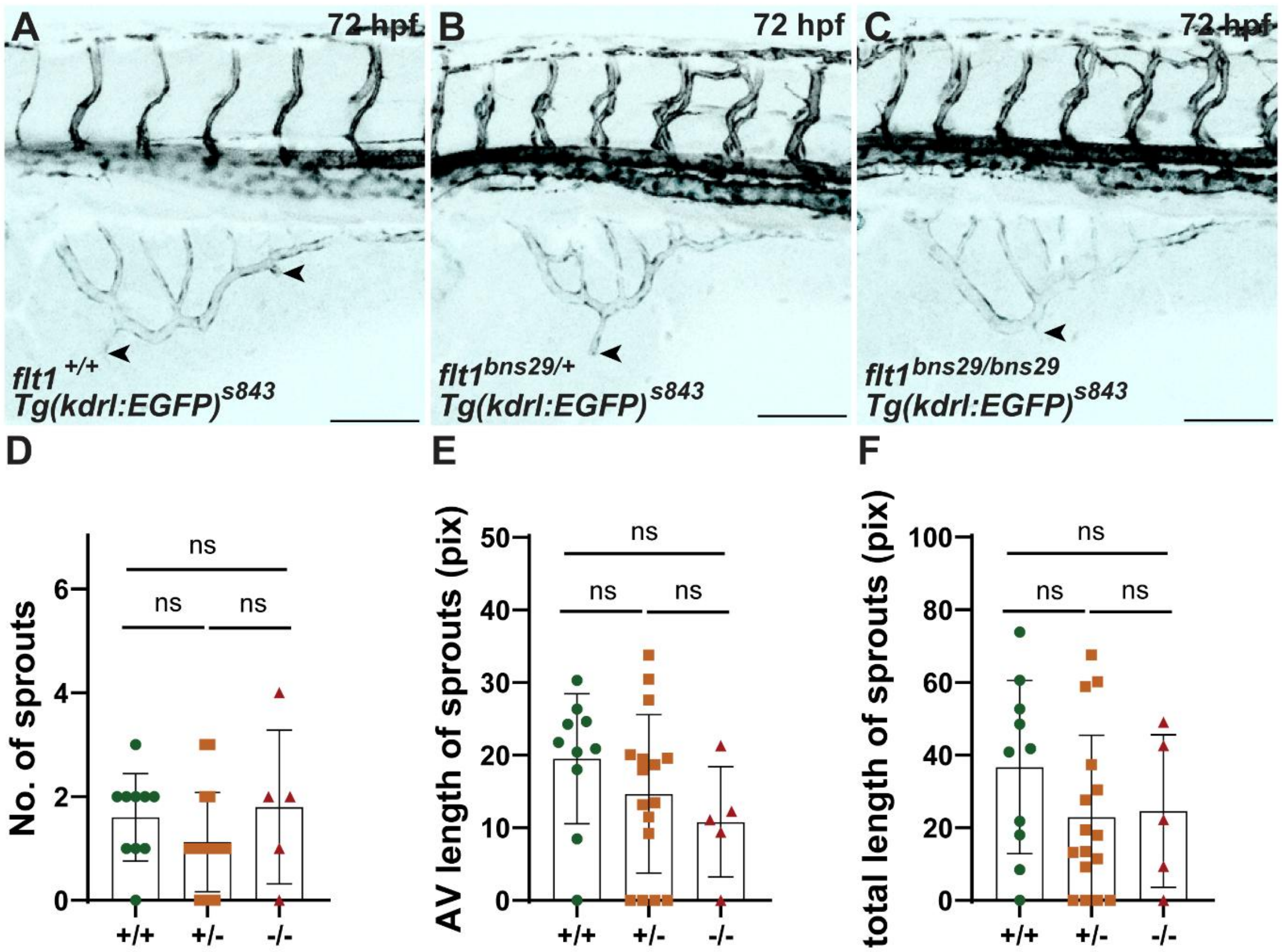

Fig. S8. Leading sprout regression occurs normally in the SIVP of *flt1* mutants

A-F) No significant differences were observed in the frequency (**D**), mean (**E**) or total length of sprouts measured in pixels (pix) (**F**) in WT, *flt1* heterozygotes or *flt1* mutant embryos at 72hpf. Unpaired t-test ns, $p \geq 0.05$. 3 replicates. Graphs show mean and standard deviation. Scale bars: 100µm.

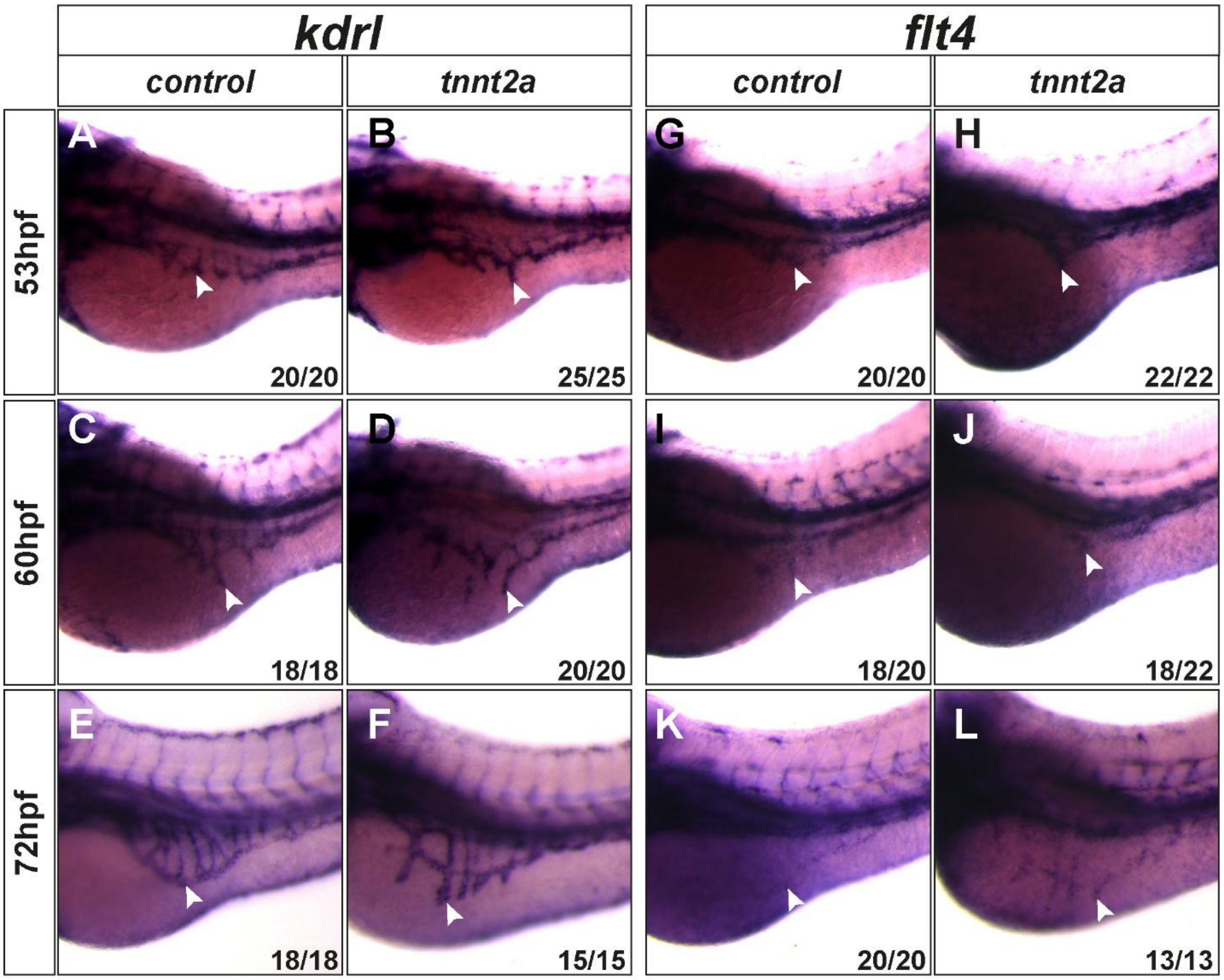

Fig. S9. Expression of *vegfr4/kdrl* and *vegfr3/flt4* are retained in leading sprouts in *tnnt2a* morphants.

Both *vegfr4*/*kdrl* and vegfr3/*flt4* were expressed in ECs of the SIVP before onset of flow at 53hpf, during sprout regression at 60hpf, and 72hpf in the presence or absence of flow. The expression of *kdrl* and *flt4* were retained in leading sprouts (arrowheads) which had failed to regress in the absence of flow by 72hpf. Scale bars: 100μm.

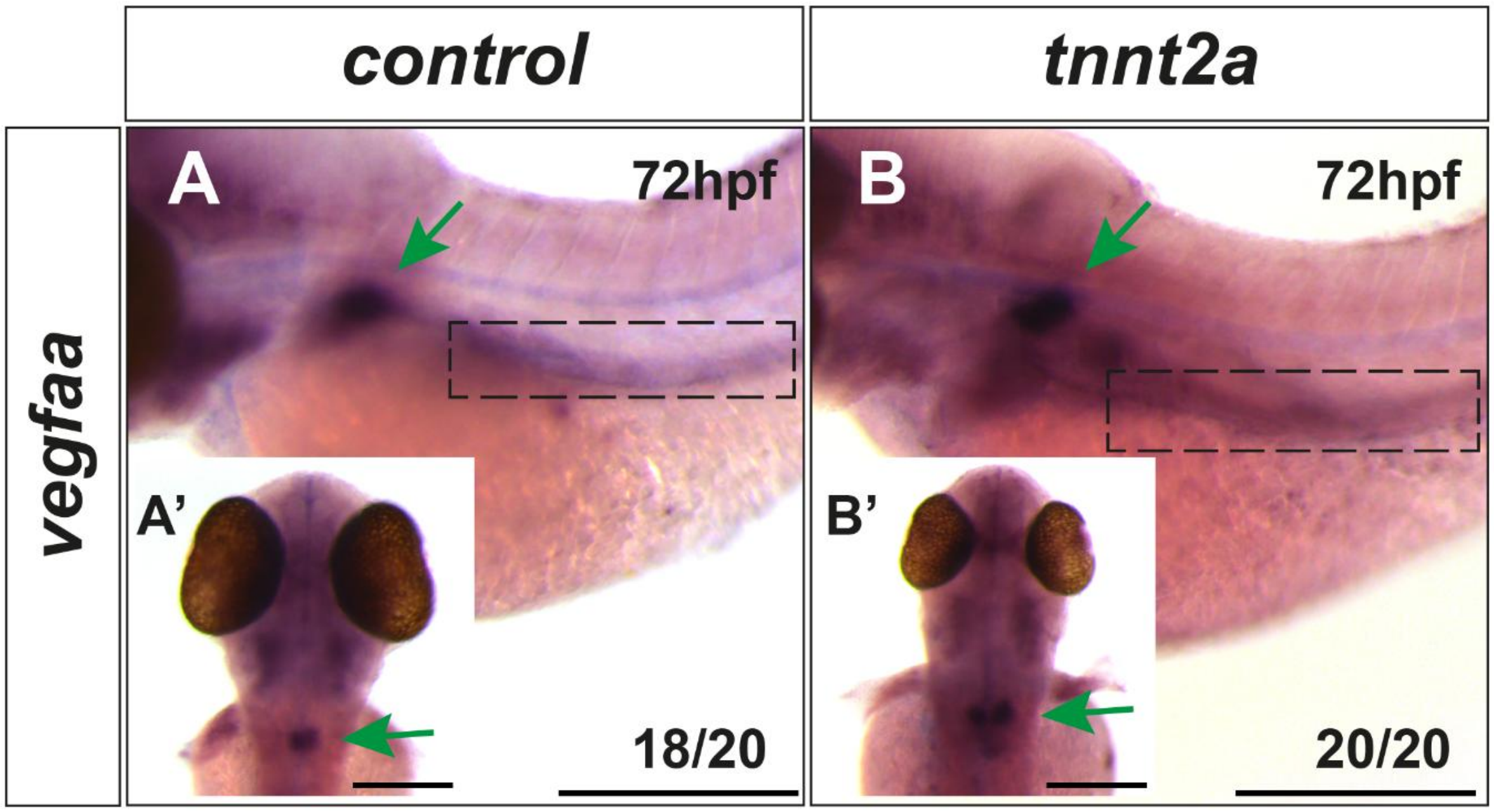

Fig. S10. Expression of *vegfaa* is not altered by the absence of flow
Expression of *vegfaa* is detected in the glomerulus of the developing kidney (green arrows) and regions near the SIA (dotted rectangles) and does not differ between *control* and *tnnt2a* morphants. The dorsal view of the embryos is shown at the bottom left (A'-B'). Scale bars: 100µm.

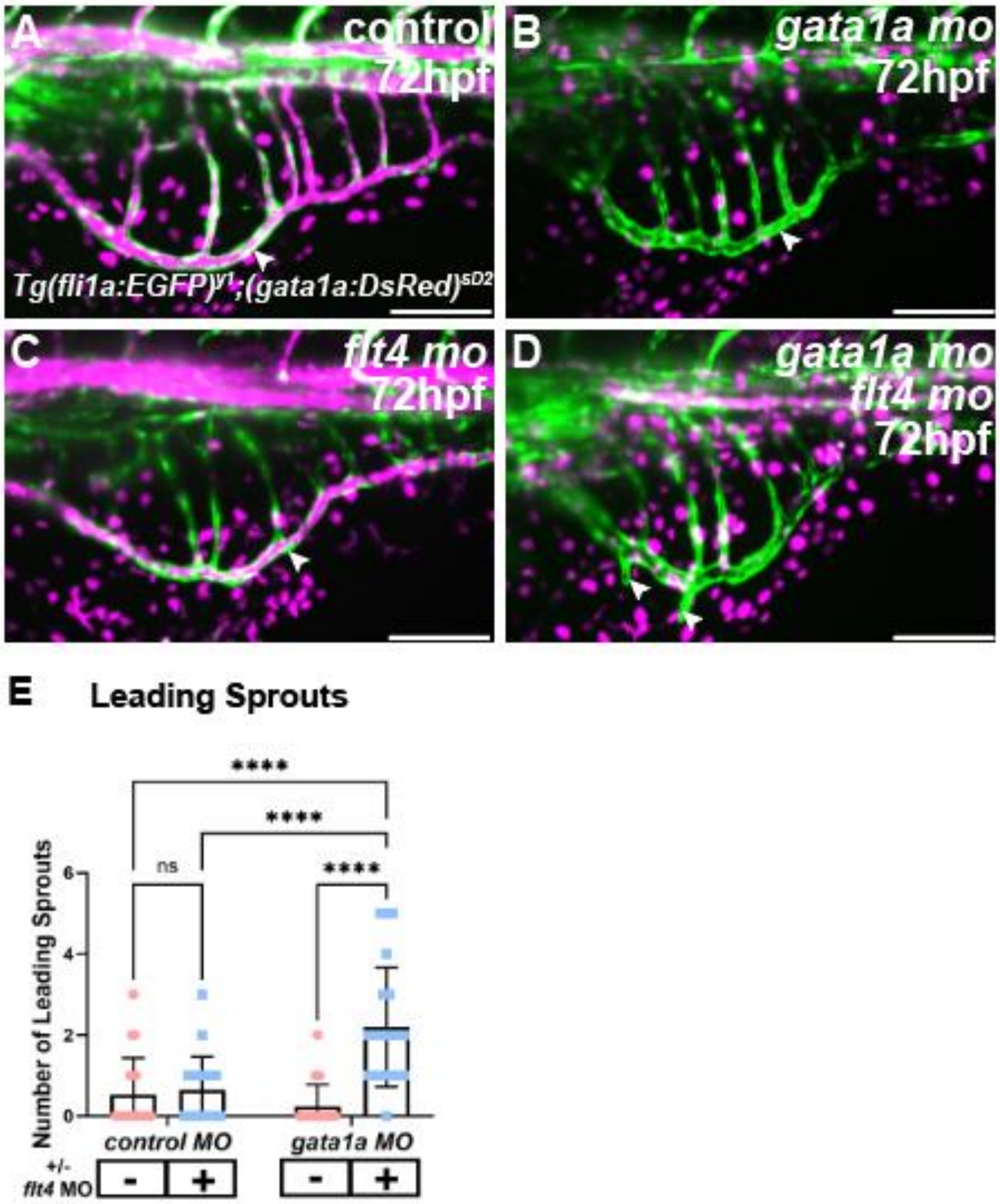

Fig. S11. Leading sprout regression under low blood flow conditions is dependent on *flt4*
In comparison to controls (**A**), *gata1a* morphants displayed reduced circulating erythrocytes within the SIVP without inhibiting leading sprout regression (**B**, arrowheads). Leading sprout regression was unaltered by *flt4* knockdown under normal flow conditions (**C**) but *flt4* knockdown inhibited sprout regression under low flow conditions (**D**, arrowheads). The frequency of leading sprouts was significantly increased in *flt4*/*gata1a* double morphants compared to either *flt4*, *gata1a* or control single morphants (**E**). Two-way ANOVA, **** $p \leqslant 0.0001$; *** $p \leqslant 0.001$; ** $p \leqslant 0.01$; ns $p \geq 0.05$. (**F**). Two-way ANOVA, **** $p \leqslant 0.0001$; *** $p \leqslant 0.001$; ** $p \leqslant 0.01$; ns $p \geq 0.05$; interaction F (1, 77) = 17.05, P<0.0001. Control morphants n=19; *flt4* morphants n=19; *gata1a* morphants n=21; *flt4; gata1a* morphants n=21, 3 replicates. Graphs show mean and standard deviation. Scale bars: 100µm.

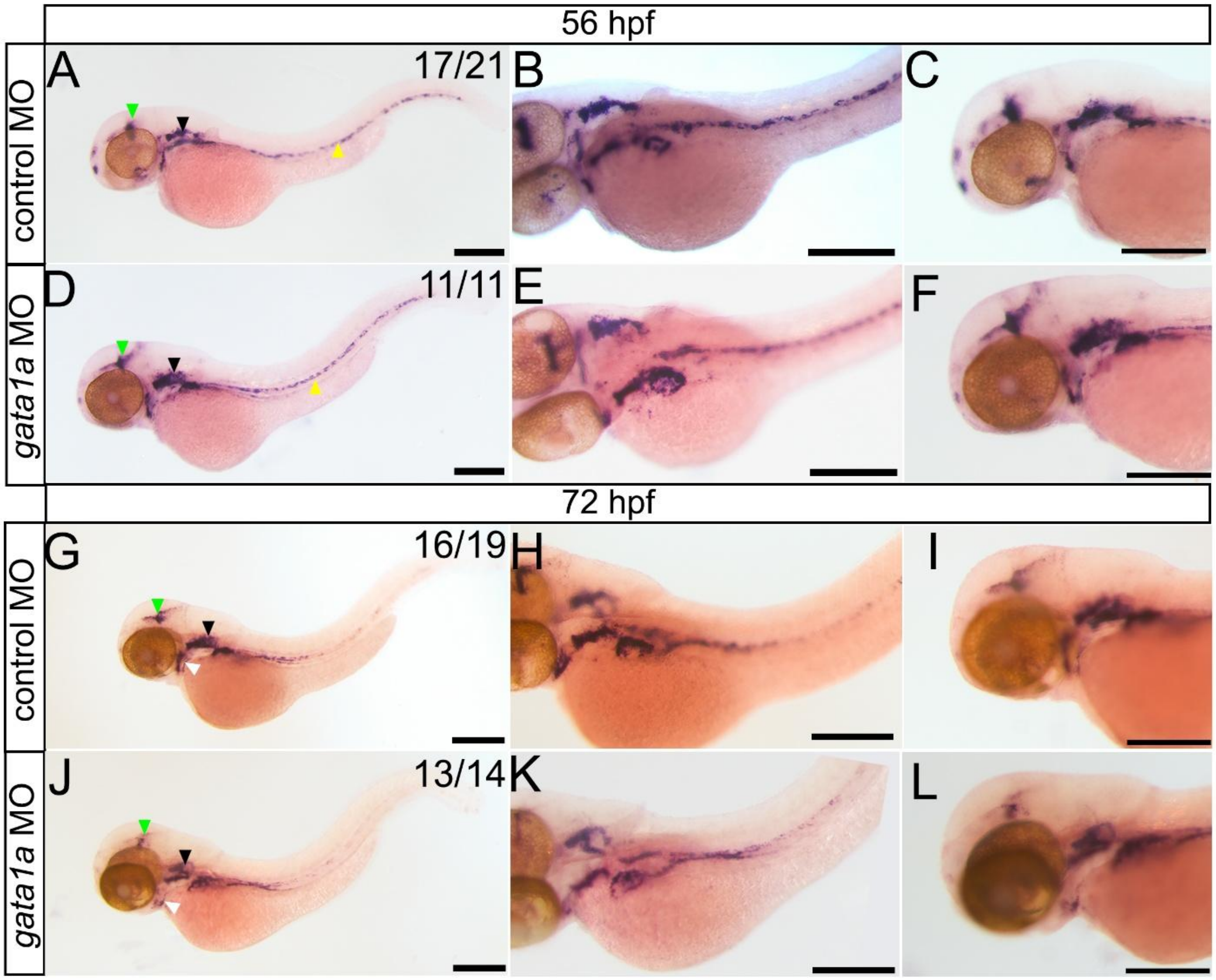

Fig. S12. *gata1a* knockdown does not alter the gross pattern of *vegfc* expression.
A-C) *vegfc* expression in control morphants at 56 hpf. Expression is detected in the dorsal aorta (yellow arrowhead), brain (green arrowhead), and pharyngeal endoderm (black arrowhead), with no obvious expression near the developing SIVP. **D-F)** *gata1a* morphants display *vegfc* expression comparable with controls at 56 hpf. **G-I)** *vegfc* expression in control morphants at 72 hpf. Expression is detected in the brain (green arrowhead), pharyngeal endoderm (black arrowhead) and pharyngeal arches (white arrowhead), with signal observed dorsal to the developing SIVP. **J-L)** *gata1a* morphants display *vegfc* expression comparable to controls at 72 hpf. Numbers indicate embryos showing the representative expression pattern. Scale bars 100μm.

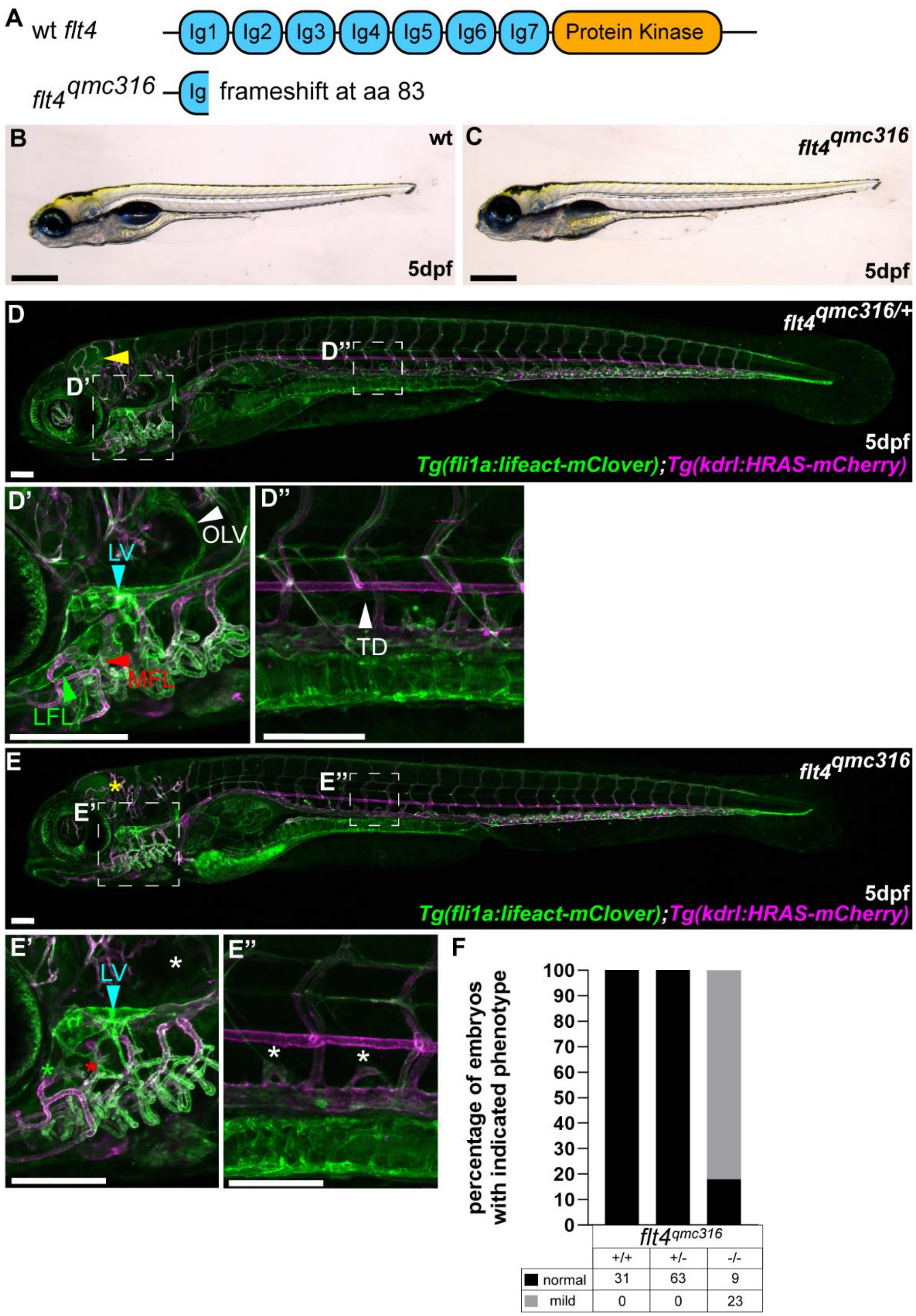

Fig. S13. *flt4^{qmc316}* mutants display impaired lymphatic development.

The *flt4^{qmc316}* mutant allele is predicted to prematurely truncate in the first immunoglobulin domain of Flt4, due to a frameshift at amino acid 83 (**A**) *flt4^{qmc316}* mutants display no embryonic oedema by 5dpf compared to wild type siblings (**B-C**). *flt4$^{qmc316/+}$* heterozygotes display stereotypical formation of embryonic lymphatic vasculature (**D**), including meningeal lymphatic endothelial cells (**D** yellow arrowhead), otolithic facial lymphatic vessel (**D'** white arrowhead, OLV), medial facial lymphatic vessel (**D'** red arrowhead, MFL), lateral facial lymphatic vessel (**D'** green arrowhead,

Development • Supplementary information

LFL), lymphatic valve (**D'** blue arrowhead, LV) and formation of the thoracic duct (**D''** white arrowhead). Conversely, *flt4*^{qmc316} mutants display impaired lymphatic vasculature formation (**E**), including loss of facial lymphatic vasculature, including a reduction in meningeal lymphatic endothelial cells (**E** yellow asterisk), loss of the otolithic facial lymphatic vessel (**E'** white asterisk), a loss of medial facial lymphatic vessel (**E'** red asterisk) and a loss of the lateral facial lymphatic vessel (**E'** green asterisk), along with failure to form the thoracic duct (**E''** asterisks). *flt4*^{qmc316} mutants retain the lymphatic valve (E', blue arrowhead). Compared to wild type and heterozygous siblings (100%), 90% of *flt4*^{qmc316} mutants were identifiable at 5 dpf by a slight thickening of the membrane behind the eye, suggesting mild oedema (**F**). Scale bars in (B, C) 500µm, scale bars in (D-E'') 100µm. Abbreviations: LV = lymphatic valve; LFL = lateral facial lymphatic vessel; MFL = medial facial lymphatic vessel.

Development • Supplementary information

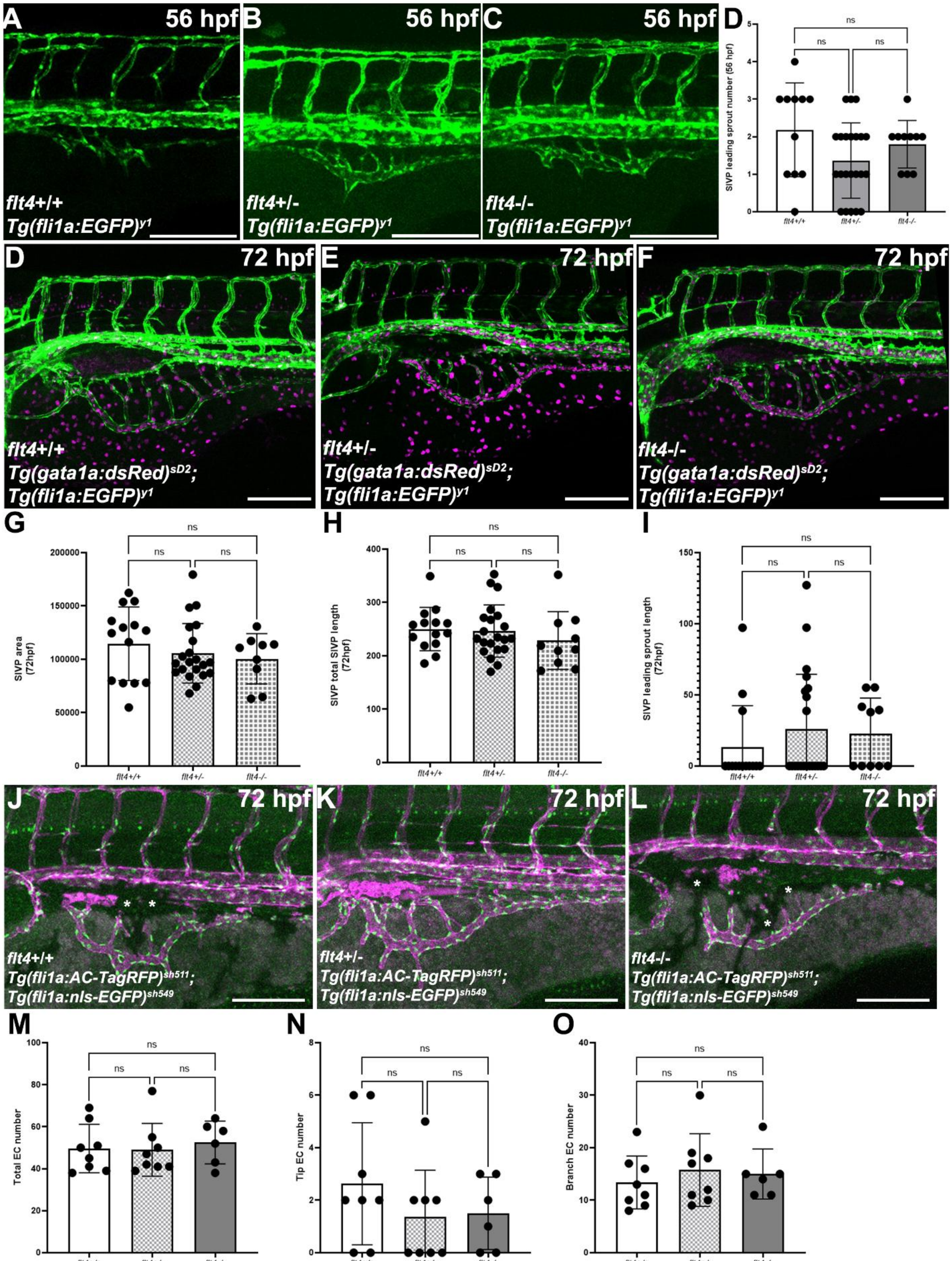

Fig. S14. ***fl4***^{qmc316} **mutants show normal SIVP development under unperturbed flow conditions**

Development • Supplementary information

A-C) *flt4^{qmc316}* mutants display comparable early SIVP development compared to heterozygous and WT siblings. **D)** No significant difference is observed between sibling genotypes when comparing leading sprout numbers at 56 hpf. 3 repeats, *flt4+/+* n=11, *flt4+/-* n=22, flt*4-/-* n=10. **D-F)** *flt4^{qmc316}* mutants display comparable late SIVP development compared to heterozygous and WT siblings. **G)** SIVP area is not significantly different between sibling genotypes. 3 repeats, *flt4+/+* n=14, *flt4+/-* n=21, flt*4-/-* n=12. **H)** SIVP total length is not significantly different between sibling genotypes. 3 repeats, *flt4+/+* n=14, *flt4+/-* n=21, flt*4-/-* n=12. **I)** SIVP sprout length is not significantly different between sibling genotypes. 3 repeats, *flt4+/+* n=14, *flt4+/-* n=21, flt*4-/-* n=12. **J-L)** Representative images showing *flt4^{qmc316}* mutants display comparable SIVP EC numbers and distribution compared to heterozygous and WT siblings. White asterisks indicate overlying pigment. **M-O)** There is no significant difference in total EC numbers, tip EC numbers and branch EC numbers between sibling genotypes. 2 repeats, *flt4+/+* n14, *flt4+/-* n18, flt*4-/-* n=11. All analysis One-way ANOVA with multiple comparisons. All Scale bars 100µm.

Development • Supplementary information

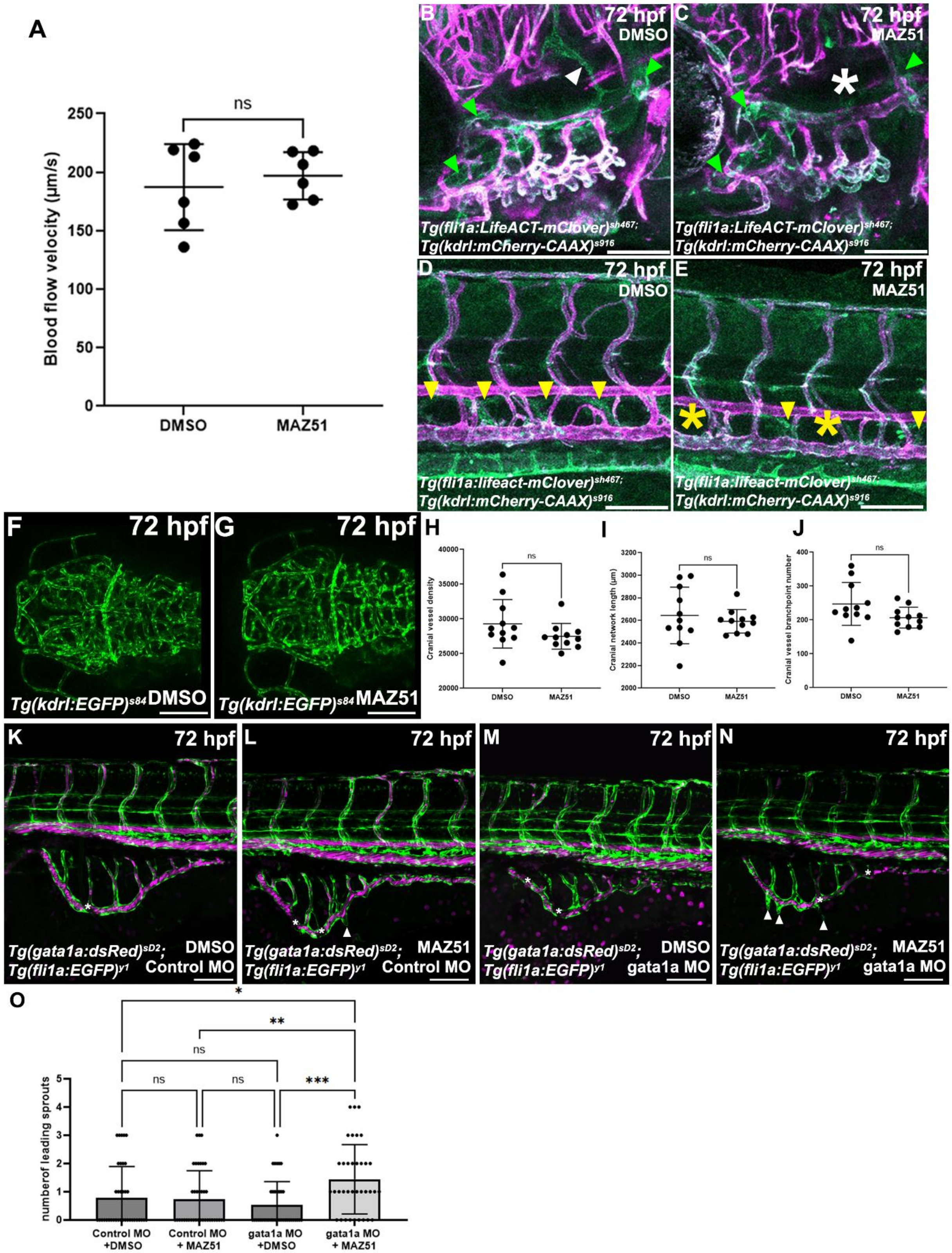

Fig. S15. Acute Flt4 inhibition impairs leading sprout regression under reduced-viscosity flow conditions.

A) MAZ51 treatment does not significantly alter blood flow velocity. Unpaired *t*-test; ns, not significant. Two independent repeats; DMSO, n=6; MAZ51, n=6.**B-C)** Treatment with MAZ51 from 56-72 hpf causes craniofacial lymphatic defects consistent with reduced Flt4 signalling. Yellow arrowheads indicate lymphatic vessels: a loss of otolithic facial lymphatic vessel (white arrowhead/asterisk), and reductions in lateral facial lymphatic vessel formation (green arrowheads); asterisks indicate lymphatic defects. **D-E)** MAZ51 treatment from 56-72 hpf disrupts thoracic duct formation. Yellow arrowheads indicate the thoracic duct; asterisks indicate regions of defective thoracic duct formation. **F-J)** cranial vascular network formation is not significantly affected by Maz51 treatment, with no significant difference in cranial vascular density, network length and branchpoint number observed. Two independent repeats; DMSO, n=12; MAZ51, n=11. **K-N)** Representative images of embryos injected with control or *gata1a* morpholino and treated with DMSO or MAZ51 from 56-72 hpf. White arrowheads indicate retained leading sprouts. White asterisks indicate pigmentation. **O)** Combined *gata1a* knockdown and MAZ51 treatment significantly increases retained leading sprout number compared with all other conditions. Two-Way ANOVA with multiple comparisons; * $p<0.5$; ** $p \leqslant 0.01$; ***$p \leqslant 0.001$; ns, not significant. Three independent repeats; control MO + DMSO, n=38; control MO + MAZ51, n=38; *gata1a* MO + DMSO, n=38; *gata1a* MO + MAZ51, n=36. Scale bars: 100μm.

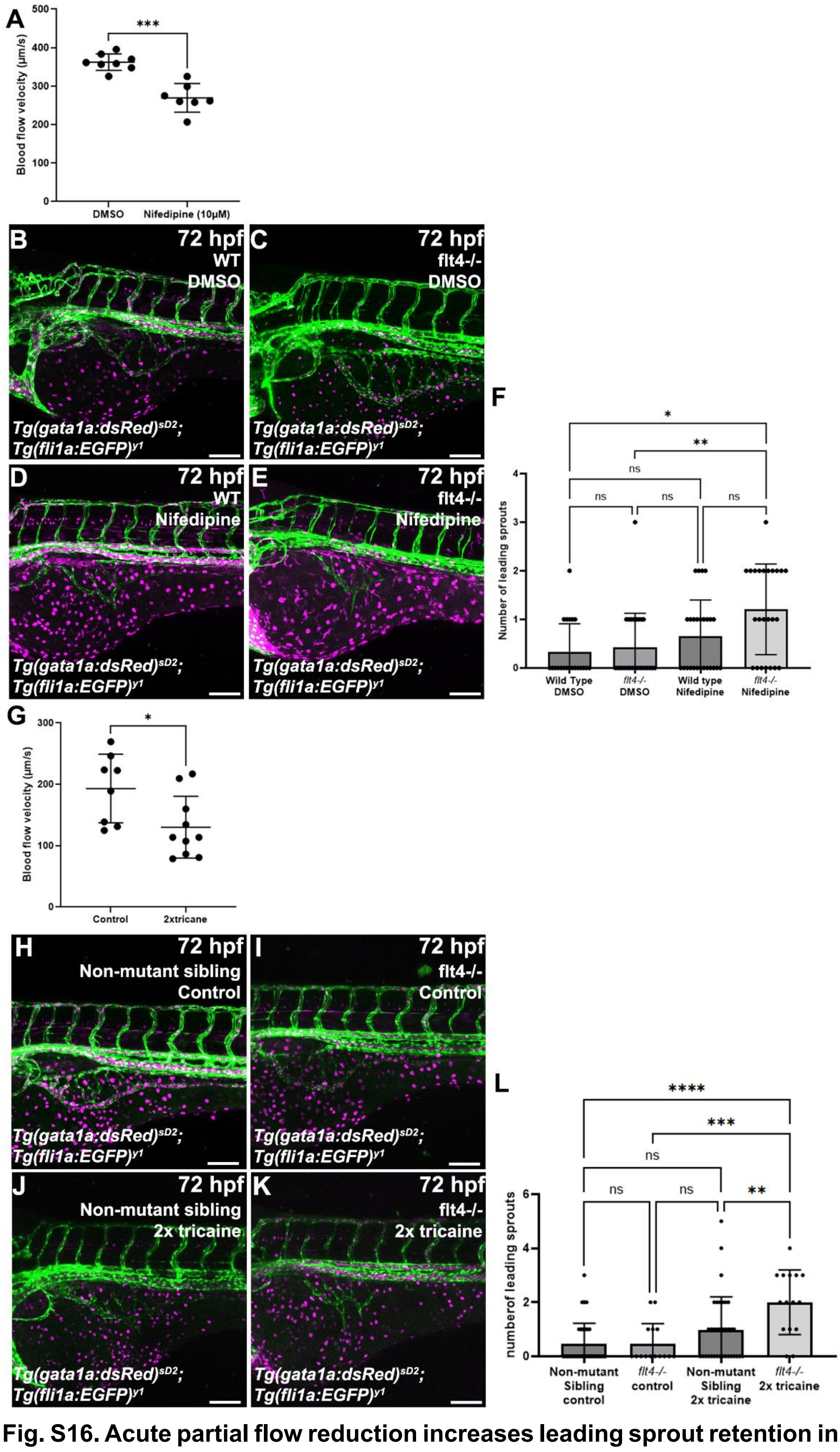

Fig. S16. Acute partial flow reduction increases leading sprout retention in *flt4*^{qmc316} mutants

Development • Supplementary information

A) Treatment with 10µM nifedipine significantly reduces blood flow velocity without abolishing circulation. Unpaired *t*-test; *** $p \leqslant 0.001$. Two independent repeats; DMSO n=8; nifedipine n=7. **B-E)** Representative images of the SIVP in wild-type and *flt4*^{qmc316} mutant embryos treated with DMSO or 10µM nifedipine from 56-72 hpf. **F)** Acute partial flow reduction increases retained leading sprout number in *flt4*^{qmc316} mutants compared with DMSO-treated controls. WT nifedipine-treated embryos showed an intermediate response that was not significantly different from *flt4*^{qmc316} nifedipine treated embryos. Two-way ANOVA with multiple comparisons; * $p \leqslant 0.05$; ** $p \leqslant 0.01$; ns, not significant. Three independent repeats; WT DMSO, n=21; *flt4*$^{qmc31\ 6}$DMSO, n=26; WT nifedipine n=26; *flt4*^{qmc316} nifedipine, n=24. Scale bars: 100µm. **G)** Treatment with 12x tricaine significantly reduces blood flow velocity without abolishing circulation. Unpaired *t*-test; * $p \leqslant 0.05$. Two independent repeats; control n=8; 2x tricaine n=10. **H-I)** Representative images of the SIVP in control or 2x tricaine-treated wild-type and *flt4*^{qmc316} mutant embryos. **F)** Acute partial flow reduction increases retained leading sprout number in 2x tricaine-treated *flt4*^{qmc316} mutants compared other conditions. Two-way ANOVA with multiple comparisons; ** $p \leqslant 0.01$; *** $p \leqslant 0.001$; **** $p \leqslant 0.0001$; ns, not significant. Two independent repeats; sibling control n=42; *flt4*^{qmc316} control n=15; WT 2x tricaine n=35; *flt4*^{qmc316} 2x tricaine, n=15. Scale bars: 100µm.

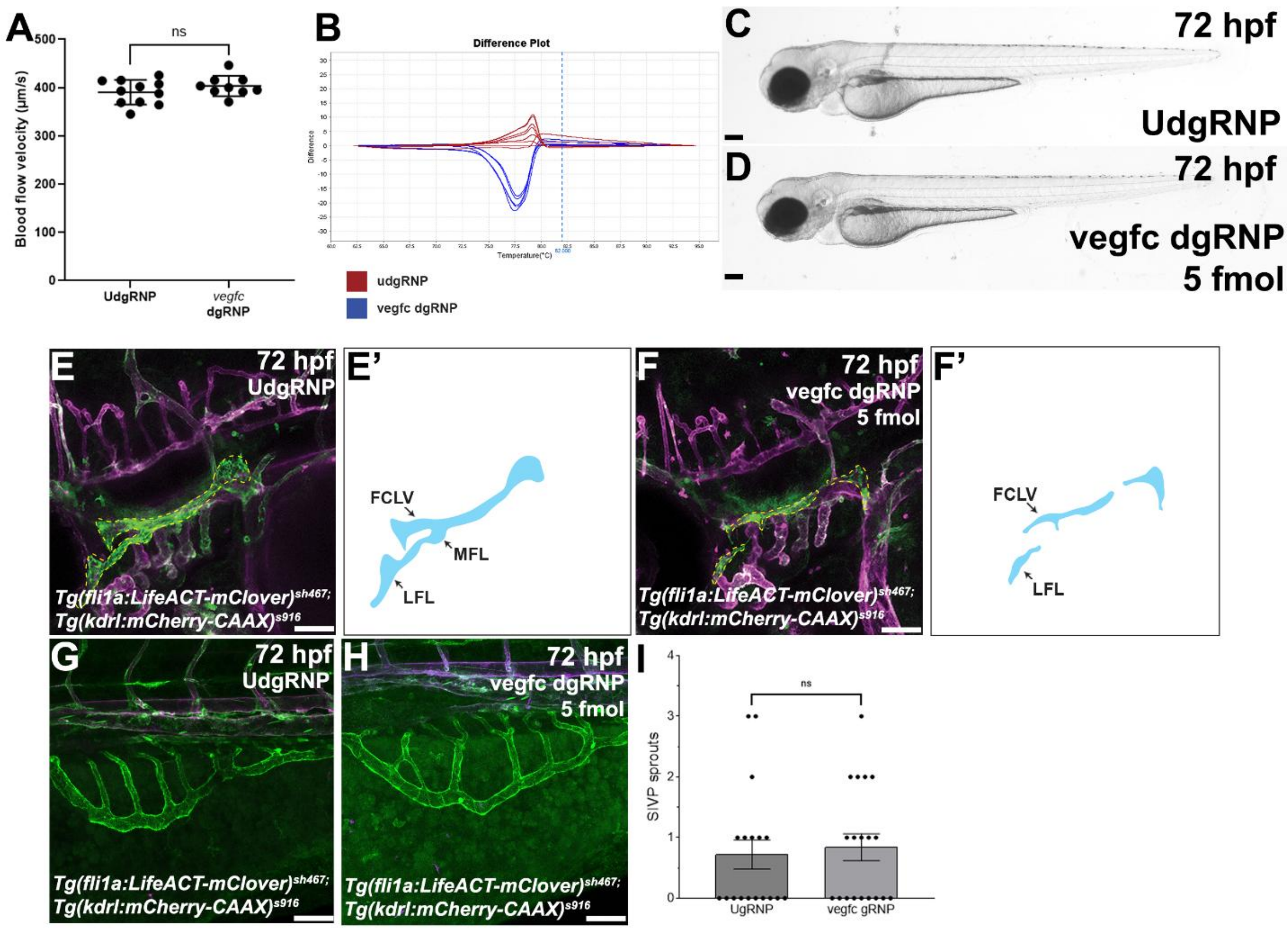

Fig. S17. *vegfc* disruption does not affect leading sprout regression

A) *vegfc* dgRNP injection does not significantly alter blood flow velocity. Unpaired *t*-test; ns, not significant. Two independent repeats; uninjected/control gRNP, n = 11; *vegfc* gRNP, n = 9.**B)** High-resolution melt analysis confirms genomic mutation in *vegfc* gRNP-injected embryos. **C,D)** *vegfc* crispants do not display oedema or gross morphological defects compared with control gRNP-injected embryos. **E–F')** *vegfc* crispants display impaired facial lymphatic formation compared with controls, confirming effective disruption of *vegfc* function. FCLV, facial collecting lymphatic vessel; MFL, medial facial lymphatic vessel; LFL, lateral facial lymphatic vessel. **G,H)** SIVP morphology appears comparable between control and *vegfc* crispant embryos. **I)** Leading SIVP sprout number is not significantly different between control and *vegfc* crispant embryos. Mann-Whitney *U*-test; ns, not significant. Two independent repeats; universal control non-targeting dgRNP (UdgRNP), n = 19; *vegfc* gRNP, n = 20. Scale bars: 100 µm in C,D; 50 µm in E–H

Table S1. Sequence of primers, crRNAs, and morpholinos used in this study

Name	Sequence (5'-3')	Reference
**tnnt2a-1 (exon 7) crRNA**	CCGTAAGCGCATGGAGAAGGCC	(Wu et al., 2018)
**tnnt2a-2 (exon 11) crRNA**	GCGTTCTCCTCCTGCTGAGCC	(Wu et al., 2018)
**tnnt2a-3 (exon 5) crRNA**	CCTTGCGAGCTCTTTCCTCC	(Wu et al., 2018)
**tnnt2a-4 (exon 3) crRNA**	CAATTCCTTTGCTTTCTCTC	(Wu et al., 2018)
**Standard Control MO**	CCTCTTACCTCAGTTACAATTTATA	Gene Tools, LLC
***tnnt2a* MO**	CATGTTTGCTCTGATCTGACACGCA	(Sehnert *et al.*, 2002)
***flt4* MO**	CTCTTCATTTCCAGGTTTCAAGTCC	(Hogan *et al.*, 2009b)
***gata1a* MO**	CTGCAAGTGTAGTATTGAAGATGTC	(Galloway *et al.*, 2005)
**Universal control crRNA**	GGGAGGCGTTCGGGCCACAGGTTTTAGAGCT ATGCT	
***Vegfc* crRNA**	AGCAGCACTCAATCATACGA	This study

Table S2. Mean endothelial cell number within the SIVP in the presence and absence of blood flow. ECs within the SIVP were divided into three subsets (tip, SIV, branch) according to their initial position in the SIVP at the beginning of cell tracking (56hpf). The average number of ECs per embryo were quantified at the start and end of tracking. Mean EC proliferation events per EC subset were recorded. EC migration between subsets were quantified. Total ECs per subset comprise the sum of ECs at the start and mean EC proliferation events observed during time lapses (control, n=7 embryos, *tnnt2a* morphants, n=6 embryos. Values are mean ±SD).

Control	Start (56hpf)	Proliferation events	Total ECs	ECs lost to other subsets	ECs gained from other subsets	End (72hpf)
Tip	9 ±3	4 ±3	13 ±3	10 ±2	0 ±0	3 ±3
SIV	21 ±3	8 ±5	29 ±4	10 ±4	10 ±2	29 ±3
Branch	17 ±7	8 ±4	25 ±6	0 ±1	10 ±4	35 ±11
Total	48 ±9	19 ±9	68 ±9			68 ±13
***tnnt2a* MO**	**Start (56hpf)**	**Proliferation events**	**Total ECs**	**EC contribution to other subsets**	**ECs gained from other subsets**	**End (72hpf)**
Tip	13 ±5	7 ±0	20 ±2	5 ±2	4 ±3	19 ±5
SIV	19 ±3	7 ±1	26 ±2	10 ±5	6 ±3	22 ±8
Branch	9 ±5	5 ±0	14 ±2	2 ±1	6 ±4	18 ±9
Total	41 ±8	19 ±1	59 ±5			59 ±14

Table S3. Percentage proportions of endothelial populations in relation to total endothelial numbers within the SIVP in the presence and absence of blood flow. ECs within the SIVP were divided into three subsets (tip, SIV, branch) according to their initial position in the SIVP at the beginning of cell tracking (56hpf). The average number of ECs per embryo were quantified at the start and end of tracking. Mean EC proliferation events per EC subset were recorded and percentage of total EC number was calculated. Total ECs per subset comprise the sum of ECs at the start and mean EC proliferation events observed during time lapses (control, n=7 embryos, *tnnt2a* morphants, n=6 embryos. Values are mean ±SD).

**Control**	**Start (56hpf)**	**Proliferation events**	**Total ECs**	**End (72hpf)**
**Tip**	9 ±3(18.8±6.25 %)	4 ±3 (21.1±15.7%)	13 ±3 (21.1±15.7%)	3 ±3 (15.7±15.7%)
**SIV**	21 ±3 (43.8±6.25%)	8 ±5 (42.1±26.3%)	29 ±4 (21.1±15.7%)	29 ±3 (42.6±15.7%)
**Branch**	17 ±7 (35.4±14.5%)	8 ±4	25 ±6 (21.1±15.7%)	35 ±11 (52.1±16.2%)
**Total**	48 ±9	19 ±9	68 ±9	68 ±13
***tnnt2a* MO**	**Start (56hpf)**	**Proliferation events**	**Total ECs**	**End (72hpf)**
**Tip**	13 ±5 (31.7±12.1%)	7 ±0 (36.8±0%)	20 ±2 (33.9±3.4%)	19 ±5 (32.2±8.5%)
**SIV**	19 ±3 (46.3±7.3%)	7 ±1 (36.8±5.3%)	26 ±2 (44.1±3.4%)	22 ±8 (37.3±13.6%)
**Branch**	9 ±5 (21.9±12.1%)	5 ±0 (26.3±0%)	14 ±2 (23.7±3.4%)	18 ±9 (30.1±15.3%)
**Total**	41 ±8	19 ±1	59 ±5	59 ±14

Development • Supplementary information

Table S4. Percentage change in endothelial populations within the SIVP in the presence and absence of blood flow. ECs within the SIVP were divided into three subsets (tip, SIV, branch) according to their initial position in the SIVP at the beginning of cell tracking (56hpf). The average number of ECs per embryo were quantified at the start and end of tracking. Mean EC proliferation events per EC subset were recorded and percentage of ECs lost and gained were calculated relative to total EC mean. Total ECs per subset comprise the sum of ECs at the start and mean EC proliferation events observed during time lapses (control, n=7 embryos, *tnnt2a* morphants, n=6 embryos. Values are mean ±SD).

**Control**	**Total ECs**	**ECs lost to other subsets**	**ECs gained from other subsets**	**End (72hpf)**
**Tip**	13 ±3	10 ±2 (76.9±15.3%)	0 ±0 (0±0%)	3 ±3 (23.1±23.1%)
**SIV**	29 ±4	10 ±4 (34.5±13.8%)	10 ±2 (34.5±6.9%)	29 ±3 (100±10.3%)
**Branch**	25 ±6	0 ±1 (0±4%)	10 ±4 (40±16%)	35 ±11 (140±44%)
**Total**	68 ±9			68 ±13
***tnnt2a* MO**	**Total ECs**	**EC contribution to other subsets**	**ECs gained from other subsets**	**End (72hpf)**
**Tip**	20 ±2	5 ±2 (25±10%)	4 ±3 (20±15%)	19 ±5 (95±25%)
**SIV**	26 ±2	10 ±5 (38.5±19.2%)	6 ±3 (23.1±11.5%)	22 ±8 (84.6±30.1%)
**Branch**	14 ±2	2 ±1 (14.3±7.1%)	6 ±4 (42.9±28.6%)	18 ±9 (128.6±64.3%)
**Total**	59 ±5			59 ±14

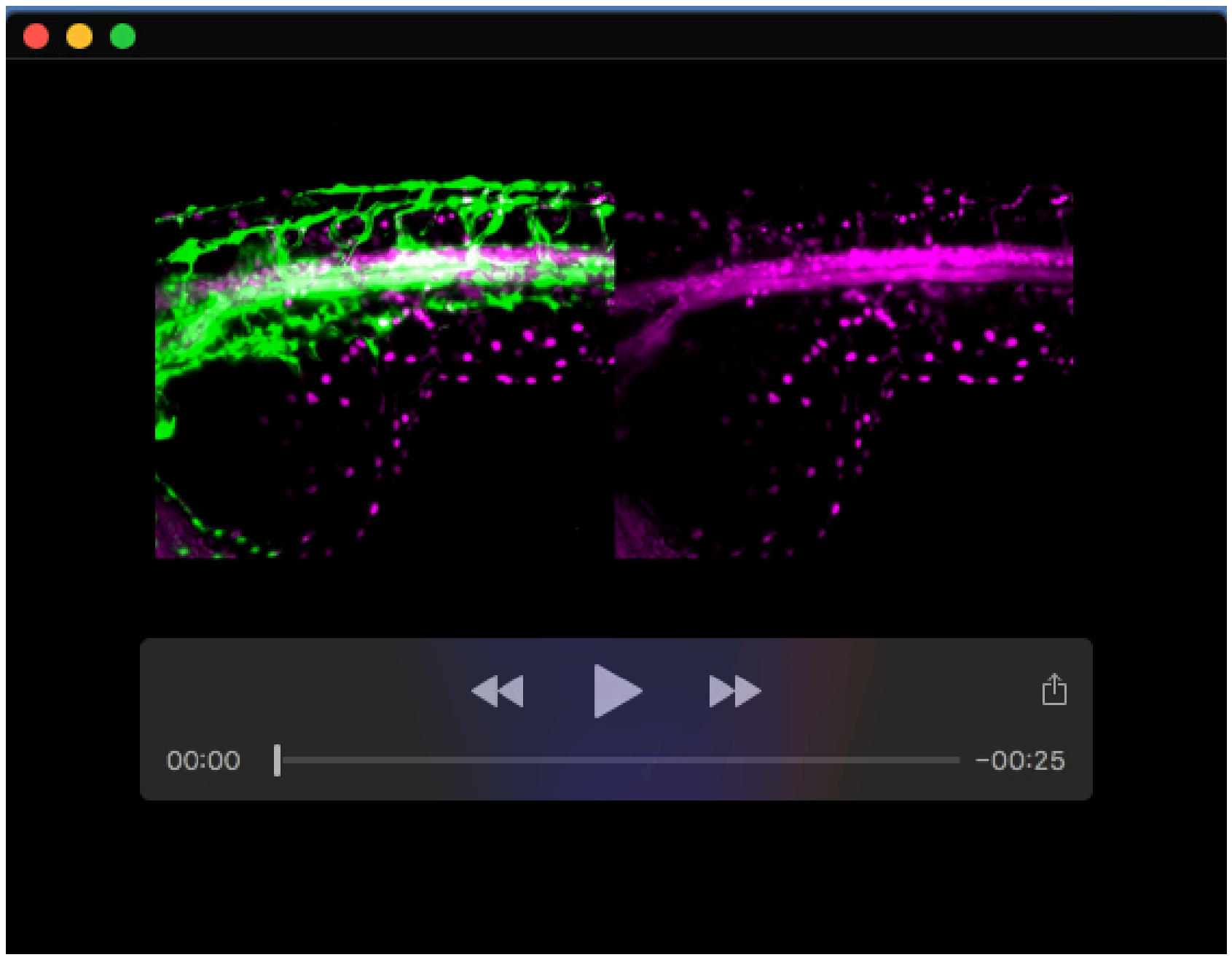

Movie 1. SIVP perfusion is coincident with leading sprout regression

Time-lapse movie of *Tg(fli1a:egfp)*^{y1};*(gata1a:DsRED)*^{sD2} labelling endothelial cells (green) and erythrocytes (magenta). Images were taken from 53hpf for approximately 19 hours at 5-minute intervals. Z-series were flattened to 2D maximum intensity projections and presented in time series. Arrows highlight points at which blood flow enters different segments of the SIVP from anterior to posterior.

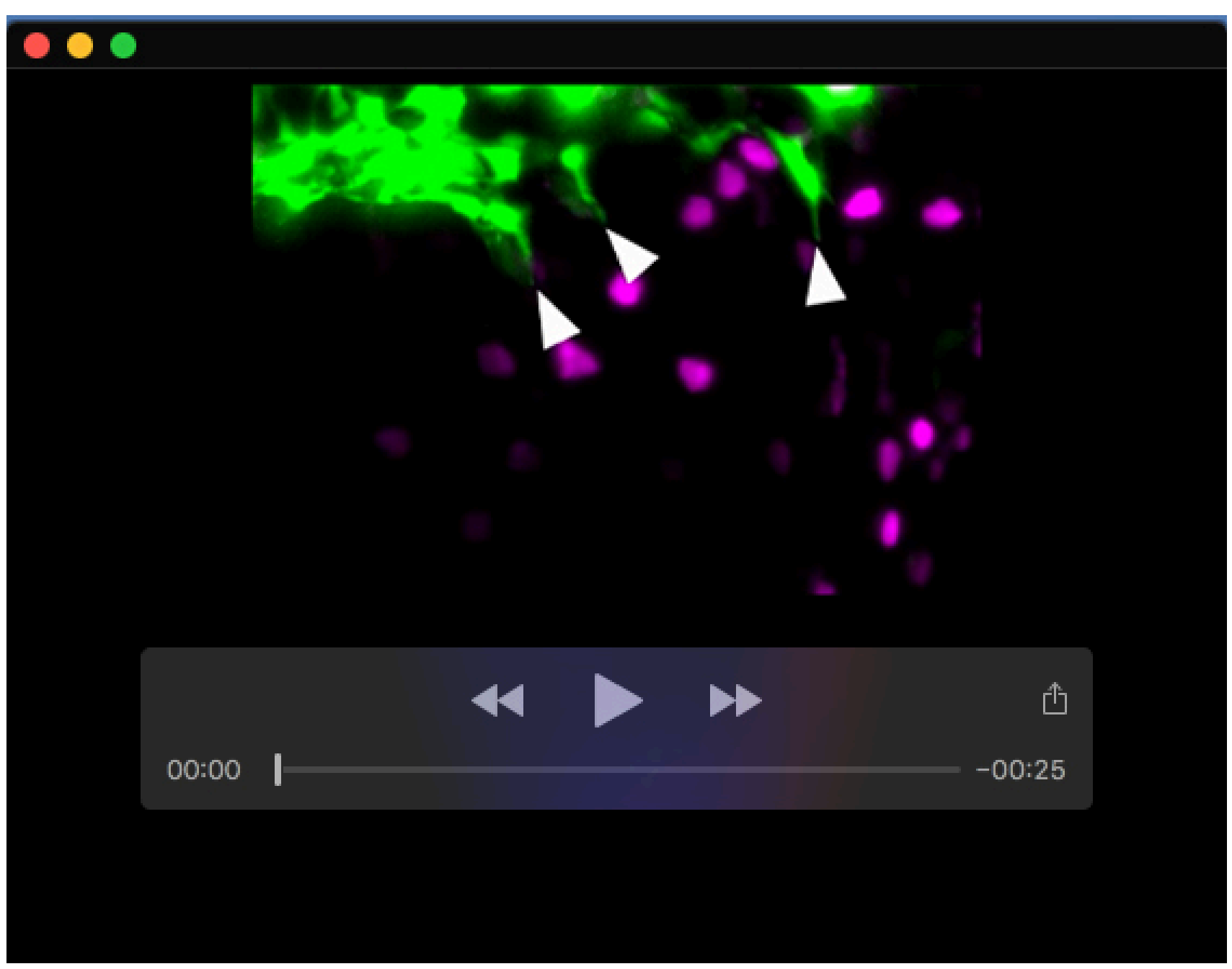

Movie 2. Leading sprout regression occurs in the presence of blood flow

Time-lapse movie of *Tg(fli1a:egfp)*^{y1};*(gata1a:DsRED)*^{sD2} labelling endothelial cells (green) and erythrocytes (magenta). Movie is enlarged and cropped from Movie 1. Images were taken from 53hpf for approximately 19 hours at 5-minute intervals. Arrowheads highlight leading sprouts within the SIVP undergoing regression in the presence of blood flow. The two most anterior sprouts (left side of plexus) anastomose, while the other three undergo flow-mediated regression.

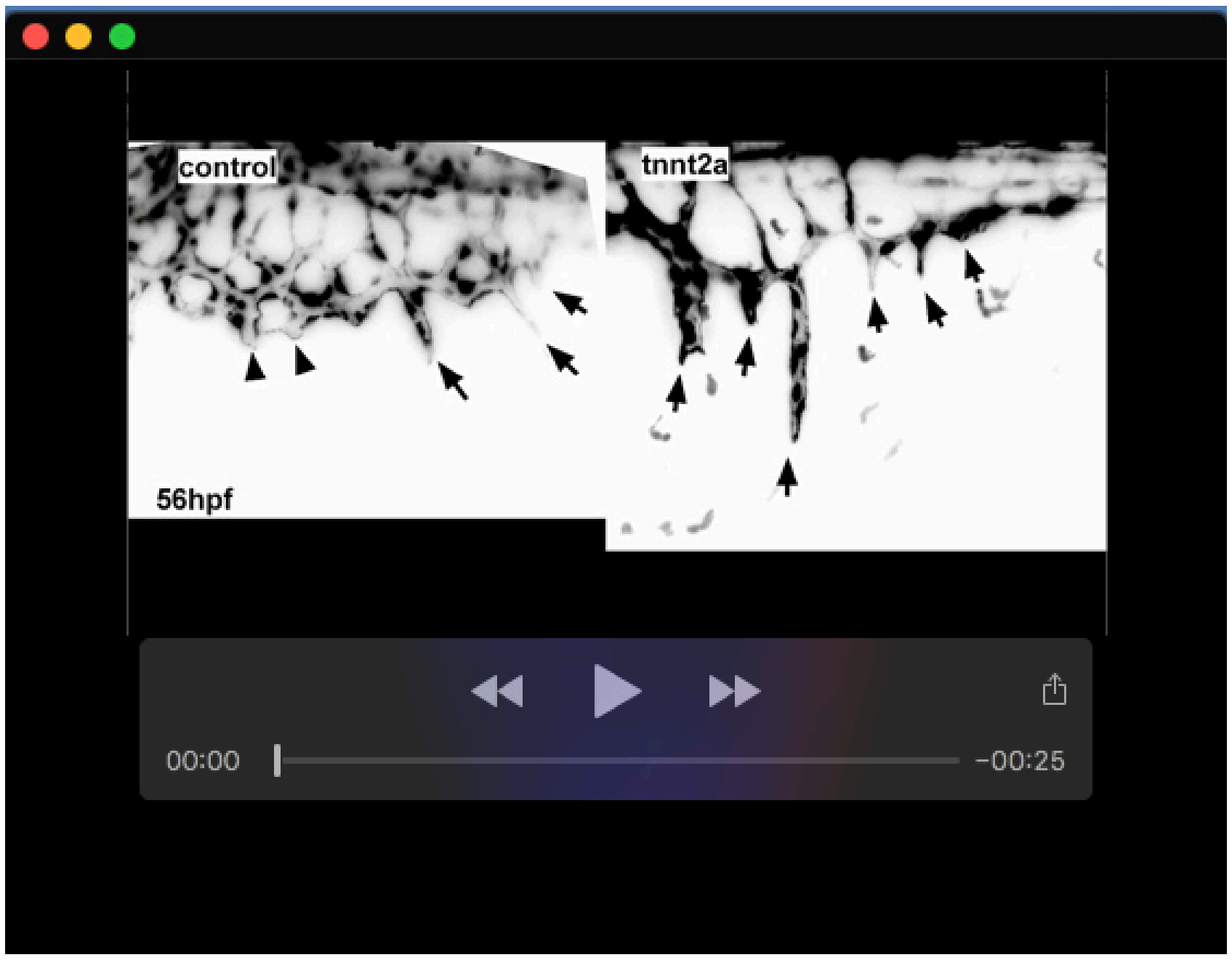

Movie 3. SIVP Leading sprouts fail to regress in *tnnt2a* morphants

Time-lapse movie of *Tg(fli1a:egfp)*^{y1} *control* and *tnnt2a* morphants. Images were taken at 5-minute intervals from 56hpf for approximately 14 hours and were down sampled to 30-minute intervals. Z-series were flattened to 2D maximum intensity projections and presented in time series. Arrowheads highlight leading sprouts within the SIVP undergoing regression in the presence of blood flow or failing to regress in the absence of blood flow.

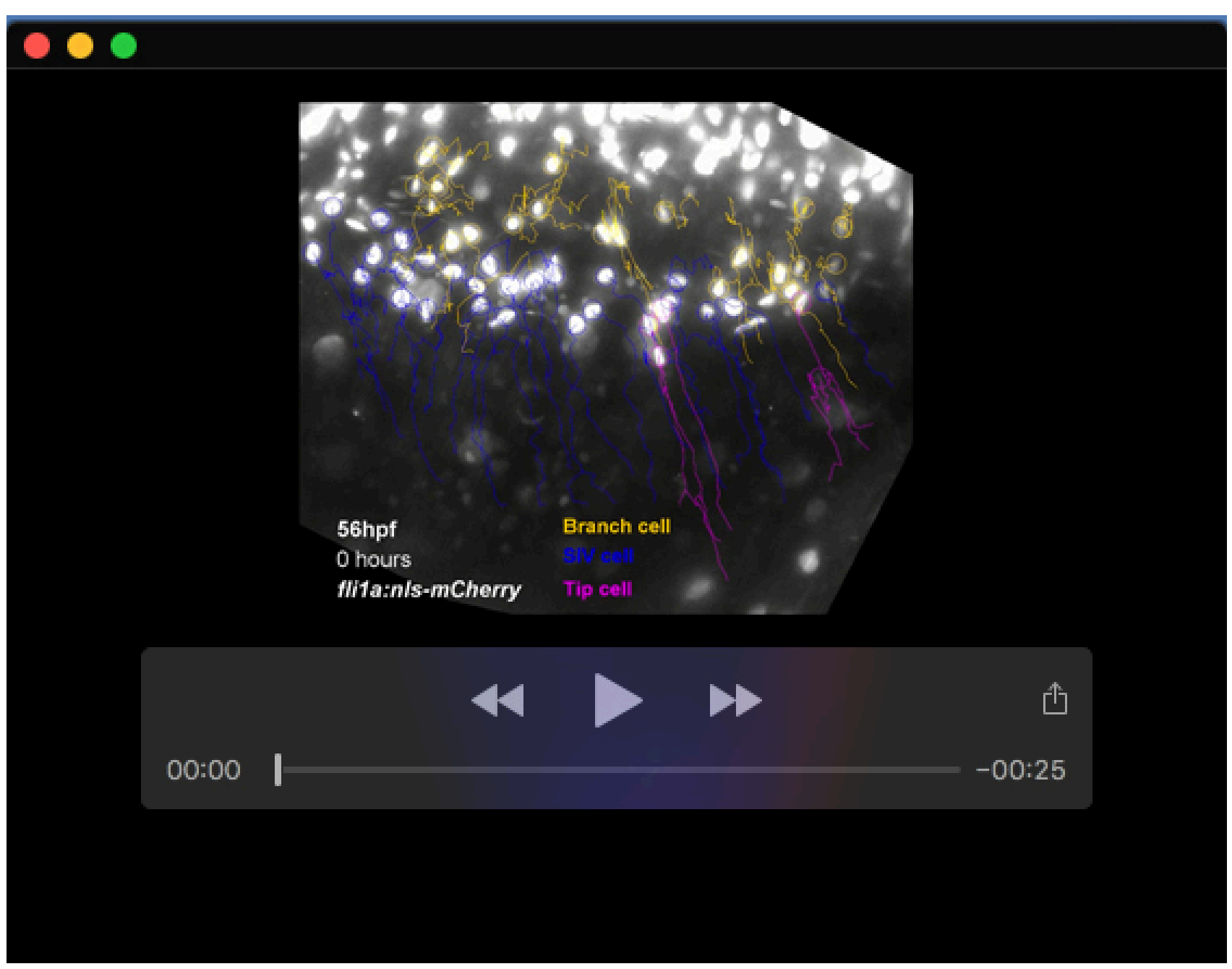

Movie 4. Cell migration trajectories in *control* morphants

Time-lapse movie of *Tg(fli1a:nls-mCherry)*^{SH370} overlaid with cell migration trajectories in control morphants. Images were taken for approximate 14 hours, 30-minute interval, starting at 56hpf. Z-series were flattened to 2D maximum intensity projections and presented in time series. Images were processed using TrackMate Plugin in Fiji (ImageJ). Orange, branch cells; blue, SIV cells; magenta, tip cells.

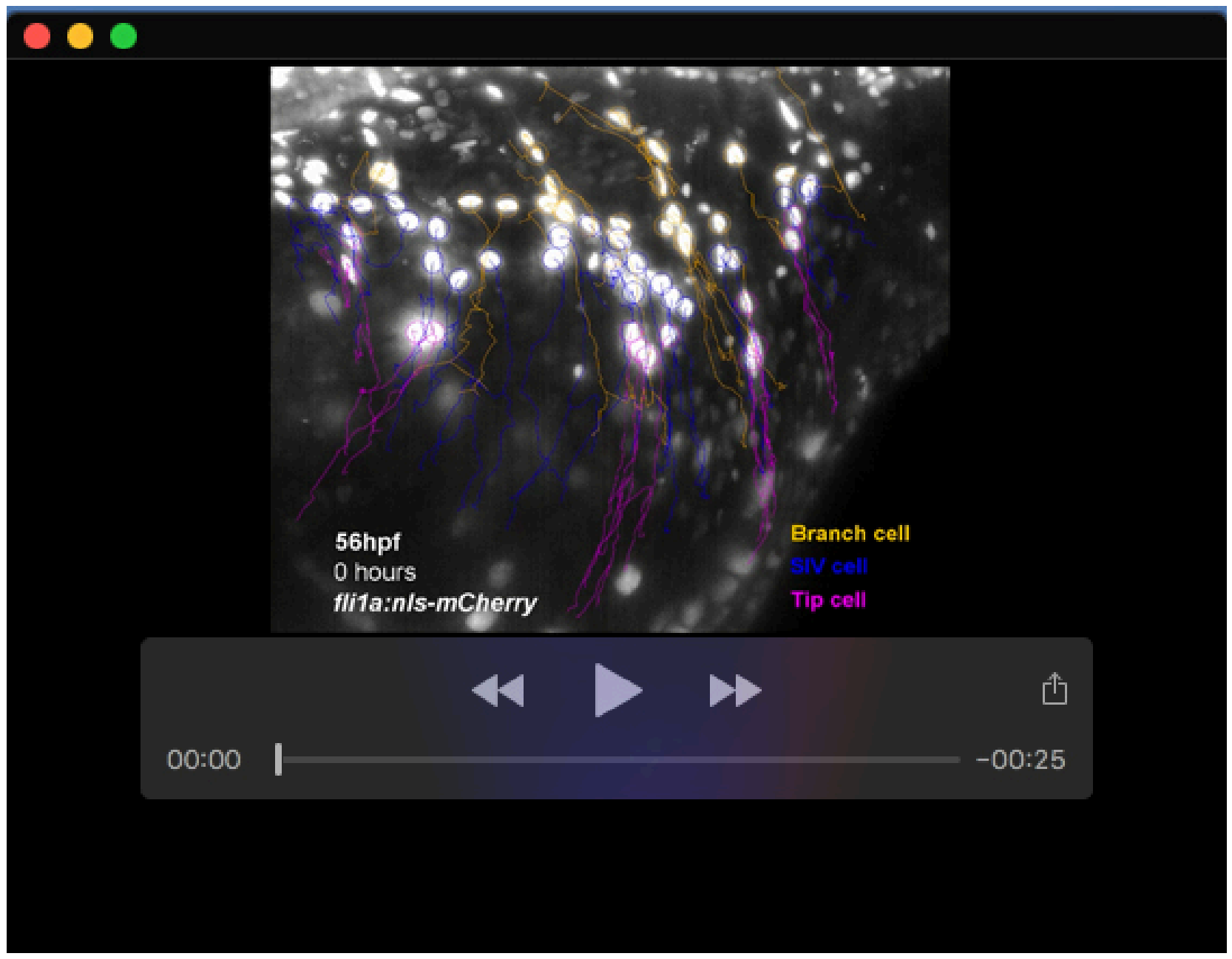

Movie 5. Cell migration trajectories in *tnnt2a* morphants

Time-lapse movie of *Tg(fli1a:nls-mCherry)*^{SH370} overlaid with cell migration trajectories in *tnnt2a* morphants. Images were taken for approximate 14 hours, 30-minute interval, starting at 56hpf. Z-series were flattened to 2D maximum intensity projections and presented in time series. Images were processed using TrackMate Plugin in Fiji (ImageJ). Orange, branch cells; blue, SIV cells; magenta, tip cells.

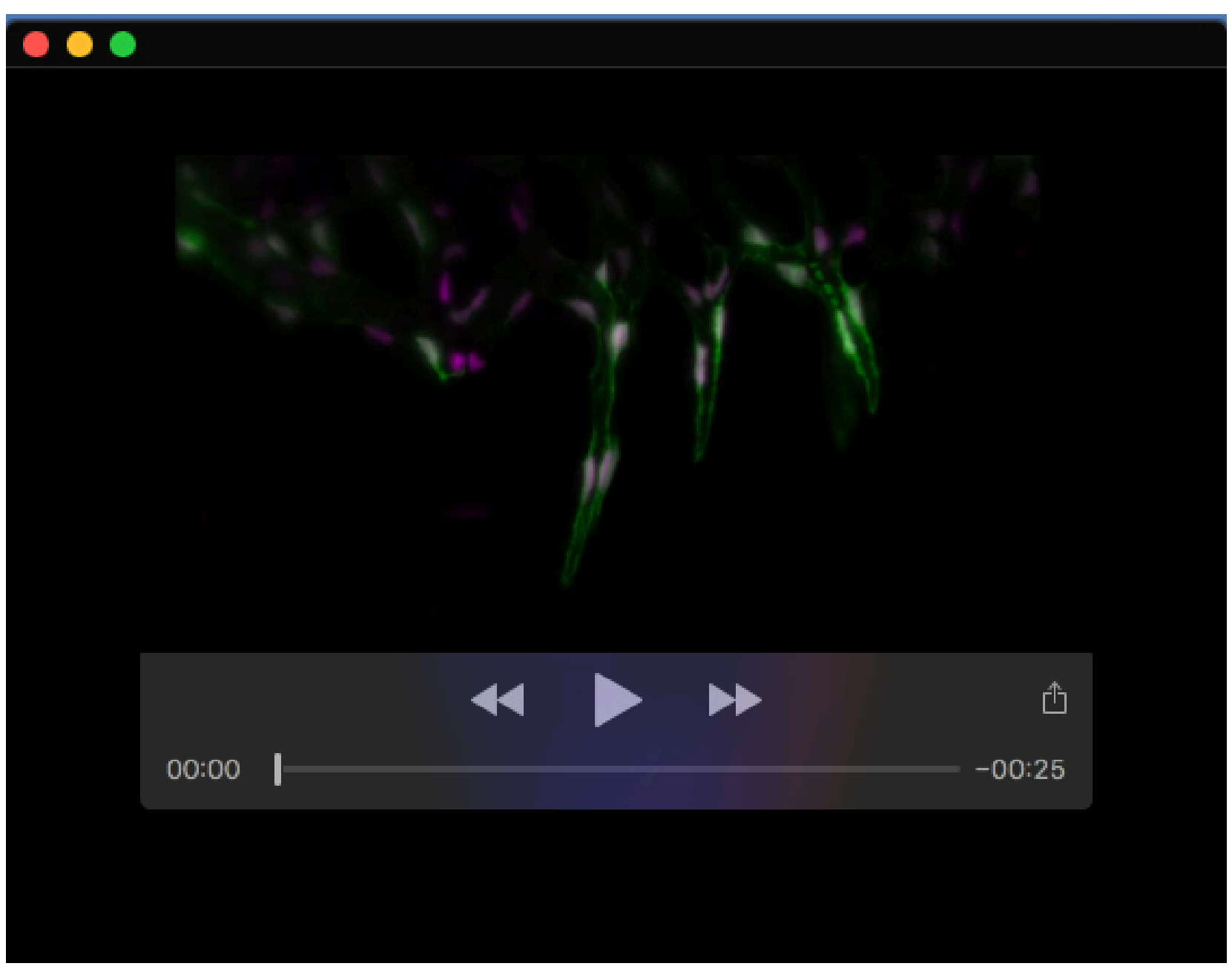

Movie 6. Daughter cells from the tip cells rapidly migrate towards SIV after cell division

Time-lapse movie of *Tg(fli1a:egfp)*^{y1};*(fli1a:nls-mCherry)*^{SH370} highlighting tip cell proliferation within leading sprouts and contribution of daughter cells to SIV. The daughter cell closest to SIV migrate rapidly towards SIV after cell division, leaving only one pair of ECs within the sprout. Images were taken for approximately 14 hours at 30-minute intervals, starting at 56hpf. Z-series were flattened to 2D maximum intensity projections and presented in time series. Images were processed using TrackMate Plugin in Fiji (ImageJ). Arrowheads indicate dorsal daughter cells.

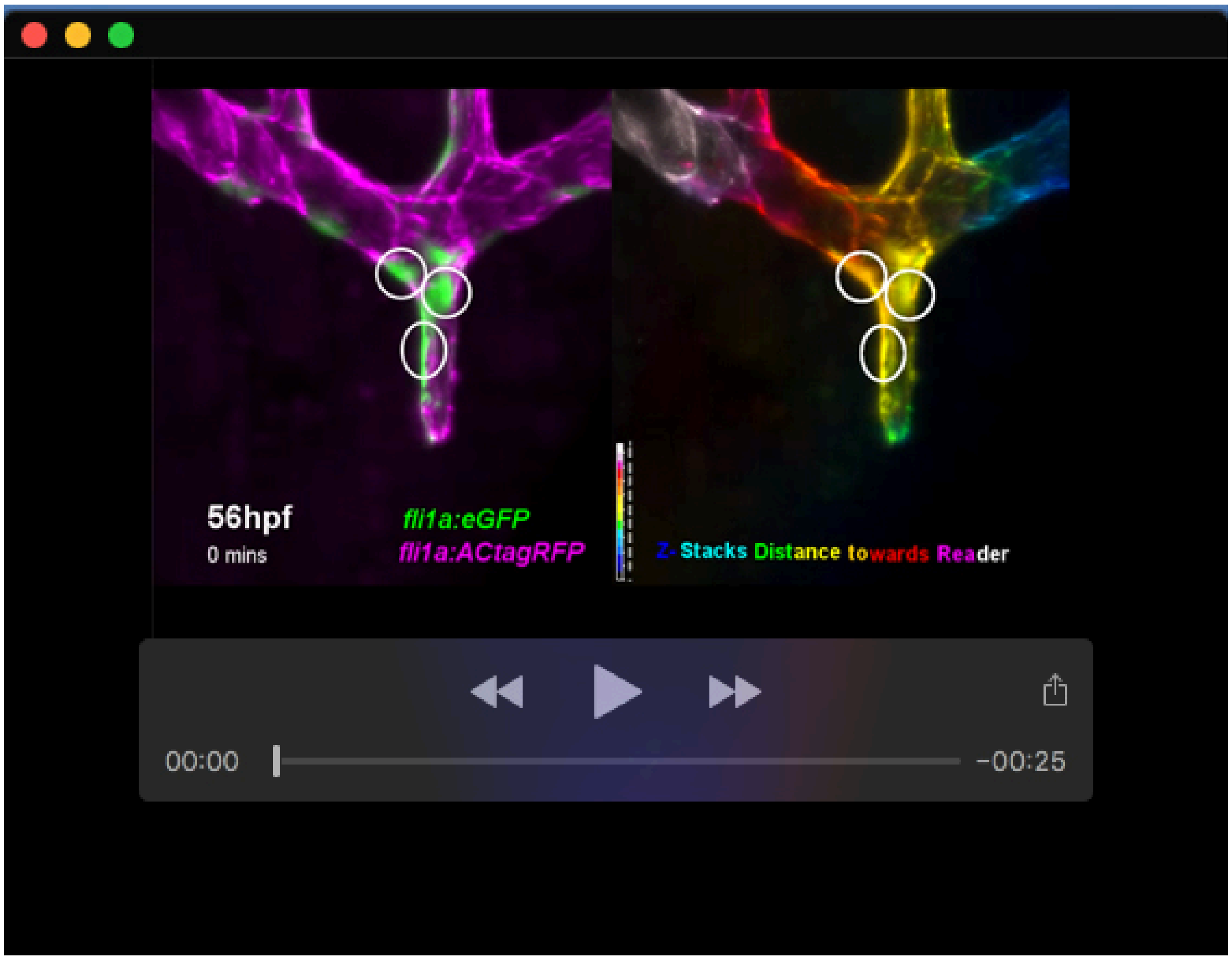

Movie 7. Endothelial cell rearrangement during sprout regression

Time-lapse movie of leading sprout regression in *Tg(fli1a:egfp)^{y1};(fli1a:AC-tagRFP)^{SH511}*. Images were taken from 56hpf 6 hours at 5-minute intervals. Z-series were flattened to 2D maximum intensity projections and presented in time series (left panel) and colour coded to illustrate depth, (right panel, white closest to viewer).
