# Supplementary material for: Blood flow coordinates collective endothelial cell migration during vascular plexus formation and promotes angiogenic sprout regression via flt4
Source: Development. 2026 Oct 6;153(19):dev205424. doi: 10.1242/dev.205424 (PMC13723401; doi:10.1242/dev.205424)
Supplement: Supplementary file 2 [file develop-153-205424_review_history.pdf]

Blood flow coordinates collective endothelial cell migration during vascular plexus formation and promotes angiogenic sprout regression via *flt4*

Yan Chen, Aaron M. Savage, Zhen Jiang, Ignacio Rodriguez-Pastrana, Myles Wilson, Hyejeong R. Kim, Paul C. Evans and Robert N. Wilkinson
DOI: 10.1242/dev.205424

Editor: Benoit G Bruneau

Review timeline

Original submission:	5 December 2025
Editorial decision:	1 January 2026
First revision received:	29 May 2026
Editorial decision:	15 June 2026
Second revision received:	25 August 2026
Accepted:	27 August 2026

Original submission

First decision letter

MS ID#: dev.205424

MS TITLE: Blood flow coordinates collective endothelial cell migration during vascular plexus formation and promotes angiogenic sprout regression via vegfr3/flt4

AUTHORS: Yan Chen; Aaron Michael Savage; Zhen Jiang; Hyejeong R. Kim; Paul C. Evans; Robert N. Wilkinson

Dear Dr Wilkinson,

I have now received all the referees' reports on the above manuscript, and have reached a decision. The referees' comments are appended below.

As you will see, the referees express considerable interest in your work, but have some significant criticisms and recommend a substantial revision of your manuscript before we can consider publication. If you are able to revise the manuscript along the lines suggested, which may involve further experiments, I will be happy to receive a revised version of the manuscript. Your revised paper will be re-reviewed by one or more of the original referees, and acceptance of your manuscript will depend on your addressing satisfactorily the reviewers' major concerns. Please also note that Development will normally permit only one round of major revision. If it would be helpful, you are welcome to contact us to discuss your revision in greater detail. Please send us a point-by-point response indicating your plans for addressing the referees' comments, and we will look over this and provide further guidance.

Please attend to all of the reviewers' comments and ensure that you upload both a 'clean' version of your Word file, along with a highlighted version clearly showing where you have made changes in the revised manuscript. Please avoid using 'Tracked changes' in Word files as these are lost in PDF conversion. I should be grateful if you would also provide a point-by-point response detailing how

© 2026. Published by The Company of Biologists under the terms of the Creative Commons Attribution License (https://creativecommons.org/licenses/by/4.0/).

you have dealt with the points raised by the reviewers in the 'Response to Reviewers' box. If you do not agree with any of their criticisms or suggestions please explain clearly why this is so.

Reviewer 1

The manuscript presents thorough phenotypic of SIVP development in zebrafish under differential flow conditions, with a specific focus on remodelling of this vascular bed. The authors report that blood flow instructs directed migration of ECs within the growing sprouts at the leading edge. Timelapse imaging to track proliferation reveals that following cell division at the leading edge (tip cells), one cell will migrate dorsally, against the direction of flow, whilst the other daughter remains at the tip. This makes for an even distribution of the ECs throughout the SIVP. In embryos without flow, directionality, and associated polarised Golgi positioning, is lost and there is an increase in the number of ECs that remain at the tip. Further application of a gata1 morpholino (depleting RBCs) suggests that regression does occur under conditions of reduced flow. To interrogate whether there is a role in VEGF signalling here, adjusting the flow set-point, the authors examined spatio-temporal expression of VEGF receptors (control vs no flow) in the SIVP. flt4 was differentially expressed and remained high in embryos without flow. The functional relevance of flt4 was shown in combination with gata1 morpholino knockdown; whilst both loss-of-function models individually did not lead to regression failure, when combined regression was compromised. This led to the conclusion that flt4 is required to regulate regression under low flow conditions.

The study is suited for publication and is likely to be of interest to the readership of Development. However, I have raised several comments below that I recommend are addressed prior to publication.

1. To model "low flow" the team has applied the previously validated gata1 morpholino. Whilst loss of circulating RBCs would undoubtedly lower shear on the endothelium, there is still fluid flow. Compared with tnnt2a knockdown, gata1 knockdown is therefore a much milder interference which likely explains why regression is not affected. I believe complementary approaches to model "low flow" would strengthen the conclusions. For example, temporal decrease or increase of flow using chemical antagonists/agonists during the time of SIVP rearrangements. Furthermore, the tnn2a morpholino can be titrated down to reduce flow (Vignes at al. Dev Cell 2022).

2. Comments above can be extended to the data presented in Figure 7, where flt4 is shown to be required for regression under low flow conditions. Based on the data presented in 7D, there are still a considerable number of embryos where vessels do regress in double LOF (flt4-/- and gata1 MO) and in only 1/18 embryos are there three sprouts left (as shown in G). To strengthen this data, I therefore strongly suggest the authors apply complementary approaches to alter flow in the flt4 mutants.

3. In gata1 morpholino injected embryos the vessel running along the SIVP plexus appears to have a wider lumen compared to controls (across all figures). Does this impact perfusion across the SIVP network? In a study by Sugden et al (NCB 2022) Engoglin was shown to sense flow and thereby coordinate cell shape changes so that vessel diameters are adjusted appropriately and all vessels in the trunk network are perfused. It would be beneficial if the authors conduct similar timelapse imaging of cellular behaviour (directionality) in the double LOF (flt4-/- and gata1 MO) embryos, as presented for the tnnta morphants (Fig 4-5), in conjunction with a vessel dye to label perfusion of regression.

4. Actin labelling in the AC-TagRFP line is not as defined as in other previously developed endothelial actin lines (lifeact or uthrophin). To faithfully monitor individual cells over time it would be worth repeating this data using these available lines.

5. There are dsRed+ve cells outside the vasculature across multiple figures. To assist readers, I suggest the authors should describe what these cells are in the text and point those out in the figure to clarify that these are not the cells of interest.

© 2026. Published by The Company of Biologists under the terms of the Creative Commons Attribution License (https://creativecommons.org/licenses/by/4.0/).

6. In movies 4 and 5 there are numerous tracks of individual cells which makes it very challenging to follow these cells/tracks over time. A cleaner representation of this data is requested.

Reviewer 2

SUMMARY OF THE ADVANCE MADE IN THIS PAPER AND ITS POTENTIAL SIGNIFICANCE TO THE FIELD

In this work, Chen et al. examine the development of the subintestinal vessels plexus (SIVP) in the zebrafish as a model for understanding how blood flow impacts endothelial cell (EC) migration and vascular remodeling. While development of this vascular bed from the posterior cardinal vein (PCV) has been extensively described in previous work, this paper focuses primarily on flow-mediated regression of the ventrally-directed "leading sprouts" that seem to pull the plexus over the yolk. The authors conclude that these cells polarize and migrate dorsally in response to blood flow and incorporate into the subintestinal vein (SIV), and that this behavior requires vegfr3/flt4, but only in conditions of low hematocrit/low viscosity/low shear stress. The fluorescent imaging data are nicely presented, and the work adds to our understanding of development of the SIVP. However, primary conclusions are not particularly novel. That is, it is well established that SIVP leading sprouts retract to the SIV, that blood flow induces vascular quiescence, that pruning/sprout retraction primarily occur via polarization and migration toward high flow or parent vessels, and that vegf3/flt4 plays a role in mechanosensing in low flow vessels. Additional analyses could add new mechanistic insight, strengthen conclusions, and increase the impact of this work.

SUGGESTIONS TO AUTHORS

Major comments

1. The authors state that SIVP perfusion occurs in an anterior-to-posterior fashion, and that blood flow triggers leading sprout regression. Accordingly, it would be informative to investigate whether there is a spatiotemporal correlation between blood flow and leading sprout regression.
2. Flow through the SIV causes EC elongation along the flow axis (based on nuclear morphology), and this change in shape and orientation might be sufficient to pull the sprout cells into the SIV wall. How can you distinguish between this pulling versus active migration of these cells, less than 50% of which are dorsal-polarized at 64 hpf? It would be informative correlate dorsal polarization with dorsal migration at the individual cell level, and/or to analyze EC shape changes in the SIV over time using the actin nanobody line.
3. In Figure 5, the step data plotted in panels H/I/K/L would be better analyzed by radial statistics. Binning these movements into dorsal, ventral, anterior, or posterior steps fails to fully represent the data. Notably, the graphs in J and M look remarkably similar, whereas the plots in H/I and K/M do not. In panel I, I see a confinement of migration along the DV axis, with less movement along the AP axis, but this isn't clear from the graph in panel J. Comparing panels K and L, I see very little change in tnnt2a MO versus control. I cannot appreciate that control cells display a tendency toward posterior migration while tnnt2a MO show significant anterior migration, as stated in the text.
4. Data presented do not support the authors' conclusions as stated in lines 328-336. In Figure 5, the temporal resolution is lost in data panels H-M. In panel N, where temporal resolution is preserved, only 3 cells are tracked over time for control and tnnt2a morphants. Accordingly, it is unclear how the authors can conclude from these data that in controls, there are differences in migratory behavior before and after flow. And in tnnt2a morphants, the authors state that SIV ECs migrate anteriorly, but panel N shows that the migration is mostly ventral and skews posteriorly, assuming that the axes are the same as in H/I/K/L.
5. In situ hybridization data (Figures S4, S6) are not publication quality. It is very difficult to see the SIVP due to very high background staining. Also, the images lack scale bars.
6. In the flt4 mutants + gata1 morpholino experiments, it would be helpful to assess SIV EC elongation and alignment against flow to help to differentiate between defective sprout EC dorsal "migration" versus defective "pulling" of sprout ECs to the SIV wall (see point 2 above)

Minor comments

© 2026. Published by The Company of Biologists under the terms of the Creative Commons Attribution License (https://creativecommons.org/licenses/by/4.0/).

1. The description of SIVP development could be improved. For example, line 66 states that the ECs from the primary SIV migrate dorsally to form branches and the SIA. But from published work (e.g. Hen et al. Development 2015) as well as provided movies, it is clear that some cells get "dropped off" during ventral migration from the PCV; these cells may move dorsally into the SIA.
2. Line 77 typo Zzebrafish
3. Line 97 change lumen to SIV
4. Line 117 construct synthesis is not in silico synthesis; maybe silicon-based DNA synthesis?
5. Line 171 sentence fragment
6. Movie 2 please annotate fusion event differently from sprout elongation and add timeframe to legend.
7. Figure 3 there is no reference to panels D-F in the text.
8. Line 222 typo: hereafterleading
9. In movies 4-6 that support Figure 4, it would be helpful to annotate at least some examples of ECs that move from one vessel segment to another as quantified in Figure 4D.
10. Figure 4D is confusing to me because it shows number of cell movements and the text discusses percent of cells moving, so it was hard to reconcile the figure and text.
11. In Figure 4 legend, remove the word "(magenta)" on line 786.
12. Movie 6: define the arrow in the legend as pointing to the dorsal daughter cell
13. Figure 5 define quadrants for polarization analysis in the legend and perhaps color code the datapoints in H,I,K,L to reflect A/P/D/V designations. In the legend, indicate timing of experiment (56-72 hpf?)
14. Line 347: It is not true that most tip cells showed ventral Golgi positioning at 56 hpf; this is the largest bin but it is less than 50%. Please rephrase.
15. Figure S7E: please label the X-axis to clearly define each experimental group. Consider a different statistical test for interaction.
16. Figure S2B diagram: replace flow with SIV and migration with sprout.
17. Figure S2C,D analysis requires categorical variable test like Fisher's exact
18. In Figure 7, the flt4 mutant SIVP seems to have abnormal morphology. Is this typical? Can the authors comment?
19. Line 492 sentence fragment

<u>Reviewer 3</u>

SUMMARY OF THE ADVANCE MADE IN THIS PAPER AND ITS POTENTIAL SIGNIFICANCE TO THE FIELD

In the manuscript by Chen et al, the authors use the subintestinal vessels (SIV) in zebrafish to investigate the role of blood flow in regulating vascular plexus formation. In particular, the authors find that regression of leading angiogenic sprouts at the vascular front of the SIV plexus is regulated by blood flow - under normal conditions, upon blood flow into SIVs, leading sprouts collapse and migrate back into the patent SIV. However, in the absence of flow (but not red blood cells) these sprouts contain more cells than normal and fail to migrate back into the main SIV. Interestingly, the authors find that flow-dependent regression is independent of apoptosis or changes in cell proliferation. They further note that reduction of VEGFC receptor (Vegfr3) limits regression, but only when red blood cell volume is simultaneously reduced. In general, these are interesting findings that modestly add to the literature on vascular regression - a fairly detailed analysis of SIV morphogenesis has been provided by several previous papers, though more detailed analysis on the role of flow is provided here. Further, there are several issues that may limit the potential impacts of these findings, especially regarding the genetic interaction between flt4 and flow. Specific comments are listed below.

SUGGESTIONS TO AUTHORS

Major comments/issues

All of the manipulations here involve blocking gene function from beginning of development, while phenotypic assessment is not until much later. Since SIV formation and plexus remodeling is a relatively late event (past 2 dpf), it is not possible to rule out that the observed phenotypes are simply downstream effects. While it is challenging to acutely regulate RBC volume, there are several ways to regulate flow at later time points - cardiac ablation (e.g. via laser), "cauterization"

© 2026. Published by The Company of Biologists under the terms of the Creative Commons Attribution License (https://creativecommons.org/licenses/by/4.0/).

(also via laser) of the SIV to block flow, or treatment with high dose tricaine. The authors need to incorporate any of these to verify that their results are due to flow acting at the stage they are proposing, at least regarding regression (I would not recommend using BDM to stop the heart as that can also affect pre-flow angiogenesis). Flt4 can be similarly inhibited via a selective small molecule inhibitor at the appropriate dose (MAZ51 or similar) and stage.

Lines 82-83, 392-393. The Baeyens et al. paper is a bit over-interpreted. The effect in zebrafish is only on the dorsal aorta (which actually lacks flt4 expression), while the artery remodeling effect is in mouse. Further, definitive genetic data (e.g. alleles that specifically block the proposed mechanosensory role of Vegfr3) are lacking - all of the zebrafish studies in that paper were using morphants including "high dose" experiments. Finally, the "shear stress setpoint" was determined based on flow chamber studies in vitro without any RBCs.

Line 227, 377. The term "perfusion" generally refers to any constituent of the blood, including red blood cells (RBCs). The leading sprouts appear to be lumenized and may be in contact with other blood constituents. This is probably worth checking (via injection with a low molecular weight fluorescence tracer - e.g. QDots) to determine whether these sprouts are or are not "perfused" in some way.

Line 387. ". . under low flow conditions. . .". This is a bit confusing and/or vague. Gata1a morphants have "flow" - they just have reduced RBC volume and everything else in circulation should largely be the same. Underscoring this fact is that the severe depletion of RBCs did not have a significant effect on SIV sprouts, suggesting some other flow component is playing a role. This "blooid flow" concept is vague in other places as well and should probably be more precisely defined.

I did not see any movies of the tnnt2a morphants. These should be included.

The epistasis between flt4 and gata1a is potentially interesting. However, there are a number of issues here: 1. The flt4 mutant may not be a null. Several different flt4 alleles have been reported that all have edema by 5 dpf - the mutant described here does not, suggesting it may be hypomorphic, possibly due to compensation or exon skipping. There are in-frame deletion alleles available that specifically target Vegfc binding that would be more informative, as would confirmed null alleles. 2. Similarly, gata1a knockdown is also hypomorphic, confounding any interpretation of the apparent epistasis. 3. Lacking genetic analysis of Vegfc. This is important since the flt4 allele here would be expected to affect Vegfc-dependent signaling. Would also be helpful to know pattern of vegfc in normal and gata1a-deficient embryos.

Are there any changes in vegfaa/b expression adjacent to SIVs in the absence of flow or RBCs?

More comprehensive analysis of flt4 mutant SIVs is needed (similar to the analysis done for control vs. tnnt2 MO) to confirm that there are not earlier SIV defects, or subtle defects.

Minor comments

Lines 53-55. There are a number of context-dependent examples of vascular regression and associated pathways that are regulated in those cases. It clearly is different across blood vessel types, but it is not a complete black box in many cases.

In some cases, there appear to be gaps in the SIVs (e.g. Figure 7). I assume these are due to pigment cells? How were these accounted for in the context of quantification? Why are they only apparent in some images? Were embryos usually treated with PTU? If so, this should be noted.

First revision

Author response to reviewers' comments

© 2026. Published by The Company of Biologists under the terms of the Creative Commons Attribution License (https://creativecommons.org/licenses/by/4.0/).

We thank the reviewers for their constructive comments and careful assessment of our manuscript. In response, we have substantially revised the manuscript and added new experiments and analyses to strengthen the central conclusions. In particular, we have performed orthogonal acute perturbation experiments to test the temporal requirement for blood flow and Flt4 activity during the sprout regression window. These include nifedipine treatment to acutely abolish or reduce flow from 56–72 hpf, MAZ51 treatment to inhibit Flt4/VEGFR3 activity during the same period, and mosaic CRISPR targeting of *vegfc* to assess the contribution of canonical Vegfc signalling. We have also added new analyses of *flt4*^{qmc316} SIVP morphology, endothelial migration dynamics, the relationship between anterior-posterior position and timing of sprout regression, endothelial nuclear morphology, and *vegfaa* and *vegfc* expression. Together, these revisions clarify the relationship between blood flow, endothelial migration, and leading sprout regression, and support a context-specific requirement for Flt4 activity under reduced- flow conditions. We address each point below with our response included in blue text.

Comments from the Reviewers:

Reviewer 1: The manuscript presents thorough phenotypic of SIVP development in zebrafish under differential flow conditions, with a specific focus on remodelling of this vascular bed. The authors report that blood flow instructs directed migration of ECs within the growing sprouts at the leading edge. Timelapse imaging to track proliferation reveals that following cell division at the leading edge (tip cells), one cell will migrate dorsally, against the direction of flow, whilst the other daughter remains at the tip. This makes for an even distribution of the ECs throughout the SIVP. In embryos without flow, directionality, and associated polarised Golgi positioning, is lost and there is an increase in the number of ECs that remain at the tip. Further application of a gata1 morpholino (depleting RBCs) suggests that regression does occur under conditions of reduced flow. To interrogate whether there is a role in VEGF signalling here, adjusting the flow set-point, the authors examined spatio-temporal expression of VEGF receptors (control vs no flow) in the SIVP. flt4 was differentially expressed and remained high in embryos without flow. The functional relevance of flt4 was shown in combination with gata1 morpholino knockdown; whilst both loss-of-function models individually did not lead to regression failure, when combined regression was compromised. This led to the conclusion that flt4 is required to regulate regression under low flow conditions.

The study is suited for publication and is likely to be of interest to the readership of Development. However, I have raised several comments below that I recommend are addressed prior to publication.

1. To model "low flow" the team has applied the previously validated gata1 morpholino. Whilst loss of circulating RBCs would undoubtedly lower shear on the endothelium, there is still fluid flow. Compared with tnnt2a knockdown, gata1 knockdown is therefore a much milder interference which likely explains why regression is not affected. I believe complementary approaches to model "low flow" would strengthen the conclusions. For example, temporal decrease or increase of flow using chemical antagonists/agonists during the time of SIVP rearrangements. Furthermore, the tnn2a morpholino can be titrated down to reduce flow (Vignes at al. Dev Cell 2022).

2. Comments above can be extended to the data presented in Figure 7, where flt4 is shown to be required for regression under low flow conditions. Based on the data presented in 7D, there are still a considerable number of embryos where vessels do regress in double LOF (flt4-/- and gata1 MO) and in only 1/18 embryos are there three sprouts left (as shown in G). To strengthen this data, I therefore strongly suggest the authors apply complementary approaches to alter flow in the flt4 mutants.

We thank the reviewer for their constructive comments and address point 1 and 2 here. We have performed acute blood flow reduction by treating embryos from a *flt4qmc316*/+ incross with 10uM nifedipine between 56-72hpf. This dosage significantly reduced blood flow velocity (Fig. S16A; lines 491-497). We quantified sprout regression blindly in embryos and observed no significant difference in sprout regression between nifedipine and DMSO treated WT embryos from a *flt4*^{qmc316}/+ incross. This is consistent with our previous observations in *gata1a* knockdown

© 2026. Published by The Company of Biologists under the terms of the Creative Commons Attribution License (https://creativecommons.org/licenses/by/4.0/).

experiments (Fig. 7). Furthermore, we observed that nifedipine treated *flt4^{qmc316}* mutant embryos displayed significantly increased numbers of retained leading sprouts compared to DMSO treated *flt4 ^{qmc316}* mutant embryos (Fig. S16). This also supports our observations of failed sprout regression in *gata1a* mo-injected *flt4* mutants. Collectively, this orthogonal experiment supports our proposal that *flt4*-mediated sprout regression depends upon blood flow. In addition, we also abolished flow completely by treating with higher doses of nifedipine from 56hpf-72hpf and this phenocopied *tnnt2a* knockdown (Fig. S3; lines 277-286, and please also see response to reviewer 3 point 1).

3. In gata1 morpholino injected embryos the vessel running along the SIVP plexus appears to have a wider lumen compared to controls (across all figures). Does this impact perfusion across the SIVP network? In a study by Sugden et al (NCB 2022) Engoglin was shown to sense flow and thereby coordinate cell shape changes so that vessel diameters are adjusted appropriately and all vessels in the trunk network are perfused. It would be beneficial if the authors conduct similar timelapse imaging of cellular behaviour (directionality) in the double LOF (flt4-/- and gata1 MO) embryos, as presented for the tnnta morphants (Fig 4-5), in conjunction with a vessel dye to label perfusion of regression.

We agree that some representative images appear to show increased SIV diameter. However, quantitative analysis showed that this phenotype was not consistent across experimental contexts. In otherwise wild-type embryos, *gata1a* morpholino injection increased SIV diameter (Fig. S11), and this was suppressed by co-injection of the *flt4* morpholino (see figure included below). However, in embryos from *flt4^{qmc316}* heterozygous incrosses (Fig 7), we did not detect a significant difference in SIV diameter between control morpholino and *gata1a* morpholino- injected embryos (see below for quantification). Given this inconsistency, we do not consider the SIV diameter phenotype sufficiently robust to support a central conclusion of the manuscript. We have therefore not included these data in the revised manuscript and have limited our conclusions to sprout regression, where the effects are reproducible across genetic, morpholino and pharmacological approaches.

In *gata1a* morphants, erythrocytes labelled by *Tg(gata1a:DsRed)* are observed within SIVP branches, indicating that vessel connectivity and erythrocyte passage are retained despite reduced haematocrit. While this does not exclude subtle changes in flow distribution, it indicates that the SIVP is perfused under these conditions.

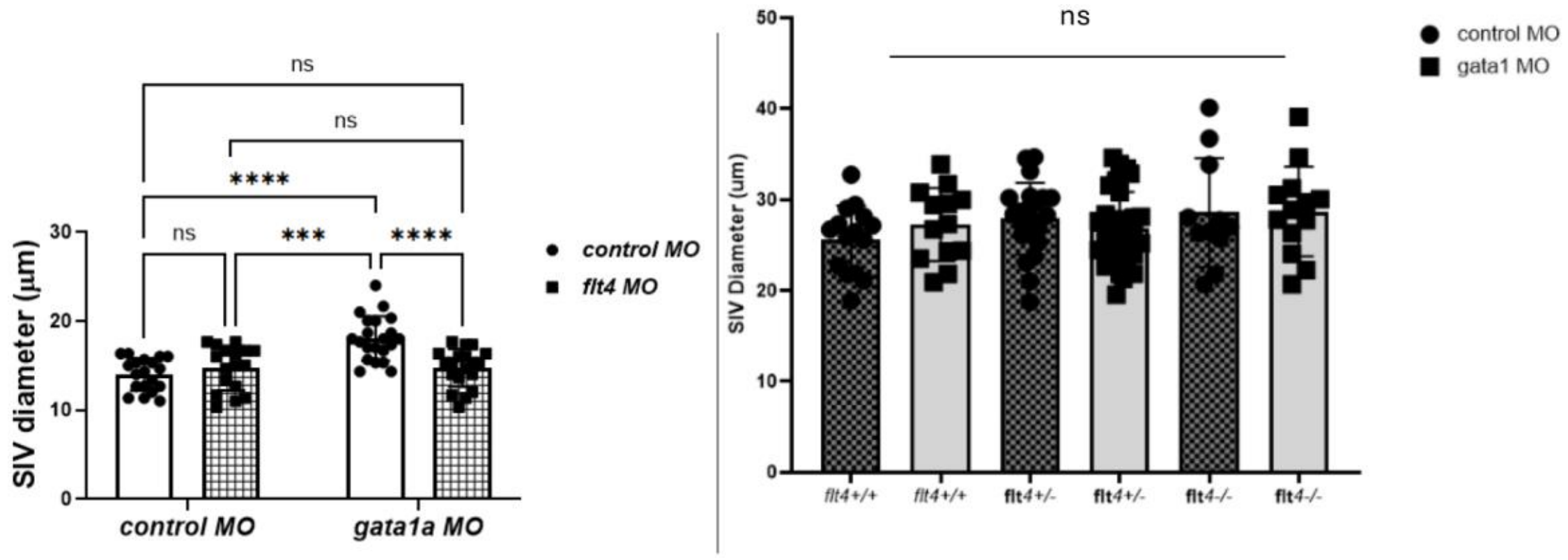

4. Actin labelling in the AC-TagRFP line is not as defined as in other previously developed endothelial actin lines (lifeact or uthrophin). To faithfully monitor individual cells over time it would be worth repeating this data using these available lines.

We agree with the reviewer that LifeAct- and Utrophin-based reporters are well-established tools to visualise filamentous actin. In our hands, however, *Tg(fli1a:AC-TagRFP)* provided more stable intracellular actin labelling during long time-lapse imaging than our endothelial LifeAct reporter, *Tg(fli1a:LifeAct-mClover)*, which preferentially labelled filopodial actin and was more prone to photobleaching under the imaging conditions required for this experiment. Actin chromobody reporters have also been shown to recover rapidly after photobleaching and to capture actin redistribution during dynamic developmental processes (Panza et al, Development, 2015,

© 2026. Published by The Company of Biologists under the terms of the Creative Commons Attribution License (https://creativecommons.org/licenses/by/4.0/).

https://doi.org /10.1242/dev.118943).

To address this point directly, we attempted to repeat the experiment using *Tg(fli1a:LifeAct-mClover)*. We observed actin foci resembling the actin ring-like structure seen before sprout retraction in the AC-TagRFP line; however, photobleaching during long-term imaging reduced image quality and made direct quantitative comparison difficult.

We have therefore retained the AC-TagRFP data but have revised the text to interpret these observations more cautiously (lines 394-402). In addition, our new nuclear morphology analysis shows that reverse-migrating sprout ECs have more rounded nuclei than neighbouring SIV ECs, supporting the conclusion that these cells are not simply passively incorporated into the SIV wall through flow-induced elongation.

5. There are dsRed+ve cells outside the vasculature across multiple figures. To assist readers, I suggest the authors should describe what these cells are in the text and point those out in the figure to clarify that these are not the cells of interest.

We have added a note explaining the occurrence of ectopic *Tg(gata1a:DsRed)*+ cells to the text (lines 251-254).

6. In movies 4 and 5 there are numerous tracks of individual cells which makes it very challenging to follow these cells/tracks over time. A cleaner representation of this data is requested.

We agree that the density of tracks in Movies 4 and 5 makes individual cell trajectories challenging to follow. To improve clarity, we have added two new supplementary figures, Figures S4 and S5, which provide cleaner visualisations of the same tracking data. These figures separate the analysis by cell type, including tip cells and ventral SIV cells, and show paired control and tnnt2a morphant experiments. Figure S4 displays endothelial migration direction over time, while Figure S5 shows individual cell trajectories and clustering behaviour during SIVP remodelling. These additional visualisations complement the movies and make the cell-type-specific migration behaviours easier to assess.

Reviewer 2: SUMMARY OF THE ADVANCE MADE IN THIS PAPER AND ITS POTENTIAL SIGNIFICANCE TO THE FIELD

In this work, Chen et al. examine the development of the subintestinal vessels plexus (SIVP) in the zebrafish as a model for understanding how blood flow impacts endothelial cell (EC) migration and vascular remodeling. While development of this vascular bed from the posterior cardinal vein (PCV) has been extensively described in previous work, this paper focuses primarily on flow-mediated regression of the ventrally-directed "leading sprouts" that seem to pull the plexus over the yolk. The authors conclude that these cells polarize and migrate dorsally in response to blood flow and incorporate into the subintestinal vein (SIV), and that this behavior requires vegfr3/flt4, but only in conditions of low hematocrit/low viscosity/low shear stress. The fluorescent imaging data are nicely presented, and the work adds to our understanding of development of the SIVP. However, primary conclusions are not particularly novel. That is, it is well established that SIVP leading sprouts retract to the SIV, that blood flow induces vascular quiescence, that pruning/sprout retraction primarily occur via polarization and migration toward high flow or parent vessels, and that vegf3/flt4 plays a role in mechanosensing in low flow vessels. Additional analyses could add new mechanistic insight, strengthen conclusions, and increase the impact of this work.

We thank the reviewer for their constructive assessment of our manuscript and for recognising that the imaging data are clearly presented and that the work adds to our understanding of SIVP development. We agree that our study builds on important previous work on SIVP formation, flow-dependent vascular remodelling, endothelial migration during pruning, and VEGFR3/Flt4-dependent mechanosensing. We address the reviewer’s specific comments below.

SUGGESTIONS TO AUTHORS

© 2026. Published by The Company of Biologists under the terms of the Creative Commons Attribution License (https://creativecommons.org/licenses/by/4.0/).

Major comments

1. The authors state that SIVP perfusion occurs in an anterior-to-posterior fashion, and that blood flow triggers leading sprout regression. Accordingly, it would be informative to investigate whether there is a spatiotemporal correlation between blood flow and leading sprout regression.

To assess whether leading sprout regression correlates spatially with the reported anterior-to-posterior onset of SIVP perfusion, we quantified leading sprout persistence according to each sprout's relative anterior-posterior position within the SIVP. Surprisingly, this analysis revealed that posterior leading sprouts regressed earlier than anterior leading sprouts, indicating a posterior bias in sprout regression. We have added these data as a new supplementary figure, (Figure S1; lines 256-259). This finding is unexpected, given that posterior plexus segments are perfused later, and suggests that leading sprout regression is not simply explained by the timing of local perfusion.

2. Flow through the SIV causes EC elongation along the flow axis (based on nuclear morphology), and this change in shape and orientation might be sufficient to pull the sprout cells into the SIV wall. How can you distinguish between this pulling versus active migration of these cells, less than 50% of which are dorsal-polarized at 64 hpf? It would be informative correlate dorsal polarization with dorsal migration at the individual cell level, and/or to analyze EC shape changes in the SIV over time using the actin nanobody line.

To address whether leading sprout regression reflects passive incorporation into the SIV wall due to flow-induced endothelial elongation, rather than active dorsal migration, we quantified endothelial nuclear morphology in the SIV and in cells undergoing dorsal sprout regression. Specifically, we compared nuclear eccentricity between SIV endothelial cells and dorsally migrating sprout cells. Consistent with the reviewer's premise, SIV endothelial cells displayed elongated nuclei, in line with the well-established response of endothelial cells to shear stress, in which cells elongate and align with the direction of flow (Lenard *et al.,* 2015 PMID 25884426; Franco *et al.,* 2015 PMID 25884288).

By contrast, dorsally migrating sprout cells had significantly lower nuclear eccentricity and were therefore more rounded than SIV endothelial cells. The more rounded nuclear morphology of dorsally migrating sprout cells, compared with elongated SIV endothelial cells, argues against their passive incorporation through flow-induced elongation and is more consistent with active endothelial rearrangement/migration. Together with their dorsal displacement over time, these data support the interpretation that leading sprout regression involves active endothelial cell rearrangement/migration rather than passive incorporation into the SIV wall. We have added this analysis to the revised manuscript (Fig. S6U; lines 394-402).

3. In Figure 5, the step data plotted in panels H/I/K/L would be better analyzed by radial statistics. Binning these movements into dorsal, ventral, anterior, or posterior steps fails to fully represent the data. Notably, the graphs in J and M look remarkably similar, whereas the plots in H/I and K/M do not. In panel I, I see a confinement of migration along the DV axis, with less movement along the AP axis, but this isn't clear from the graph in panel J. Comparing panels K and L, I see very little change in tnnt2a MO versus control. I cannot appreciate that control cells display a tendency toward posterior migration while tnnt2a MO show significant anterior migration, as stated in the text.

We thank the reviewer for this helpful suggestion. We agree that the previous binning of migration steps into broad dorsal, ventral, anterior and posterior categories did not fully capture the directional information in the tracking data. We have therefore revised the Figure 5 analysis and now present the migration-step data using rose plots, which better represent the distribution and magnitude of endothelial cell movements in different directions. Representative rose plots are included in the revised main figure (Fig. 5H,I), with the full analysis across paired experiments shown in the new supplementary Figure S4.

In addition, we have revised the associated quantification to focus on reverse migration during

© 2026. Published by The Company of Biologists under the terms of the Creative Commons Attribution License (https://creativecommons.org/licenses/by/4.0/).

sprout regression. This analysis shows that reverse migration is significantly reduced in *tnnt2a* morphants compared with controls (Fig. 5J). We also added a nearest-neighbour analysis showing that endothelial cells in *tnnt2a* morphants remain more closely clustered than in controls (Fig. 5K-M; Fig. S5), consistent with impaired redistribution of cells during sprout regression. We have revised the text accordingly to avoid overinterpreting anterior/posterior directional biases and to more directly reflect the revised analyses (lines 360-372).

4. Data presented do not support the authors' conclusions as stated in lines 328-336. In Figure 5, the temporal resolution is lost in data panels H-M. In panel N, where temporal resolution is preserved, only 3 cells are tracked over time for control and tnnt2a morphants. Accordingly, it is unclear how the authors can conclude from these data that in controls, there are differences in migratory behavior before and after flow. And in tnnt2a morphants, the authors state that SIV ECs migrate anteriorly, but panel N shows that the migration is mostly ventral and skews posteriorly, assuming that the axes are the same as in H/I/K/L.

We recognise that the original presentation of Figure 5 made it difficult to evaluate the temporal dynamics of endothelial cell migration. In particular, temporal information was not readily apparent in the summary plots, and the schematic in the original panel N may have given the impression that it was derived from a small number of representative cell tracks rather than the full tracking dataset.

To address this, we have performed an extensive re-analysis of the endothelial cell tracking data and have substantially revised the data presentation. In the revised Figure 5, we now show migration steps over defined time intervals, preserving temporal information and allowing directional changes in endothelial cell movement to be assessed over the course of SIVP remodelling. We have also added new supplementary figures, Figures S4 and S5, showing the full extent of endothelial cell migration over time in individual embryos. These visualisations allow the reader to assess embryo-to-embryo variability and the temporal progression of migration directly.

In response to the reviewer's concern, we have revised this section (lines 360-372) to more explicitly link the conclusions to the corresponding quantitative analyses. The revised text now distinguishes between the measured directional biases in endothelial migration and the model we propose to explain how blood flow coordinates these behaviours. Specifically, our revised analysis supports the conclusion that, in the absence of flow, tip cells show increased ventral migration associated with failure of sprout regression, whereas in controls, tip cell movement is biased dorsally and sprout regression proceeds. For SIV endothelial cells, we now describe the observed directional bias in terms of the quantitative migration-step analysis and have clarified the axes used in all panels to avoid ambiguity.

This re-analysis supports our overall conclusion that blood flow influences the directionality of endothelial cell rearrangements during SIVP remodelling, while avoiding overinterpretation of the schematic representation.

5. In situ hybridization data (Figures S4, S6) are not publication quality. It is very difficult to see the SIVP due to very high background staining. Also, the images lack scale bars.

We agree that the presentation of the in situ hybridisation data makes the SIVP signal difficult to assess. We have repeated and attempted to optimise the staining, including bleaching/clearing steps, however, since these transcripts are expressed at relatively low levels in the SIVP, extended staining was always required to visualise signal in this region, which increased background staining. We have added scale bars and also revised the figure legends and text (line 429-431) to ensure that these data are interpreted appropriately.

6. In the flt4 mutants + gata1 morpholino experiments, it would be helpful to assess SIV EC elongation and alignment against flow to help to differentiate between defective sprout EC dorsal "migration" versus defective "pulling" of sprout ECs to the SIV wall (see point 2 above)

© 2026. Published by The Company of Biologists under the terms of the Creative Commons Attribution License (https://creativecommons.org/licenses/by/4.0/).

We agree that assessing SIV endothelial elongation/alignment in the *flt4* mutant + *gata1a* morpholino background would provide an additional test of whether sprout regression reflects active migration or altered incorporation into the SIV wall. During revision, we prioritised analyses that could be completed rigorously within the available timeframe, including re-analysis of endothelial migration dynamics and quantification of endothelial nuclear eccentricity. Performing the proposed experiment in the *flt4* mutant low-flow background would require an additional experimental series and was therefore beyond the scope of the revision.

Importantly, our new nuclear eccentricity analysis addresses the same underlying concern. Regressing sprout cells displayed lower nuclear eccentricity than SIV endothelial cells, arguing against a simple passive-pulling model.

Minor comments

1. The description of SIVP development could be improved. For example, line 66 states that the ECs from the primary SIV migrate dorsally to form branches and the SIA. But from published work (e.g. Hen et al. Development 2015) as well as provided movies, it is clear that some cells get "dropped off" during ventral migration from the PCV; these cells may move dorsally into the SIA.

We thank the reviewer for this clarification. We have revised the description of SIVP development to better reflect previous work, including the contribution of cells that migrate from the PCV, are retained within the developing plexus, and subsequently contribute to dorsal SIVP structures including the SIA. We have modified the text on lines 66-69.

2. Line 77 typo Zzebrafish

Agreed

3. Line 97 change lumen to SIV

We have retained “lumen” at this point because the sentence refers to incorporation of sprout cells into the patent vessel lumen during regression, rather than specifically to their final position within the SIV. To avoid ambiguity, we have revised the wording to “incorporate into the lumen of the SIV.”

4. Line 117 construct synthesis is not in silico synthesis; maybe silicon-based DNA synthesis?

- We have removed this

5. Line 171 sentence fragment

Agreed

6. Movie 2 please annotate fusion event differently from sprout elongation and add timeframe to legend.

We have added the imaging timeframe to the Movie 2 legend. Because Movie 2 is intended to show the overall dynamics of leading sprout behaviour during SIVP remodelling, we have not added further event-specific annotations to the movie itself. Sprout elongation and fusion/anastomosis events are now described in the legend.

7. Figure 3 there is no reference to panels D-F in the text.

© 2026. Published by The Company of Biologists under the terms of the Creative Commons Attribution License (https://creativecommons.org/licenses/by/4.0/).

Agreed

8. Line 222 typo: hereafterleading

Agreed

9. In movies 4-6 that support Figure 4, it would be helpful to annotate at least some examples of ECs that move from one vessel segment to another as quantified in Figure 4D.

We thank the reviewer for this suggestion. We have retained Movies 4–6 with minimal annotation so that the underlying endothelial cell dynamics remain visible throughout the time-lapse. The EC transitions between vessel segments are quantified in Figure 4D and Supplementary Tables 3 and 4, and we have revised the text to more clearly link these movies to the quantified cell- transition analysis (lines 297-303). This allows the movies to show the raw cell behaviours while the figure and tables provide the corresponding quantification.

10. Figure 4D is confusing to me because it shows number of cell movements and the text discusses percent of cells moving, so it was hard to reconcile the figure and text.

We have revised the text to remove potentially confusing percentage descriptions and added percentages to Supplementary Table 3 and 4.

11. In Figure 4 legend, remove the word "(magenta)" on line 786.

Agreed

12. Movie 6: define the arrow in the legend as pointing to the dorsal daughter cell

Agreed

13. Figure 5 define quadrants for polarization analysis in the legend and perhaps color code the datapoints in H,I,K,L to reflect A/P/D/V designations. In the legend, indicate timing of experiment (56-72 hpf?)

The Figure 5 analysis and presentation have been substantially revised. The previous quadrant-based presentation is no longer used, and the revised figure now defines the axes and timing of the migration analysis directly in the figure and legend.

14. Line 347: It is not true that most tip cells showed ventral Golgi positioning at 56 hpf; this is the largest bin but it is less than 50%. Please rephrase.

We have now removed this data (see comment to point 17).

15. Figure S7E: please label the X-axis to clearly define each experimental group. Consider a different statistical test for interaction.

We thank the reviewer for this suggestion. We have clarified the statistical analysis in the figure legend. Because this experiment tests the effects of two variables, *gata1a* knockdown and *flt4* perturbation, and whether these variables interact, we analysed the data using two -way ANOVA with an interaction term. We have now explicitly reported the interaction analysis in the figure legend and added clearer group labels/statistical annotations to Figure S7E.

© 2026. Published by The Company of Biologists under the terms of the Creative Commons Attribution License (https://creativecommons.org/licenses/by/4.0/).

16. Figure S2B diagram: replace flow with SIV and migration with sprout.

We have revised the Figure S2B legend to clarify that “flow” refers to blood flow within the SIV and that “migration” refers to leading sprout cell movement along the sprout axis. We retained the labels in the schematic because they indicate the biological directions used for the polarity analysis rather than naming the anatomical structures themselves.

17. Figure S2C,D analysis requires categorical variable test like Fisher's exact

We agree that the data in Figure S2C, D are categorical and should be analysed using Fisher’s exact test. Following re-analysis, some comparisons were no longer statistically significant. Because this analysis was supportive rather than central to the manuscript, we have removed the Golgi polarity data from the revised version and revised the text to avoid conclusions based on this dataset. Our conclusions now rely on the live-imaging, migration-step, nearest-neighbour, nuclear morphology and actin analyses.

18. In Figure 7, the flt4 mutant SIVP seems to have abnormal morphology. Is this typical? Can the authors comment?

We have added analysis of SIVP morphology in *flt4*^{qmc316} incross embryos at 56 and 72 hpf (Fig. S14). Across wild-type, heterozygous and homozygous siblings, we did not detect significant differences in SIVP patterning under normal-flow conditions. The morphology shown in Figure 7 is therefore within the range of variation observed in sibling controls and is not representative of a consistent genotype-associated SIVP defect.

19. Line 492 sentence fragment

Agreed

Reviewer 3: SUMMARY OF THE ADVANCE MADE IN THIS PAPER AND ITS POTENTIAL SIGNIFICANCE TO THE FIELD

In the manuscript by Chen et al, the authors use the subintestinal vessels (SIV) in zebrafish to investigate the role of blood flow in regulating vascular plexus formation. In particular, the authors find that regression of leading angiogenic sprouts at the vascular front of the SIV plexus is regulated by blood flow - under normal conditions, upon blood flow into SIVs, leading sprouts collapse and migrate back into the patent SIV. However, in the absence of flow (but not red blood cells) these sprouts contain more cells than normal and fail to migrate back into the main SIV. Interestingly, the authors find that flow-dependent regression is independent of apoptosis or changes in cell proliferation. They further note that reduction of VEGFC receptor (Vegfr3) limits regression, but only when red blood cell volume is simultaneously reduced. In general, these are interesting findings that modestly add to the literature on vascular regression - a fairly detailed analysis of SIV morphogenesis has been provided by several previous papers, though more detailed analysis on the role of flow is provided here. Further, there are several issues that may limit the potential impacts of these findings, especially regarding the genetic interaction between flt4 and flow. Specific comments are listed below.

SUGGESTIONS TO AUTHORS

Major comments/issues

1. All of the manipulations here involve blocking gene function from beginning of development, while phenotypic assessment is not until much later. Since SIV formation and plexus remodeling is a relatively late event (past 2 dpf), it is not possible to rule out that the observed phenotypes are simply downstream effects. While it is challenging to acutely regulate RBC volume, there are

© 2026. Published by The Company of Biologists under the terms of the Creative Commons Attribution License (https://creativecommons.org/licenses/by/4.0/).

several ways to regulate flow at later time points - cardiac ablation (e.g. via laser), "cauterization" (also via laser) of the SIV to block flow, or treatment with high dose tricaine. The authors need to incorporate any of these to verify that their results are due to flow acting at the stage they are proposing, at least regarding regression (I would not recommend using BDM to stop the heart as that can also affect pre-flow angiogenesis). Flt4 can be similarly inhibited via a selective small molecule inhibitor at the appropriate dose (MAZ51 or similar) and stage.

We thank the reviewer for this important suggestion. To determine whether blood flow is required specifically during the sprout regression phase, we inhibited cardiac contraction pharmacologically from 56–72 hpf, the period during which leading sprout regression normally occurs. Treatment with 20 μM nifedipine blocked SIVP flow during this window and phenocopied the increased leading sprout retention observed after *tnnt2a* knockdown (Fig. S3), supporting a temporally specific requirement for flow during sprout regression (lines 277-286).

We next used 10 μM nifedipine to reduce, but not fully ablate, flow during the same period. This was sufficient to induce leading sprout retention in *flt4*^{qmc316} mutants (Fig. S16, lines 490-495). In parallel, we inhibited Flt4 activity pharmacologically by treating *gata1a* morphants with MAZ51 from 56–72 hpf, which also caused leading sprout retention when both *gata1a* morpholino and MAZ51 were used in combination but not individually (Fig. S15, lines 482-488). Together, these data support a temporal requirement for blood flow during sprout regression and indicate that Flt4 activity is required under reduced-flow conditions.

2. Lines 82-83, 392-393. The Baeyens et al. paper is a bit over-interpreted. The effect in zebrafish is only on the dorsal aorta (which actually lacks flt4 expression), while the artery remodeling effect is in mouse. Further, definitive genetic data (e.g. alleles that specifically block the proposed mechanosensory role of Vegfr3) are lacking - all of the zebrafish studies in that paper were using morphants including "high dose" experiments. Finally, the "shear stress setpoint" was determined based on flow chamber studies in vitro without any RBCs.

We thank the reviewer for this comment and have adjusted text in the manuscript accordingly

3. Line 227, 377. The term "perfusion" generally refers to any constituent of the blood, including red blood cells (RBCs). The leading sprouts appear to be lumenized and may be in contact with other blood constituents. This is probably worth checking (via injection with a low molecular weight fluorescence tracer - e.g. QDots) to determine whether these sprouts are or are not "perfused" in some way.

We agree that it is not clear whether leading sprouts are perfused and that the presence of RBCs does not account for other flow constituents. We attempted to address this concern using a transgenic reporter line, *Tg(lfabp:DBP-EGFP)*, which drives expression of a fluorescently labelled blood serum protein as a proxy for blood flow. We were unable to clearly discern whether flow was found within sprouts. We have therefore clarified these as perfusion by erythrocytes and have added a comment to note that we cannot exclude the presence of flow within the SIVP prior to erythrocyte entry at lines 259-260.

4. Line 387. ". . under low flow conditions. . .". This is a bit confusing and/or vague. Gata1a morphants have "flow" - they just have reduced RBC volume and everything else in circulation should largely be the same. Underscoring this fact is that the severe depletion of RBCs did not have a significant effect on SIV sprouts, suggesting some other flow component is playing a role. This "blooid flow" concept is vague in other places as well and should probably be more precisely defined.

We have replaced “low flow” with “reduced-viscosity flow” throughout the manuscript.

5. I did not see any movies of the tnnt2a morphants. These should be included.

© 2026. Published by The Company of Biologists under the terms of the Creative Commons Attribution License (https://creativecommons.org/licenses/by/4.0/).

Movies 3 and 5 show SIVP development in *tnnt2a* morphants.

6. The epistasis between flt4 and gata1a is potentially interesting. However, there are a number of issues here: 1. The flt4 mutant may not be a null. Several different flt4 alleles have been reported that all have edema by 5 dpf - the mutant described here does not, suggesting it may be hypomorphic, possibly due to compensation or exon skipping. There are in-frame deletion alleles available that specifically target Vegfc binding that would be more informative, as would confirmed null alleles. 2. Similarly, gata1a knockdown is also hypomorphic, confounding any interpretation of the apparent epistasis. 3. Lacking genetic analysis of Vegfc. This is important since the flt4 allele here would be expected to affect Vegfc-dependent signaling. Would also be helpful to know pattern of vegfc in normal and gata1a-deficient embryos.

We thank the reviewer for these helpful comments. We agree that interpretation of the interaction between *flt4* and reduced erythrocyte flow requires caution, particularly because our *flt4* allele has not been definitively established as a molecular null and because *gata1a* knockdown produces a partial reduction in erythrocyte flow. We have therefore revised the text to avoid overinterpreting these data as formal genetic epistasis, and now describe them as supporting a requirement for Flt4 activity during sprout regression under reduced-flow conditions.

1) Regarding the *flt4* allele, *flt4*^{qmc316} is predicted to cause an early frameshift and premature truncation in the first extracellular Ig-like domain. Consistent with impaired Flt4 function, mutants show lymphatic development defects comparable to those reported for other *flt4* mutants including reported null alleles. However, because these mutants do not develop overt oedema by 5 dpf, we agree that residual function, compensation, or altered transcript processing cannot be excluded. We have added this caveat to the discussion. At present, UK import restrictions prevent us from obtaining additional published alleles, and generating and validating further alleles would be beyond the scope of this revision.

2) To reduce reliance on gata1a morpholino-mediated erythrocyte depletion, we performed an additional flow-reduction experiment using nifedipine. Reduced flow was sufficient to induce leading sprout retention in *flt4*^{qmc316} mutants, supporting the conclusion that Flt4 is required under reduced-flow conditions independently of the gata1a morphant background (Fig. S16).

3) We also agree that analysis of *vegfc* is important since we could not exclude a potential role for circulating Vegfc during sprout regression. In the absence of available *vegfc* mutants, we performed mosaic G0 CRISPR mutation of *vegfc*. As expected, *vegfc* crispants displayed severe reductions in lymphatic vessels, consistent with effective inhibition of *vegfc* function. However, despite this validated lymphatic phenotype, *vegfc* crispants did not show defective leading sprout regression in the SIVP (Fig. S17). Although the lymphatic phenotype supports effective disruption of *vegfc*, we cannot exclude the possibility that residual *vegfc* activity remains in crispants. We also analysed *vegfc* expression in control and *gata1a* morphants at 56 hpf and 72 hpf, corresponding to the onset of SIVP flow and the regression analysis timepoint, and did not observe an obvious change in expression pattern. These data indicate that the sprout regression phenotype is not simply explained by loss of canonical Vegfc-dependent signalling. These data are described in lines 497-502.

Together, these data support a context-specific requirement for Flt4 activity in sprout regression under reduced-flow conditions, while indicating that overt loss of *vegfc* does not phenocopy this aspect of the *flt4* reduced-flow phenotype. We have revised the discussion to reflect these limitations and to distinguish between Flt4-dependent effects and canonical Vegfc-Flt4 signalling.

Are there any changes in vegfaa/b expression adjacent to SIVs in the absence of flow or RBCs?

There is no observable change in vegfaa expression in the absence of blood flow. We have included data to show this (Fig. S10, lines 448-450).

7. More comprehensive analysis of flt4 mutant SIVs is needed (similar to the analysis done for control vs. tnnt2 MO) to confirm that there are not earlier SIV defects, or subtle defects.

© 2026. Published by The Company of Biologists under the terms of the Creative Commons Attribution License (https://creativecommons.org/licenses/by/4.0/).

We have now performed a morphological analysis of SIVP development in embryos from *flt4*^{qmc316} heterozygous incrosses at both 56 hpf and 72 hpf, using parameters comparable to those used for the control versus *tnnt2a* morphant analysis. We found no significant differences in SIVP patterning between wild-type, heterozygous and homozygous siblings at either timepoint (Fig. S14, lines 472-480). These data indicate that *flt4*^{qmc316} mutants do not display detectable early or subtle SIVP defects under normal-flow conditions, supporting the interpretation that sprout retention under reduced-flow conditions is not secondary to pre-existing SIVP abnormalities.

Minor comments

8. Lines 53-55. There are a number of context-dependent examples of vascular regression and associated pathways that are regulated in those cases. It clearly is different across blood vessel types, but it is not a complete black box in many cases.

We have adjusted this sentence to reflect the reviewer's suggestion.

9. In some cases, there appear to be gaps in the SIVs (e.g. Figure 7). I assume these are due to pigment cells? How were these accounted for in the context of quantification? Why are they only apparent in some images? Were embryos usually treated with PTU? If so, this should be noted.

We thank the reviewer for highlighting this. The apparent gaps in the SIV are caused by pigment cells obscuring the endothelial fluorescence, particularly in maximum-intensity projections from confocal image stacks. Where possible, we used embryos in a non-pigmented nacre background for quantification. For lines that were not available in a nacre background, quantification was performed using fluorescent stereomicroscope images in which the SIV could be assessed directly, before acquisition of higher-resolution confocal images for representative presentation. Thus, pigment-related obscuration in confocal projections did not affect the quantification. We have clarified this in the relevant figure legends. We did not use PTU for depigmentation.

Second decision letter

MS ID#: dev.205424R1

MS TITLE: Blood flow coordinates collective endothelial cell migration during vascular plexus formation and promotes angiogenic sprout regression via vegfr3/flt4

AUTHORS: Yan Chen; Aaron Michael Savage; Zhen Jiang; Ignacio Rodriguez-Pastrana; Myles Wilson; Hyejeong R. Kim; Paul C. Evans; Robert N. Wilkinson

Dear Dr Wilkinson,

I have now received all the referees reports on the above manuscript, and have reached a decision. The referees' comments are appended below.

The overall evaluation is positive and we would like to publish a revised manuscript in Development, provided that the referees' comments can be satisfactorily addressed. Please attend to all of the reviewers' comments in your revised manuscript and detail them in your point-by-point response. If you do not agree with any of their criticisms or suggestions explain clearly why this is so. If it would be helpful, you are welcome to contact us to discuss your revision in greater detail. Please send us a point-by-point response indicating your plans for addressing the referees' comments, and we will look over this and provide further guidance.

© 2026. Published by The Company of Biologists under the terms of the Creative Commons Attribution License (https://creativecommons.org/licenses/by/4.0/).

Reviewer 1

After assessing the revised manuscript, most concerns raised are addressed with suitable experimentation and data analysis. However, I am not entirely supportive of conclusions drawn around flow when using nifedipine, and on the specificity of the actin foci.

Pharmacological flow manipulation:

As a requested complementary approach to alter flow in both WT and flt4 mutants, the authors have applied nifedipine. Indeed nifedipine (an L-type calcium channel blocker) is frequently used to modify cardiac contractility and as a resultant blood flow/ shear, however, the choice of this compound to examine EC migration/regression for this study is not well justified.
As indicated by the authors in lines 284-285, there are likely "additional effects of calcium channel inhibition". Blocking calcium signaling in endothelial cells can alter cellular processes such as polarization, actin dynamics, and junction remodeling. Particularly in the event described here, where regression is an active process in response to flow, interfering with the ECs ability to be "active" is not ideal. This pharmacological approach therefore is not truly complementary to the tnnt2a knockdown.

Why is the dose of Nifedipine different in S3F (50uM)?

To model reduced flow (10uM) is used (Figure S16). In FigS16E (flt4 mut + low dose nifedipine), the morphology of the entire SIV plexus is abnormal and judging by gata1 expression, it is not perfused at all. Phenotype appears strong for what should be a milder condition, and the image suggests there is no flow. It does not appear to be in accordance with the conclusions drawn from reduced flow conditions.

More specific approaches that are frequently used to alter flow are:

- High dose of the commonly applied anesthetic MS222/Tricaine
- Low dose tnnt2a MO
- Physical obstruction/ablation of vessels

F-actin:

The authors indicate imaging of endothelial lifeact also revealed similar "actin foci". Unfortunately, these are not shown due to bleaching during tracking. Upon re-examination of the images in FigS6, these actin foci appear to be located around nuclei (both within regressing sprouts and within the SIV vessel) and therefore might not be solely a phenomena related to migration. In combination with the data on nuclear shape, however, a cautious suggestion can be made that this is indeed an active process.

Reviewer 2

SUMMARY OF THE ADVANCE MADE IN THIS PAPER AND ITS POTENTIAL SIGNIFICANCE TO THE FIELD

This work exploits the development of the subintestinal vessels plexus (SIVP) in the zebrafish as a model for understanding how blood flow impacts endothelial cell (EC) migration and vascular remodeling. It focuses primarily on flow-mediated regression of the ventrally-directed "leading sprouts." The authors conclude that these cells migrate dorsally in response to blood flow and incorporate into the subintestinal vein (SIV), and that this behavior requires vegfr3/flt4, but only in conditions of low hematocrit/low viscosity/low shear stress. The work adds modestly to our understanding of flow-associated vascular remodeling.

The authors have satisfactorily addressed most of my concerns with the initial submission, and have added a substantial number of figures in response to all reviews. However, a concern remains regarding presentation of migration data.

SUGGESTIONS TO AUTHORS

© 2026. Published by The Company of Biologists under the terms of the Creative Commons Attribution License (https://creativecommons.org/licenses/by/4.0/).

Major comments

1. I appreciate that the authors now present rose plots for cell migration data (Fig 5, new Fig S4), but I am still struggling to interpret the data. The authors say that the plots represent the "coordinates and relative extent of tip and SIVP migration" (as stated in the text) or "magnitude of endothelial cell movements" (as stated in the rebuttal). But I think that the units of the concentric rings must be number of migration steps within the angle bin and not distance traveled. Please explicitly define the units in text and legends. Also, based on the presented data and text and assuming that the time frame of the experiment was 56-72 hpf, I expected to see more migration steps in the dorsal direction (assumed to be centered on 90 degrees), at least in the control tip cells, but I see almost none. Relatedly, panel J shows "increased reverse migration" in control (tip and SIV); I am not sure whether this means migration against flow or dorsal migration, which would be the same thing for tip but not SIVP cells. I am struggling to understand how the rose plots can show almost no dorsal migration yet the reverse fraction graph depicts means of over 85% in all groups. As suggested by panels K and L, if all tracked cells are moving ventrally (likely because of large scale morphogenetic movements during embryo development), then you won't fully capture dorsal migration in this analysis.

Minor comments

1. Former point 3, current lines 98-99: The lumen is the interior of the vessel that is bound by but exclusive of the endothelium. Perhaps change from "lumen of the SIV" to "lumenized SIV."
2. Figure 5 the legend still does not indicate timing of experiment
3. Figure S6 and movie 7 track nuclei in a cytoplasmic line, fli1a:EGFP. How well can nuclei be distinguished in this line? Some justification is required for the use of this line versus a nuclear line.
4. Figures S15,S16: Why can we only see individual data points in the rightmost column in S15J and S16F? Perhaps decrease point sizes. Additionally, all data should be analyzed by 2-way ANOVA (treatment + genotype).
5. The value of Figure S17 (vegfc crispants) is unclear. I realize that it was added in response to reviewer comments, but there are no data under altered flow conditions.

<u>Reviewer 3</u>

SUMMARY OF THE ADVANCE MADE IN THIS PAPER AND ITS POTENTIAL SIGNIFICANCE TO THE FIELD

In their revised manuscript, Chen et al. have addressed most of my comments. However, there remain several related issues that still need to be cleared up.

SUGGESTIONS TO AUTHORS

Line 247. "Circulation progressed from anterior to posterior. . ." This is a bit confusing since I believe (and according to schematics in 1C'-F') flow itself ends up going the opposite direction. I think a more accurate description would be the "onset of circulation (or perfusion?)" progresses from anterior to posterior.

Lines 452-458. I believe that the Flt4 mutant is used for all of the analysis, so the MO data could probably be left out.

Lines 463-467. Based on comparison to the cited literature (Hogan, Shin), the qmc316 allele is likely to be a hypomorph, as it does not result in lymphedema, which is clearly observed with null alleles. This should be explicitly stated.

Line 464. The presence of lymphedema in flt4 mutants has been correlated to the presence/absence of the main facial lymphatic vessel and valve, which looks like it is intact in the qmc316 mutant. This is not well-described here - the figure legend is vague. Zoomed-in views of thoracic duct and facial lymphatic network, and more explicit labeling, would be helpful here.

© 2026. Published by The Company of Biologists under the terms of the Creative Commons Attribution License (https://creativecommons.org/licenses/by/4.0/).

Line 471. Please label the gaps caused by pigment cells with an asterisk. Also, there does not seem to be any mention of using the nacre mutant, though that was included in the author rebuttal. Please include the use of nacre in the methods section.

Line 483-484. Similar issue as above for characterization of lymphatic defects in MAZ51-treated embryos. Authors need to provide a better analysis here (zoomed in views, labeling particular lymphatic structures). Also, it seems the MAZ51 dose is a bit high - the density of the brain blood vessels labeled by kdrl:mcherry is clearly reduced, suggesting it may be blocking inhibiting Vegfr-2-related receptors. The authors need to use a dose that inhibits thoracic duct formation, without affecting blood vessels.

Lines 499-500. It is possible that Flt4 is acting in a ligand independent manner in the context of a "set point" - this would be consistent with VEGFR2 acting in a ligand-independent manner with PECAM and CDH5 (as cited by the authors) as a hemodynamic sensor. Therefore, it is important that the Vegfc F0 CRISPR experiment mimic a null. Onset of lymphedema in vegfc and flt4 mutants occurs after 72 hpf, so this should not be a concern. The functional level of knockout is not clear from the data presented here. It is important to assess the SIV network using a Vegfc CRISPR dose that gives nearly fully penetrant lymphedema.

Second revision

Author response to reviewers' comments

Comments from the Reviewers:

Reviewer 1: After assessing the revised manuscript, most concerns raised are addressed with suitable experimentation and data analysis. However, I am not entirely supportive of conclusions drawn around flow when using nifedipine, and on the specificity of the actin foci.

Pharmacological flow manipulation:

As a requested complementary approach to alter flow in both WT and flt4 mutants, the authors have applied nifedipine. Indeed nifedipine (an L-type calcium channel blocker) is frequently used to modify cardiac contractility and as a resultant blood flow/ shear, however, the choice of this compound to examine EC migration/regression for this study is not well justified.

As indicated by the authors in lines 284-285, there are likely "additional effects of calcium channel inhibition". Blocking calcium signaling in endothelial cells can alter cellular processes such as polarization, actin dynamics, and junction remodeling. Particularly in the event described here, where regression is an active process in response to flow, interfering with the ECs ability to be "active" is not ideal. This pharmacological approach therefore is not truly complementary to the tnnt2a knockdown.

We thank the reviewer for this important point. We agree that that calcium-channel inhibition may have effects on endothelial cell behaviour, including cytoskeletal organisation, polarity and junctional remodelling. We have therefore revised the manuscript to avoid presenting nifedipine as a clean or fully complementary flow-only perturbation. Instead, we now describe nifedipine as an acute pharmacological inhibition of cardiac contraction/circulation, used to test whether perturbing circulation during the sprout-regression window affects SIVP remodelling (lines 303-306).

Why is the dose of Nifedipine different in S3F (50uM)?

The 50 μM label in Fig. S3F was a labelling error and has now been corrected. Nifedipine was used at 20 μM for the acute flow-blocking experiments shown in Fig. S3.

To model reduced flow (10uM) is used (Figure S16). In FigS16E (flt4 mut + low dose nifedipine), the morphology of the entire SIV plexus is abnormal and judging by gata1 expression, it is not perfused

© 2026. Published by The Company of Biologists under the terms of the Creative Commons Attribution License (https://creativecommons.org/licenses/by/4.0/).

at all. Phenotype appears strong for what should be a milder condition, and the image suggests there is no flow. It does not appear to be in accordance with the conclusions drawn from reduced flow conditions.

More specific approaches that are frequently used to alter flow are:

- High dose of the commonly applied anesthetic MS222/Tricaine
- Low dose tnnt2a MO
- Physical obstruction/ablation of vessels

For the reduced-flow experiments in Fig. S16, all embryos were selected for detectable SIVP flow prior to imaging. We agree that the representative image in the previous version could give the impression of complete loss of perfusion, likely because flow was further reduced during mounting and tricaine anaesthesia. To address this concern, we have revised the figure and added an independent reduced-flow experiment using 2× tricaine. This treatment reduced blood flow without preventing SIVP formation or causing overt vascular collapse, and produced results comparable to the 10 μM nifedipine condition. These data have now been added to Fig. S16 and are described in the manuscript on lines 530–533.

We have also revised the text to make clear that pharmacological flow-reduction experiments are used as supportive evidence alongside *tnnt2a* loss-of-function and reduced-viscosity/*gata1a* experiments (lines 521-523).

F-actin:

The authors indicate imaging of endothelial lifeact also revealed similar "actin foci". Unfortunately, these are not shown due to bleaching during tracking. Upon re-examination of the images in FigS6, these actin foci appear to be located around nuclei (both within regressing sprouts and within the SIV vessel) and therefore might not be solely a phenomena related to migration. In combination with the data on nuclear shape, however, a cautious suggestion can be made that this is indeed an active process.

We thank the reviewer for this helpful comment. We agree that the actin foci/ring-like structures should not be interpreted as specific markers of migratory sprout cells, particularly as similar perinuclear actin enrichment can also be observed in other endothelial cells within the SIV. We have therefore revised the manuscript to describe these structures more cautiously as evidence of cytoskeletal remodelling associated with sprout regression, rather than as sprout-specific actin foci or definitive evidence of migration. In combination with the nuclear morphology analysis and live tracking data, we now interpret these observations as supportive of an active endothelial rearrangement process during sprout regression. We have adjusted the text accordingly (line 427-428).

Reviewer 2: SUMMARY OF THE ADVANCE MADE IN THIS PAPER AND ITS POTENTIAL SIGNIFICANCE TO THE FIELD

This work exploits the development of the subintestinal vessels plexus (SIVP) in the zebrafish as a model for understanding how blood flow impacts endothelial cell (EC) migration and vascular remodeling. It focuses primarily on flow-mediated regression of the ventrally-directed "leading sprouts." The authors conclude that these cells migrate dorsally in response to blood flow and incorporate into the subintestinal vein (SIV), and that this behavior requires vegfr3/flt4, but only in conditions of low hematocrit/low viscosity/low shear stress. The work adds modestly to our understanding of flow-associated vascular remodeling.

The authors have satisfactorily addressed most of my concerns with the initial submission, and have added a substantial number of figures in response to all reviews. However, a concern remains regarding presentation of migration data.

SUGGESTIONS TO AUTHORS

© 2026. Published by The Company of Biologists under the terms of the Creative Commons Attribution License (https://creativecommons.org/licenses/by/4.0/).

Major comments

1. I appreciate that the authors now present rose plots for cell migration data (Fig 5, new Fig S4), but I am still struggling to interpret the data. The authors say that the plots represent the "coordinates and relative extent of tip and SIVP migration" (as stated in the text) or "magnitude of endothelial cell movements" (as stated in the rebuttal). But I think that the units of the concentric rings must be number of migration steps within the angle bin and not distance traveled. Please explicitly define the units in text and legends. Also, based on the presented data and text and assuming that the time frame of the experiment was 56-72 hpf, I expected to see more migration steps in the dorsal direction (assumed to be centered on 90 degrees), at least in the control tip cells, but I see almost none. Relatedly, panel J shows "increased reverse migration" in control (tip and SIV); I am not sure whether this means migration against flow or dorsal migration, which would be the same thing for tip but not SIVP cells. I am struggling to understand how the rose plots can show almost no dorsal migration yet the reverse fraction graph depicts means of over 85% in all groups. As suggested by panels K and L, if all tracked cells are moving ventrally (likely because of large scale morphogenetic movements during embryo development), then you won't fully capture dorsal migration in this analysis.

We thank the reviewer for this careful assessment of the migration analysis. We agree that the description of the rose plots was insufficiently clear. We have now revised the Methods, Results and figure legends to explicitly state that the radial scale of each rose plot represents the number of single-frame migration steps falling within each angular bin, rather than distance travelled. Each migration step is defined as the displacement between consecutive tracked nuclear positions, and each petal represents the frequency of such steps within a 22.5° angular bin. Thus, the rose plots show the relative frequency of directional migration steps, not the cumulative distance travelled in each direction.

We also agree that the term “reverse migration” was potentially ambiguous. In the previous version, “reverse fraction” did not refer to migration against blood flow. Instead, it was calculated relative to a user-defined migration axis. For the analysis shown, the ventral migration axis was used as the reference axis; therefore, reverse migration refers to the fraction of migration steps opposing this ventral axis. We have now clarified this in the Methods and figure legends and have revised the Results text to avoid implying that this metric necessarily represents migration against blood flow.

The reviewer is also correct that large-scale ventral morphogenetic movement during SIVP development means that the rose plots do not capture sprout regression simply as a large number of purely dorsal steps. Rather, they show the directional distribution of stepwise EC displacements superimposed on the overall ventral expansion of the plexus. We have therefore revised the text to make clear that these analyses measure directional bias in stepwise EC migration, rather than net dorsal displacement into the SIV. The conclusion has been softened accordingly. The data support altered directional bias and clustering of tip cells in the absence of flow, consistent with impaired sprout regression.

To improve clarity, we have updated the Figure 5 and Figure S4 legends to define the angular orientation of the plots, indicate the direction corresponding to ventral migration, and specify the time window over which migration steps were analysed.

Minor comments

1. Former point 3, current lines 98-99: The lumen is the interior of the vessel that is bound by but exclusive of the endothelium. Perhaps change from "lumen of the SIV" to "lumenized SIV."

We agree and have changed this wording (line 97).

2. Figure 5 the legend still does not indicate timing of experiment

We have now added this to the legend.

© 2026. Published by The Company of Biologists under the terms of the Creative Commons Attribution License (https://creativecommons.org/licenses/by/4.0/).

3. Figure S6 and movie 7 track nuclei in a cytoplasmic line, fli1a:EGFP. How well can nuclei be distinguished in this line? Some justification is required for the use of this line versus a nuclear line.

Although GFP in *Tg(fli1a:egfp)y1* is not nuclear-localised, Quillien *et al.* showed that nuclei from this line retain sufficient GFP fluorescence to distinguish GFP-positive endothelial nuclei from GFP-negative nuclei and used Fluorescence-Activated Nuclear Sorting to isolate GFP-positive nuclei for ATAC-seq (Quillien *et al.*, 2017). In the experiments shown in Fig. S6 and Movie 7, we used embryos carrying both *Tg(fli1a:egfp)y1* and *Tg(fli1a:AC-TagRFP)sh511*. This combination enabled nuclei to be identified clearly, because nuclei were visible in the *fli1a:EGFP* channel and excluded from the membrane/cortical actin-associated AC-TagRFP signal. Tracks were generated only where nuclear positions could be followed unambiguously over time. We have now clarified this point in the Figure S6 legend.

Reviewer 3: SUMMARY OF THE ADVANCE MADE IN THIS PAPER AND ITS POTENTIAL SIGNIFICANCE TO THE FIELD

In their revised manuscript, Chen et al. have addressed most of my comments. However, there remain several related issues that still need to be cleared up.

SUGGESTIONS TO AUTHORS

Line 247. "Circulation progressed from anterior to posterior. . ." This is a bit confusing since I believe (and according to schematics in 1C'-F') flow itself ends up going the opposite direction. I think a more accurate description would be the "onset of circulation (or perfusion?)" progresses from anterior to posterior.

We thank the reviewer for this clarification. We agree that “circulation progressed” could be confused with the direction of blood flow within the SIV. We have revised the text to state that the onset of SIVP perfusion progresses from anterior to posterior (lines 272-274).

Lines 452-458. I believe that the Flt4 mutant is used for all of the analysis, so the MO data could probably be left out.

We thank the reviewer for this suggestion. We agree that the *flt4qmc316* mutant provides the main genetic evidence in this section. However, we prefer to retain the *flt4* morpholino experiment because it provides an orthogonal perturbation of *flt4* function and produces a comparable phenotype under reduced-viscosity conditions. We believe this strengthens the conclusion that reduced Flt4 activity impairs sprout regression in this context, while the revised text makes clear that the mutant analysis is the primary evidence.

Lines 463-467. Based on comparison to the cited literature (Hogan, Shin), the qmc316 allele is likely to be a hypomorph, as it does not result in lymphedema, which is clearly observed with null alleles. This should be explicitly stated.

We agree with the reviewer. Based on comparison with previously described *flt4* null alleles, and because *flt4qmc316* mutants do not develop overt oedema, we now state that *flt4qmc316* is likely to be a hypomorphic allele (lines 495-497).

Line 464. The presence of lymphedema in flt4 mutants has been correlated to the presence/absence of the main facial lymphatic vessel and valve, which looks like it is intact in the qmc316 mutant. This is not well-described here - the figure legend is vague. Zoomed-in views of thoracic duct and facial lymphatic network, and more explicit labeling, would be helpful here.

To improve clarity we have now expanded the description of the *flt4qmc316* lymphatic phenotype in both the main text and figure legend, and have added zoomed-in views of the thoracic duct and facial lymphatic network to Figure S13. These images show that *flt4qmc316* mutants have a severe reduction of lymphatic structures, including loss of the thoracic duct, while retaining some craniofacial lymphatic structures (lines 489-495).

© 2026. Published by The Company of Biologists under the terms of the Creative Commons Attribution License (https://creativecommons.org/licenses/by/4.0/).

Line 471. Please label the gaps caused by pigment cells with an asterisk. Also, there does not seem to be any mention of using the nacre mutant, though that was included in the author rebuttal. Please include the use of nacre in the methods section.

We have now added the use of the *nacre* background to the methods (line 107) and have labelled pigment-associated gaps with asterisks in figure 7

Line 483-484. Similar issue as above for characterization of lymphatic defects in MAZ51-treated embryos. Authors need to provide a better analysis here (zoomed in views, labeling particular lymphatic structures). Also, it seems the MAZ51 dose is a bit high - the density of the brain blood vessels labeled by kdrl:mcherry is clearly reduced, suggesting it may be blocking inhibiting Vegfr-2-related receptors. The authors need to use a dose that inhibits thoracic duct formation, without affecting blood vessels.

We thank the reviewer for raising this concern. We agree that, at higher doses, MAZ51 could potentially affect additional VEGFR-related pathways, including VEGFR2/Kdrl-related signalling. We have therefore strengthened the analysis of MAZ51-treated embryos and revised the text accordingly.

To better characterise the lymphatic phenotype, we have added zoomed-in views and clearer labelling of affected lymphatic structures in MAZ51-treated embryos, including the thoracic duct and craniofacial lymphatic network. We have also added analysis of cranial blood-vessel patterning in MAZ51-treated embryos at the analysed timepoint (Fig. S15F-J). This analysis did not detect a significant reduction in cranial blood-vessel patterning under the conditions used, suggesting that MAZ51 treatment caused lymphatic defects without gross disruption of the blood vascular network at this stage. These data have been added to Figure S15, and the figure legend and main text have been revised accordingly (lines 516-523).

Lines 499-500. It is possible that Flt4 is acting in a ligand independent manner in the context of a "set point" - this would be consistent with VEGFR2 acting in a ligand-independent manner with PECAM and CDH5 (as cited by the authors) as a hemodynamic sensor. Therefore, it is important that the Vegfc F0 CRISPR experiment mimic a null. Onset of lymphedema in vegfc and flt4 mutants occurs after 72 hpf, so this should not be a concern. The functional level of knockout is not clear from the data presented here. It is important to assess the SIV network using a Vegfc CRISPR dose that gives nearly fully penetrant lymphedema.

We thank the reviewer for this important point. We agree that, without a stable *vegfc* mutant, the *vegfc* G0 experiment needs to be interpreted cautiously, particularly because stronger G0 CRISPR conditions can introduce secondary developmental or haemodynamic effects.

Our *vegfc* dgRNP approach induced detectable *vegfc* mutations and impaired craniofacial lymphatic development, consistent with reduced Vegfc/Flt4 signalling. In our initial titration, a lower-dose *vegfc* dgRNP condition impaired lymphatic development without causing overt oedema, developmental delay or disruption of SIVP morphology. Under these conditions, we did not observe a significant increase in retained leading sprouts.

To address the reviewer's concern that the *vegfc* perturbation should more closely approximate a null condition, we also analysed embryos injected with a higher *vegfc* dgRNP dose and have included a figure below showing these compared to the lower dose data. This stronger perturbation caused a more severe phenotype and significantly increased leading sprout retention at 72 hpf. However, it also substantially reduced erythrocyte number within the SIV. Although aortic blood-flow velocity was not significantly reduced across the group, the response was highly variable, with many embryos showing reduced flow. Together, these observations suggest that the higher-dose condition affects local SIV formation and/or perfusion. We therefore cannot determine whether the increased leading sprout retention in this condition results directly from reduced Vegfc availability or indirectly from altered local blood flow.

For this reason, we have included the lower-dose *vegfc* G0 condition in the manuscript, as this allowed us to assess SIVP remodelling after partial *vegfc* disruption without overt developmental delay or clear secondary effects on local perfusion. We have revised the Results and in the

© 2026. Published by The Company of Biologists under the terms of the Creative Commons Attribution License (https://creativecommons.org/licenses/by/4.0/).

discussion we state explicitly that this experiment does not represent a complete *vegfc* null and does not exclude a role for residual or circulating Vegfc ligand in Flt4-dependent sprout regression. We now present the *vegfc* data more cautiously: partial *vegfc* disruption, sufficient to impair lymphatic development, does not phenocopy the reduced-flow *flt4* phenotype, but these data do not provide definitive evidence for ligand-independent Flt4 activity.

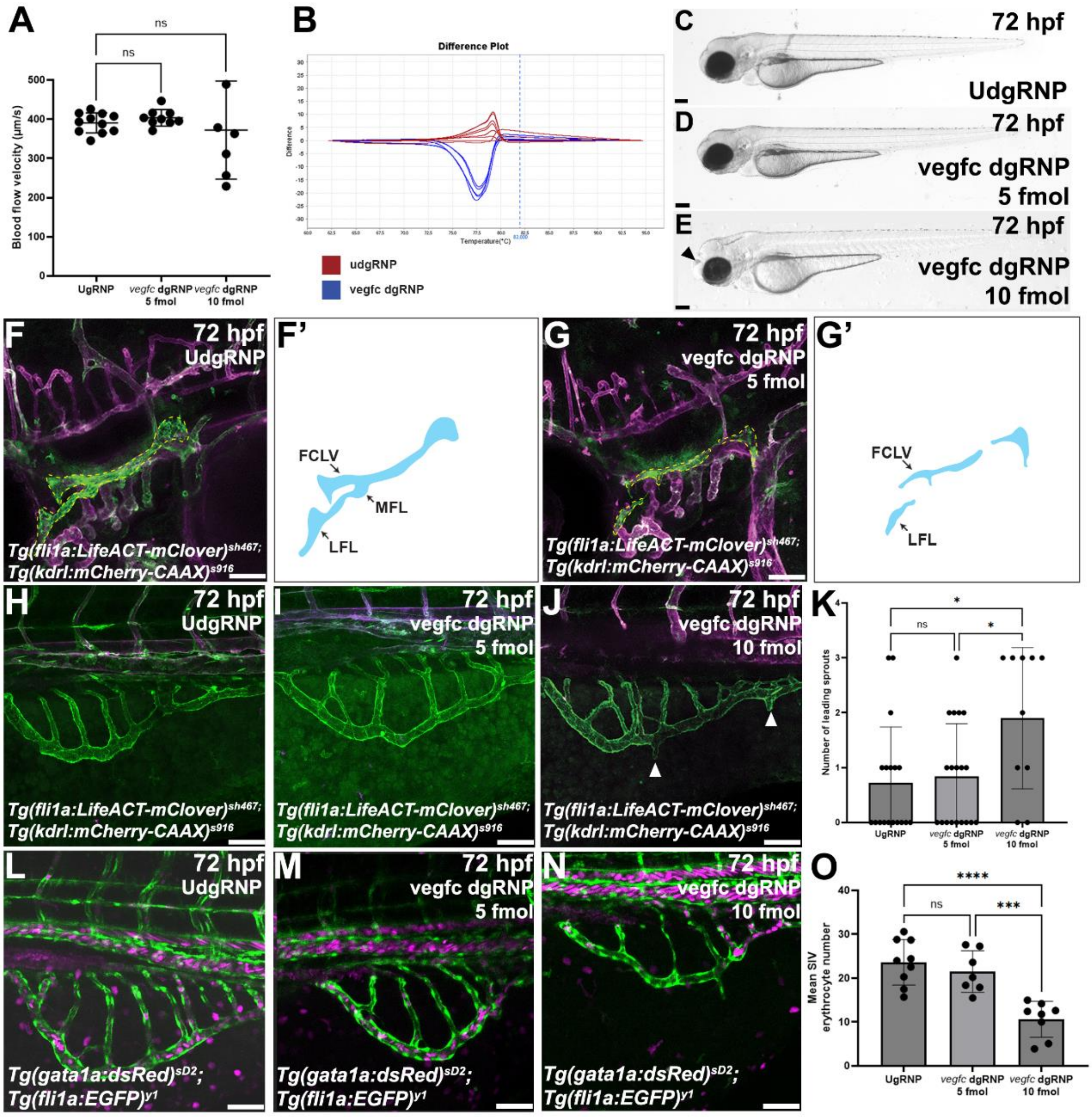

A) *vegfc* dgRNP injection does not significantly alter blood flow velocity. Unpaired *t*-test; ns, not significant. Two independent repeats; uninjected/control gRNP, n = 11; *vegfc* gRNP, n = 9; **B)** High-resolution melt analysis confirms genomic mutation in *vegfc* gRNP-injected embryos; **C-E) 5 fmol-injected** *vegfc* crispants do not display oedema or gross morphological defects compared with control gRNP-injected embryos. 10 **mol-injected** *vegfc* crispants display cranial haemorrhage (black arrowhead); **F–G')** *vegfc* crispants display impaired facial lymphatic formation compared with controls, confirming effective disruption of *vegfc* function. FCLV, facial collecting lymphatic vessel; MFL, medial facial lymphatic vessel; LFL, lateral facial lymphatic vessel; **H-J)** SIVP morphology is comparable between control and *vegfc* crispant embryos; **I)** Leading SIVP sprout number is not significantly different between control and 5 fmol *vegfc* crispant embryos but is significantly increased in 10 fmol *vegfc* crispant embryos. One-way ANOVA; *p=<0.05; ns, not significant. Two independent repeats; universal control non-targeting dgRNP (UdgRNP), n = 19; *vegfc* gRNP 5 fmol, n = 20; *vegfc* gRNP 10 fmol, n = 10. **L-N)** 10 fmol *vegfc* crispant embryos, but not 5 fmol *vegfc*

© 2026. Published by The Company of Biologists under the terms of the Creative Commons Attribution License (https://creativecommons.org/licenses/by/4.0/).

crispant embryos, display significantly reduced numbers of erythrocytes in the SIV compared to controls. One-way ANOVA; ****p=<0.0001; ***p=<0.001; ns, not significant. Two independent repeats; universal control non-targeting dgRNP (UdgRNP), n = 9; *vegfc* gRNP 5 fmol, n = 7; *vegfc* gRNP 10 fmol, n = 8. Scale bars: 100 μm in C, D; 50 μm in F, G, H, I, J, L, M, N.

Third decision letter

MS ID#: dev.205424R2

MS TITLE: Blood flow coordinates collective endothelial cell migration during vascular plexus formation and promotes angiogenic sprout regression via vegfr3/flt4

AUTHORS: Yan Chen; Aaron Michael Savage; Zhen Jiang; Ignacio Rodriguez-Pastrana; Myles Wilson; Hyejeong R. Kim; Paul C. Evans; Robert N. Wilkinson

Dear Dr Wilkinson,

I am happy to tell you that your manuscript has been accepted for publication in Development, pending our standard publication integrity checks.

© 2026. Published by The Company of Biologists under the terms of the Creative Commons Attribution License (https://creativecommons.org/licenses/by/4.0/).
